# Chemical Engineering of Transcription Factors Uncovered Cell-Permeable μMax Modulators

**DOI:** 10.1021/jacs.5c13964

**Published:** 2025-10-14

**Authors:** Omer Harel, Ferran Nadal-Bufi, Raj V. Nithun, Yumi Minyi Yao, Ariel Afek, Marc Vendrell, Muhammad Jbara

**Affiliations:** † School of Chemistry, Raymond and Beverly Sackler Faculty of Exact Sciences, Tel Aviv University, Tel Aviv 69978, Israel; ‡ Centre for Inflammation Research and IRR Chemistry Hub, Institute for Regeneration and Repair, 26745 The University of Edinburgh, Edinburgh EH16 4UU, U.K.; § Department of Chemical and Structural Biology, 34976 Weizmann Institute of Science, Rehovot 7610001, Israel

## Abstract

Transcription factor engineering has emerged as a powerful strategy for generating novel proteins for fundamental research and biomedical applications. Although various analogs have been developed, they remain largely constrained to native sequences and structures. The generation of advanced analogs bearing noncanonical modifications with enhanced functional properties remains limited. Here we combined rational design with total synthesis to engineer novel abiotic transcription factors with enhanced stability and cell permeability. Using solid-phase synthesis and native chemical ligation, we created a library of 30 Max-derived transcription factor analogs incorporating novel modifications, such as sequence mutations and aromatic staples at strategic sites. Through DNA-binding analysis and cellular uptake studies, we identified the **μMax20** analog, which contains two mutations (Lys31 and Lys57 to hArg) and exhibits potent DNA binding to the canonical enhancer box (E-box) as well as intrinsic cell permeability. Notably, further site-specific modifications of **μMax20** with aromatic staples yielded improved analogs with enhanced stability and remarkable cellular delivery at nanomolar concentrations. Our lead **μMax20** analog suppressed Myc-driven gene expression, as demonstrated by reporter gene assays and antiproliferative activity against Myc-dependent cancer cells. Altogether, these results highlight how combining chemical protein synthesis with late-stage modifications can be leveraged to enhance protein function and engineer novel bioactive modulators.

## Graphical abstract

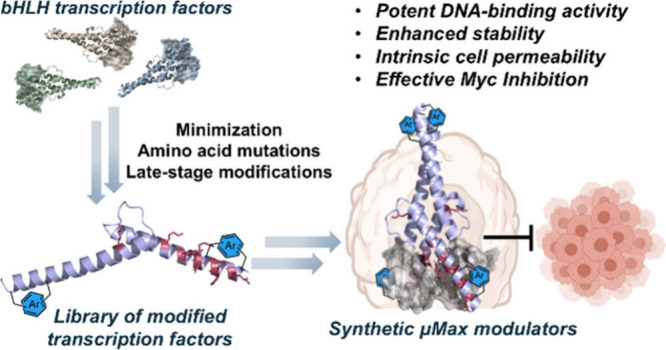

## Introduction

Nature has evolved intricate proteins to regulate the functions of living organisms. Of particular interest are transcription factors (TFs), which play key roles in modulating essential cellular processes in human health and disease. TFs are regulated by an array of mechanisms, such as post-translational modifications (PTMs), which can influence their stability, localization, and function.^1^ Inspired by nature, several synthetic TF analogs (e.g., zinc finger proteins) have been developed for basic research and biomedical applications. Although these elegant systems have opened new avenues for the development of TF modulators, the intracellular delivery of these complex macromolecules remains a significant challenge.^2^ In principle, engineering synthetic TFs with unique residues and modifications can expand their structural and chemical space, enabling the exploration of new reactivities for the development of novel protein modulators.^3^ Importantly, such modifications must preserve the protein structure to maintain functional activity. Indeed, research has shown that the rational insertion of novel modifications into polypeptides can lead to a dramatic refinement of key structural and functional features, such as folding, stability, and activity.^4^ Although these unique reactivities have been explored in peptides, the precise and high-resolution incorporation of non-natural residues or modifications into proteins remains limited and warrants further investigation.^5^ This challenge arises from the complexity of generating well-defined, site-specifically modified proteins using conventional biological methods.

Recombinant protein expression, coupled with in vitro enzymatic diversifications, can sometimes enable the production of modified proteins; however, this approach is usually restricted to the transfer of PTMs to native residues with limited selectivity.^6^ Other powerful biological approaches, such as genetic code expansion technology, can enable the insertion of a variety of novel modifications at selected sites.^7^ However, this process is technically challenging when multiple modifications are required.^8^ Alternatively, synthetic chemistry methodologies can overcome these limitations by facilitating protein editing down to the atomic level.^9^ For instance, late-stage protein modification enables effective protein diversification starting from recombinant proteins;^10^ however, this method is still limited by the type of modification and protein sequence.^11^ Importantly, the use of solid-phase peptide synthesis (SPPS) and chemoselective peptide ligations can overcome these limitations, enabling the production of modified proteins at virtually any desired site.^12^ These techniques were extensively applied to probe the molecular role of the PTMs of proteins.^13^ Despite these advances, the production of homogeneous proteins bearing novel modifications (e.g., nonproteinogenic residues and staples) remains challenging and rare.

We envisioned the combination of structure-based design with total synthesis and late-stage modifications would enable the development of novel TF analogs with refined structural and functional properties to facilitate their intracellular delivery and activity. To explore the potential of incorporating novel residues into TFs, we selected the basic-helix–loop–helix leucine-zipper (bHLH-LZ) TF Myc–Max–Mad network, which plays a pivotal role in the progression of human cancers.^14,15^ This network controls the expression of approximately 15% of the human genome by forming dimeric complexes that selectively bind to promoter regions containing the enhancer box DNA sequence (5′-CACGTG-3′, E-box) to either activate or suppress gene expression.^16^ The Myc–Max–Mad network is involved in key cellular processes such as cell proliferation, differentiation, and apoptosis. Its deregulation is implicated in many human disease pathogeneses, including various types of cancer (∼50% of human tumors).^17^

Gene expression is primarily initiated and regulated by TFs interacting with specific DNA sequences through their DNA-binding domains.^18^ DNA-binding activity and sequence specificity are mainly governed by the unique topology of the DNA-binding motif. We sought to build our abiotic TFs primarily based on the DNA-binding domain of the TF Max, which governs the dimerization and DNA-binding activity of the Myc–Max–Mad network. Crystal structure and sequence analysis revealed that six key residues make specific base pair contacts or interact with the phosphate backbone to bind to the canonical E-box DNA sequence.^16^ We anticipated that the insertion of sequence mutations and modifications into key residues can potentially expand the functional space of Max to develop new analogs with refined activity. In this study, we combined structure-based design and total synthesis to develop minimized modified TF analogs derived from natural bHLH-LZ TFs, e.g., Max. We demonstrated the power of combining chemical protein synthesis and late-stage modifications to produce a library of abiotic TF analogs. Importantly, we employed our approach to prepare 30 TF analogs, with the insertion of up to six modifications at strategic positions. We identified **μMax20** analogs, bearing two mutations at strategic sites (e.g., Lys31hArg and Lys57hArg mutations) and staples, which exhibited potent DNA-binding activity, intrinsic cell permeability at submicromolar concentrations, and effectively suppressed Myc-driven gene expression and cancer cell proliferation. Taken together, our findings demonstrate the power of combining structure-based design and total synthesis to develop novel proteins with tailored functional properties.

## Results and Discussion

### Structure-Based Design of Abiotic Transcription Factors Derived from the Myc–Max–Mad Network

The Max TF plays a key role in regulating gene expression by forming transcriptionally active (Myc/Max) and repressive (Mad/Max) protein dimers through partner selection mechanisms.^15^ We envisioned that rational engineering of the Max TF would render advanced bioactive analogs able to modulate Myc-driven gene expression upon cellular delivery by antagonizing its binding to the canonical E-box (Figure 1). We first sought to explore the impact of strategically inserting sequence mutations in the structure and function of Max. Toward this goal, we derived the design of the abiotic TF analogs from the DNA-binding domain of Max to retain functional TF homodimers for specific DNA-binding to the core E-box site (Figure 2). The X-ray structure of the Max/Max-DNA complex revealed six key residues within the basic region that interact with the E-box sequence: Arg16, His19, Asn20, Glu23, Arg24, and Arg27 (Figure 2).^16^ We envisioned that incorporating potential nonproteinogenic amino acids at these sites might augment vital TF-DNA interactions, such as hydrogen bonds and electrostatic interactions, consequently refining DNA-binding activity toward the target E-box sequence. This expansion of the chemical and interaction space would provide novel opportunities to study the impact of non-natural functionalities on the DNA-binding activity and function of abiotic TFs compared to their natural counterparts.

**Figure 1:**
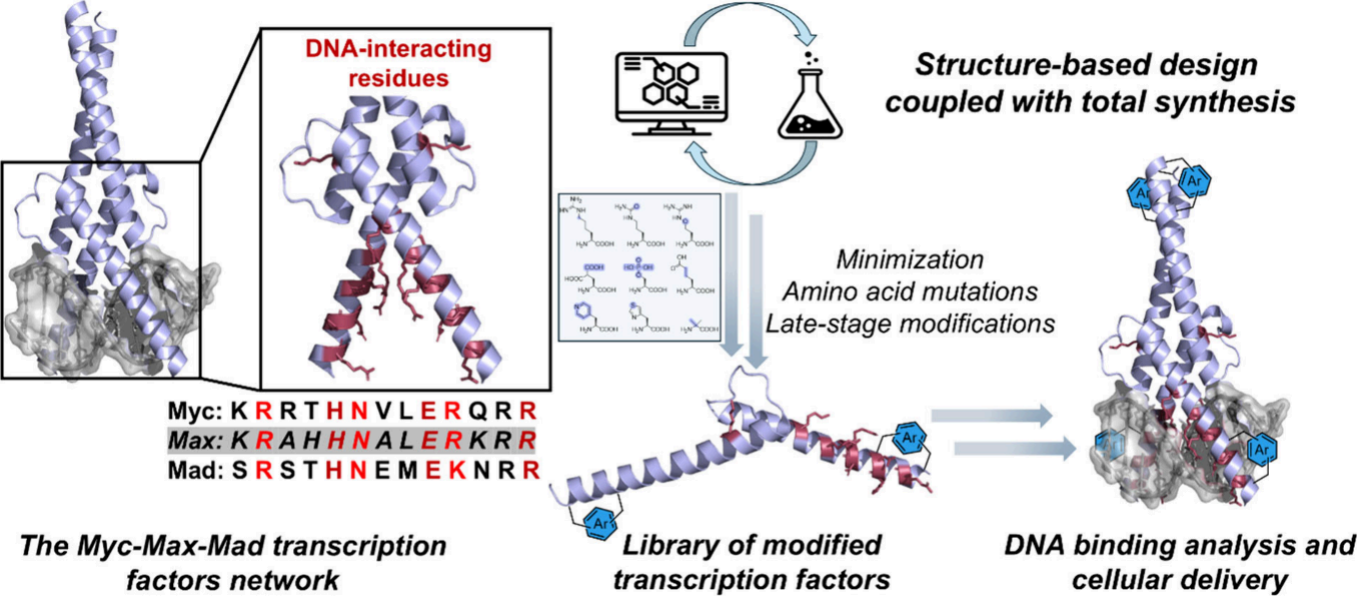
Design of abiotic transcription factors derived from the Myc–Max–Mad network (PDB ID: 1HLO).

**Figure 2:**
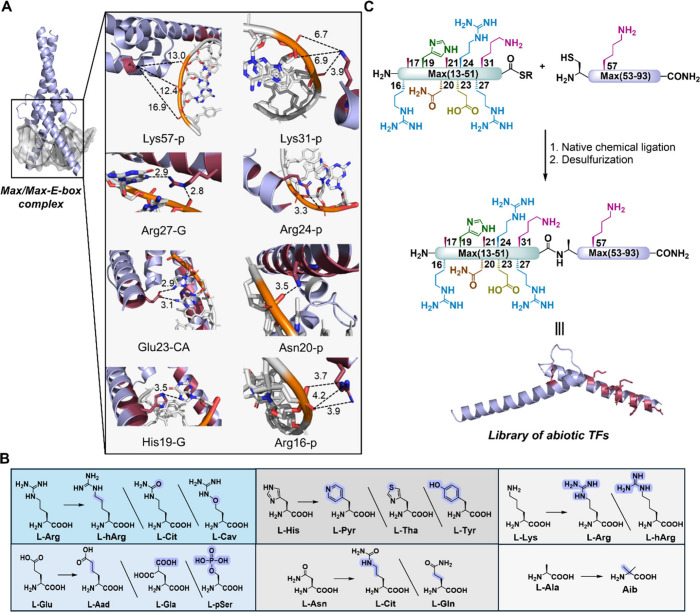
Design of abiotic TF by the strategic insertion of sequence mutations into the DNA-binding domain of Max. (A) The interactions of Max’s residues with the core E-box (PDB ID: 1HLO). p denotes DNA’s phosphate backbone; G, C, and A are guanine, cytosine, and adenine, respectively. (B) Canonical and noncanonical residues that were used in this study to replace native amino acids. (C) Schematic representation of the chemical synthesis of a library of abiotic Max analogs using a one-pot native chemical ligation and desulfurization approach.

To examine our hypothesis, we first sought to incorporate noncanonical amino acids at key residues in the basic DNA-binding domain of Max to optimize the length and electronic properties of strategic residues that contact the E-box DNA sequence and probe the DNA-binding activity (Figure 2A). For instance, Arg can be replaced with l-homoarginine (hArg), which has an extra methylene group in its alkyl chain, and l-citrulline (Cit) which features a urea functional group instead of guanidine (Figure 2B). Another alternative is l-canavanine (Cav), where the δ-carbon is replaced by an oxygen atom, introducing further diversity. The side chain of Glu can be substituted by l-homoglutamic acid (Aad), γ-carboxy-Glu (Gla), or phospho-serine (pSer), thus manipulating the length and electronic properties of its side chain. His also offers a versatile target for substitution with analogs like l-Ala­(4′-pyridyl) (Pyr) or l-Ala­(4-thiazolyl) (Tha) to modify its ring size and electronic properties. Asn can be substituted for Cit to lengthen its side chain. Finally, potential Ala sites can be replaced by α-aminoisobutyric acid (Aib) to induce the helicity of the DNA-binding domain. In addition to the nonproteogenic amino acids, we also included natural residues (e.g., His to Tyr, Asn to Gln, and Lys to Arg), which can result from genetic mutations, to assess the impact of these changes on the TF-DNA interactions.

We have recently found that post-translational modifications of Max can dramatically influence its DNA-binding activity.^19^ Importantly, we found that the acetylation of Lys31 and Lys57 significantly reduces the DNA-binding affinity of Max to the E-box, potentially due to the disruption of essential salt bridges between the ammonium residue and the phosphate diester backbone. Both residues have no direct contact with the E-box nucleotides but are proximal to the phosphate DNA backbone. We hypothesized that increasing the positive charge density and extending the length of the side chains, using Arg or hArg amino acids, might contribute to the DNA-binding activity of Max. In total, our design includes manipulating 10 strategic sites within Max’s sequence with 12 novel residues, of which 9 are nonproteinogenic amino acids and 3 are natural residues (Figure 2).

### Synthesis of a Library of Abiotic Transcription Factors by Strategic Insertion of Non-proteinogenic Residues

To synthesize a minimized Max version, we initially divided the DNA-binding domain of Max(13–93) into two peptide segments (Figure 3A): Max(13–51)-NHNH_2_ and Cys-Max(53–93).^20^ Ala52 was temporarily mutated into Cys to allow native chemical ligation (NCL)^21^ at this site, which could be converted back to Ala after NCL via a final desulfurization step.^22^ All peptide segments were prepared in parallel using stepwise Fmoc-SPPS with the selective incorporation of the desired single-point mutation, as described in Figure 3B (SI section 4), using commercially available amino acids.^23^ We obtained 18 Max(13–51)-NHNH_2_ segments (**1**–**18**) and 3 Cys-Max(53–93) segments (**19**, **21**, and **22**) on a milligram scale in an average of 13% isolated yields after RP-HPLC purification (SI section 4).

**Figure 3:**
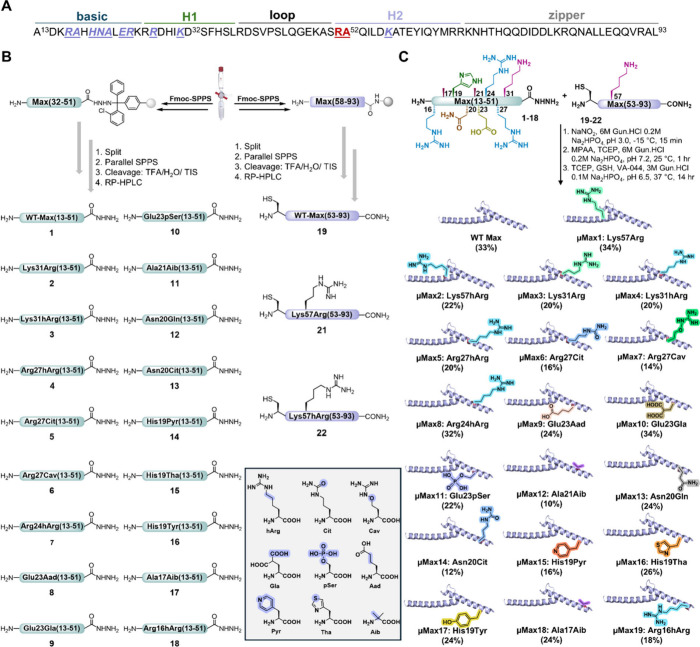
Chemical synthesis of a library of abiotic TF analogs using native chemical ligation. (A) Max(13–93) sequence (p21 isoform). The ligation site is highlighted in red, and amino acids that were mutated are highlighted in purple. Asp32 is the last residue incorporated prior to resin splitting for the parallel SPPS of the hydrazide segment. (B) Schematic representation of the chemical synthesis of **μMax** segments using Fmoc-SPPS. (C) Schematic representation of the synthesis of **μMax** analogs via one-pot NCL desulfurization (PDB ID: 1HLO).

Having all segments, we ligated each Max(13–51)-NHNH_2_ segment (**1**–**18**) with the Cys-Max(53–93) partner (**19**, **21**, or **22**) under standard NCL conditions (Figure 3C).^24^ In brief, Max(13–51)-NHNH_2_ segments (**1**–**18**) were converted into acyl-azide using NaNO_2_ in 6 M guanidine hydrochloride (Gun·HCl), 0.2 M Na_2_HPO_4_ buffer, pH 3.0 at −15 °C.^25^ Then, 4-mercaptophenylacetic acid (MPAA) was added for in situ thioesterification, followed by the addition of relevant peptide segments (**19**, **21**, or **22**) in the presence of tris­(2-carboxyethyl)­phosphine (TCEP).^26^ The reaction pH was adjusted to 7.2 and kept at room temperature for 1 h (SI section 5). The progress of the ligation reaction was analyzed by LCMS. Subsequently, the crude ligation reaction was desalted to enable a one-pot desulfurization reaction in the presence of the radical initiator 2,2′-azobis­[2-(2-imidazolin-2-yl)­propane]­dihydrochloride (VA-044), TCEP, and glutathione (GSH) to convert Cys52 at the ligation junction to the native Ala (SI section 5).^27^ Following the RP-HPLC purification step, all mutated Max (**μMax1**–**19**) analogs were obtained in 10–34% isolated yields after the four synthetic steps. In addition, following the same synthetic procedure, we synthesized the wild-type Max(13–93) analog (**WT Max**) as a control. The identity and purity of all 20 synthetic Max analogs were confirmed by LCMS (Figures S10–S12). Importantly, these results demonstrate the versatility and flexibility of total synthesis to produce novel TF analogs bearing engineered residues, underscoring the potential of this approach to prepare libraries of homogeneous abiotic proteins for various applications.

### DNA-Binding Analysis and Biophysical Characterization of the Synthesized **μMax** Analogs

Synthetic **μMax** analogs are functional and capable of binding to the canonical E-box DNA sequence. We initially assessed the DNA-binding activity of the **μMax1**–**19** analogs with a double-stranded DNA probe containing the canonical E-box sequence via an electrophoretic mobility shift assay (EMSA). After incubating all analogs with the E-box probe separately, we found that the synthesized **μMax** analogs were associated with the E-box DNA probe, as indicated by an upward shift of DNA (Figure 4). Interestingly, we observed that mutating Lys57 and Lys31 to hArg/Arg enhanced their DNA-binding activity. This can be attributed to the extended side-chain length of hArg/Arg and their delocalized positive charges, which potentially stabilize the μMax–DNA complex through the guanidinium and phosphodiester interactions. Additionally, both mutations of Ala17 and Ala21 to Aib resulted in gain-of-function, potentially by contributing to the helicity of the basic domain. On the other hand, we found that mutations at core residues that directly interact with DNA bases generally led to a reduction in activity.^28^ Notably, an exception was observed at His19, where mutating it to Tha or Tyr improved the DNA-binding activity. Overall, these experiments revealed that single mutations in **μMax** analogs are functional, but with variations in their binding to the E-box DNA, highlighting the critical role of the mutation sites on DNA-binding activity. Altogether, mutations in residues that directly interact with the E-box nucleotides led to a reduction in DNA-binding activity, whereas mutations in other strategic residues, e.g., Ala17/21 or amino acids that can interact with the DNA backbone, e.g., Lys31/57 can enhance DNA binding (Figure 4).

**Figure 4:**
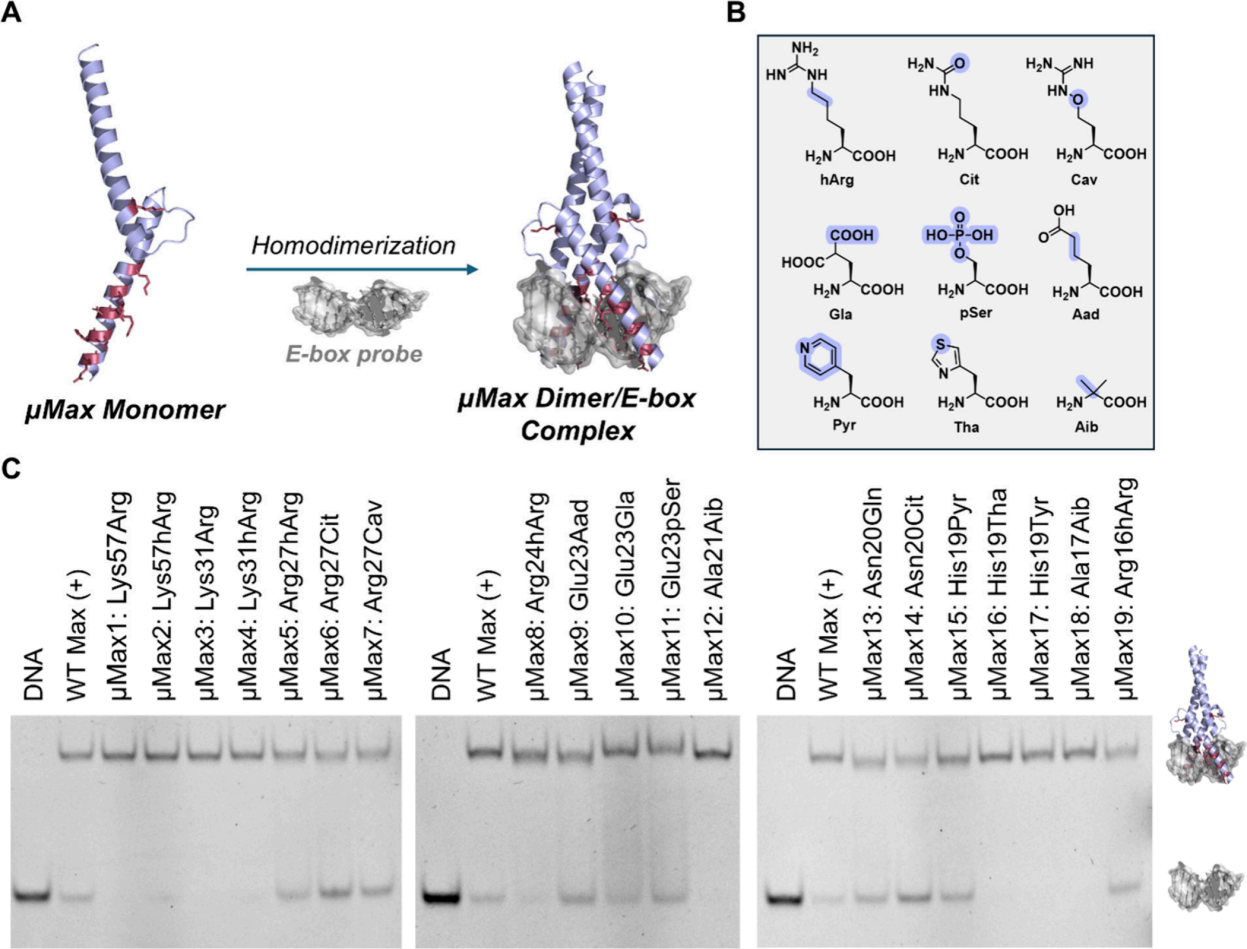
Synthetic **μMax** analogs are functional and capable of binding to the core E-box DNA. (A) **μMax** dimerization and DNA binding (PDB ID: 1HLO). (B) Noncanonical amino acids repertoire. (C) EMSA experiments of **μMax** analogs. Conditions: 1 μM DNA probe and 3 μM **μMax** analog incubated in 10 mM MES, 150 mM KCl, 1 mM MgCl_2_, and 10% glycerol buffer (pH 6.0) at room temperature. Experiments were performed in duplicate.

The mutation site in Max TF is critical for DNA sequence specificity. Next, we focused on combining productive mutations to produce advanced **μMax** analogs bearing 2–5 mutations (Figure 5). To this end, we synthesized three **μMax** analogs with combined mutations to probe their DNA-binding activity: Lys57hArg and Lys31hArg (**μMax20**); Lys57hArg, Lys31hArg, Ala21Aib, and His19Tha (**μMax21**); Lys57hArg, Lys31hArg, Glu23Aad, Ala21Aib, and His19Tha (**μMax22**). All three analogs **μMax20**–**22** were prepared by employing the one-pot NCL-desulfurization approach described in Figure 3 (Figure 5A and SI section 5). Subsequently, we incubated each of the **μMax20**–**22** analogs separately with the E-box probe and analyzed their DNA-binding activity via EMSA (Figure 5B). The assays revealed that **μMax20** and **μMax21** exhibited potent DNA-binding activity, whereas **μMax22** showed a slightly reduced binding activity. We further confirmed that inserting sequence mutations in our lead analogs (**μMax20** and **μMax21**) did not interfere with the secondary structure of Max TF. We characterized the secondary structure of **μMax20**, **μMax21**, and **WT Max** using circular dichroism (CD) spectroscopy. In these experiments, all analogs exhibited a similar α-helical pattern, with deep double minima at 208 and 222 nm, as depicted in their CD spectra (Figure 5C).

**Figure 5:**
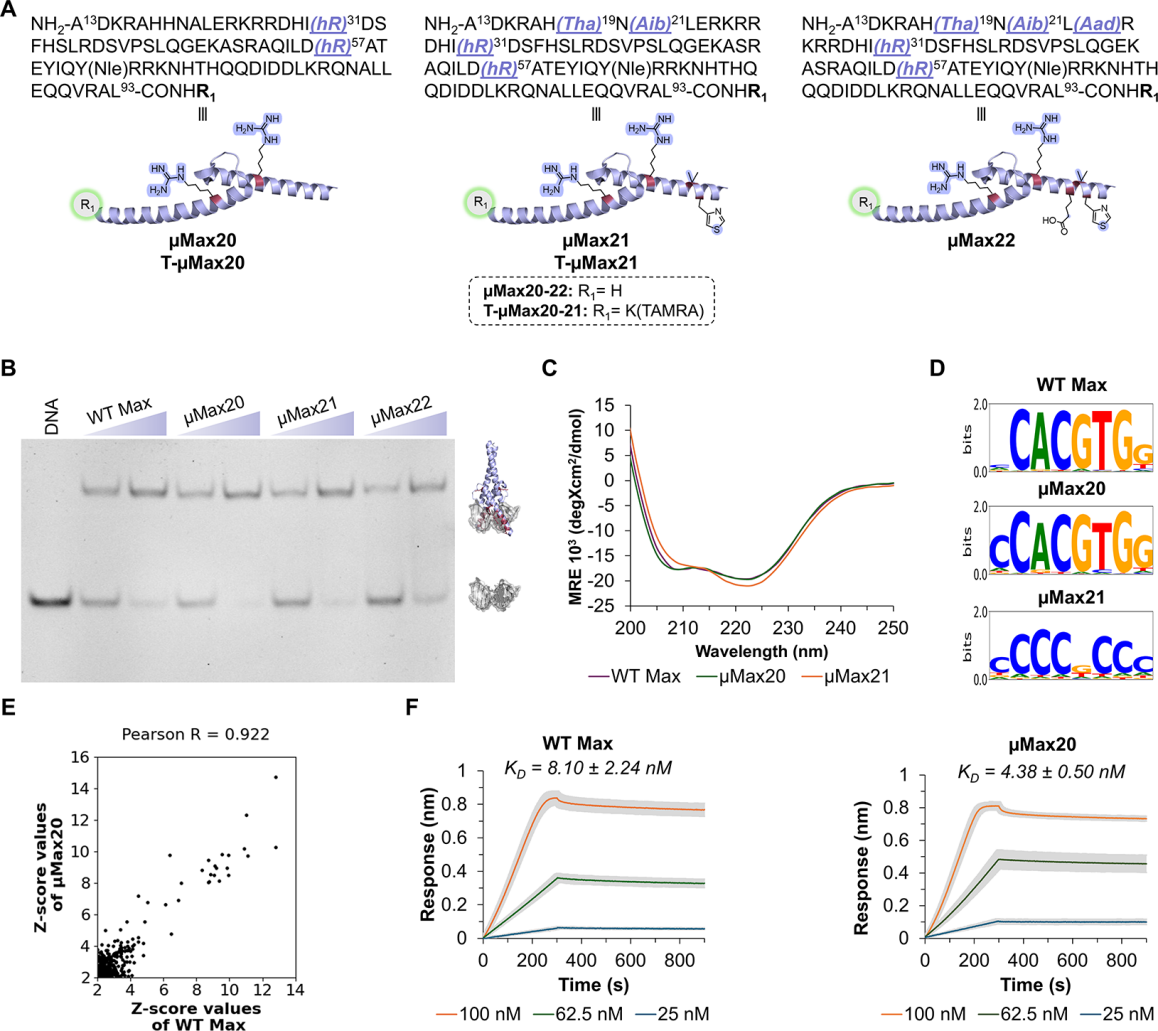
**μMax20** shows potent DNA-binding activity and sequence specificity of the core E-box DNA sequence. (A) Schematic representation of the combined mutations of the **μMax** analogs (PDB ID: 1HLO). (B) EMSA experiment of **μMax20**–**22**. Conditions: 1 μM DNA probe and 2 or 3 μM **μMax** analogs incubated in 10 mM MES, 150 mM KCl, 1 mM MgCl_2_, and 10% glycerol buffer (pH 6.0) at room temperature. Experiment was performed in duplicate. (C) CD analysis of **WT Max**, **μMax20**, and **μMax21**. Experiments were performed in triplicates. (D) Position weight matrix (PWM) logos of **WT Max**, **μMax20**, and **μMax21**. Experiment was performed in duplicates which showed high reproducibility as confirmed by the correlation of probes intensities (Figure S31). (E) Consistent trend in 8-mer sequences between **WT Max** and **μMax20**. The correlation between the 8mer Z-score values of **WT Max** and **μMax20** demonstrating similar binding preferences toward 8-mer cores for both variants. (F) Sensorgrams from a biolayer interferometry analysis of **WT Max** and **μMax20** binding to the E-box DNA probe (*K*_D_ = 8.10 ± 2.24 nM and 4.38 ± 0.50 nM, respectively; mean ± SD). Experiments were performed in triplicates.

Next, we investigated the DNA sequence specificity of our lead analogs to the core E-box sequence. More specifically, we examined the binding specificity of **μMax20** and **μMax21** across a diverse range of DNA sequences using the protein binding microarray (PBM) experiments^29^ and compared their preferences to those of the **WT Max**. The binding of the **WT Max** and the **μMax20** and **μMax21** analogs was measured on a universal DNA microarray chip, encompassing all possible 10-mer sequences (SI section 9).^29^ To quantitatively characterize DNA-binding specificity, we used the enrichment score (E-score), a standard metric in PBM analysis that ranks and thresholds k-mers by their binding enrichment.^30^ The position weight matrix (PWM) logos for **WT Max** and **μMax20** reveal strong binding preferences, and the specificity E-score of 0.497 and 0.498 further validate the strong specificity for their respective binding sequences. Importantly, **μMax20** retained the binding preferences of **WT Max** (Figure 5D, showing binding profiles), which was further confirmed by correlating the Z-scores of all 8-mers of the two proteins (Figure 5E). This comparison yielded a high Pearson R value of 0.922, indicating that sequence preferences were preserved. Further, the specificity of **μMax20** to the E-box was also confirmed by comparing probes containing the core binding site to the other sequences (SI section 9 and Figure S32). In contrast, **μMax21** showed a marked loss of selectivity for the canonical palindromic E-box sequence (Figure 5D, illustrating altered binding patterns). The loss of specificity may be attributed to the His19 mutation to Tha in **μMax21**, as this residue directly interacts with the E-box via its imidazole residue and the guanine base. We hypothesize that replacing His19 with Tha disrupts a key contact, thereby impairing E-box recognition and reducing specificity. These experiments revealed that despite sequence mutations at strategic sites of Max, our synthetic Max analog, **μMax20**, retains potent and specific DNA-binding activity with the canonical E-box binding motif. Finally, we analyzed the DNA-binding affinity of the **μMax20** analog by determining the dissociation constant to the E-box DNA probe via the biolayer interferometry (BLI) assay (SI section 10). We measured a *K*_D_ value of 4.38 ± 0.50 nM for **μMax20** and 8.10 ± 2.24 nM for **WT Max** (Figure 5F). All together, these assays demonstrate the preservation of folding and the DNA-binding activity of the abiotic Max analog **μMax20** with the target E-box sequence.

### Live-Cell Imaging Reveals Enhanced Intracellular Delivery of **μMax20** Analogs

A major challenge in the development of protein modulators is achieving efficient intracellular delivery, which may require fusion to cell-penetrating peptides or the use of specialized and invasive delivery agents. We anticipated that inserting the double hArg mutations in **μMax20** would facilitate its cellular uptake compared to the native analog.^31^ Furthermore, we envisaged that the incorporation of peptide staples at strategic positions in **μMax20** would further enhance its cell permeability.^32^ With this design in mind, we generated a stapled version of the **μMax20** analog. First, we investigated potential mutation sites and suitable stapling chemistries within the **μMax20** sequence. Specifically, we prepared two **μMax20** analogs containing dual aromatic staples that were incorporated via late-stage Cys modification using 2,6-bis­(bromomethyl)­pyridine and 1,3-bis­(bromomethyl)­benzene reagents to furnish **2S**_**p**_ **-μMax20** and **2S**_**b**_ **-μMax20** analogs, respectively (SI section 5 and Figure 6A).^33^ Next, we analyzed the DNA-binding activity of both stapled analogs via EMSA. Both analogs exhibited dose-dependent association with the DNA probe, with **2S**_**b**_ **-μMax20** showing effective binding (Figure 6B). We also evaluated the sequence specificity of both stapled analogs via PBM, which revealed that the core specificity for the E-box motifs was maintained (SI section 9 and Figure 6C). Finally, we evaluated the proteolytic stability of the stapled analogs. Both **2S**_**p**_ **-μMax20** and **2S**_**b**_ **-μMax20** displayed enhanced proteolytic stability with ∼2.5-fold longer half-lives than **WT Max** (SI section 11).

**Figure 6:**
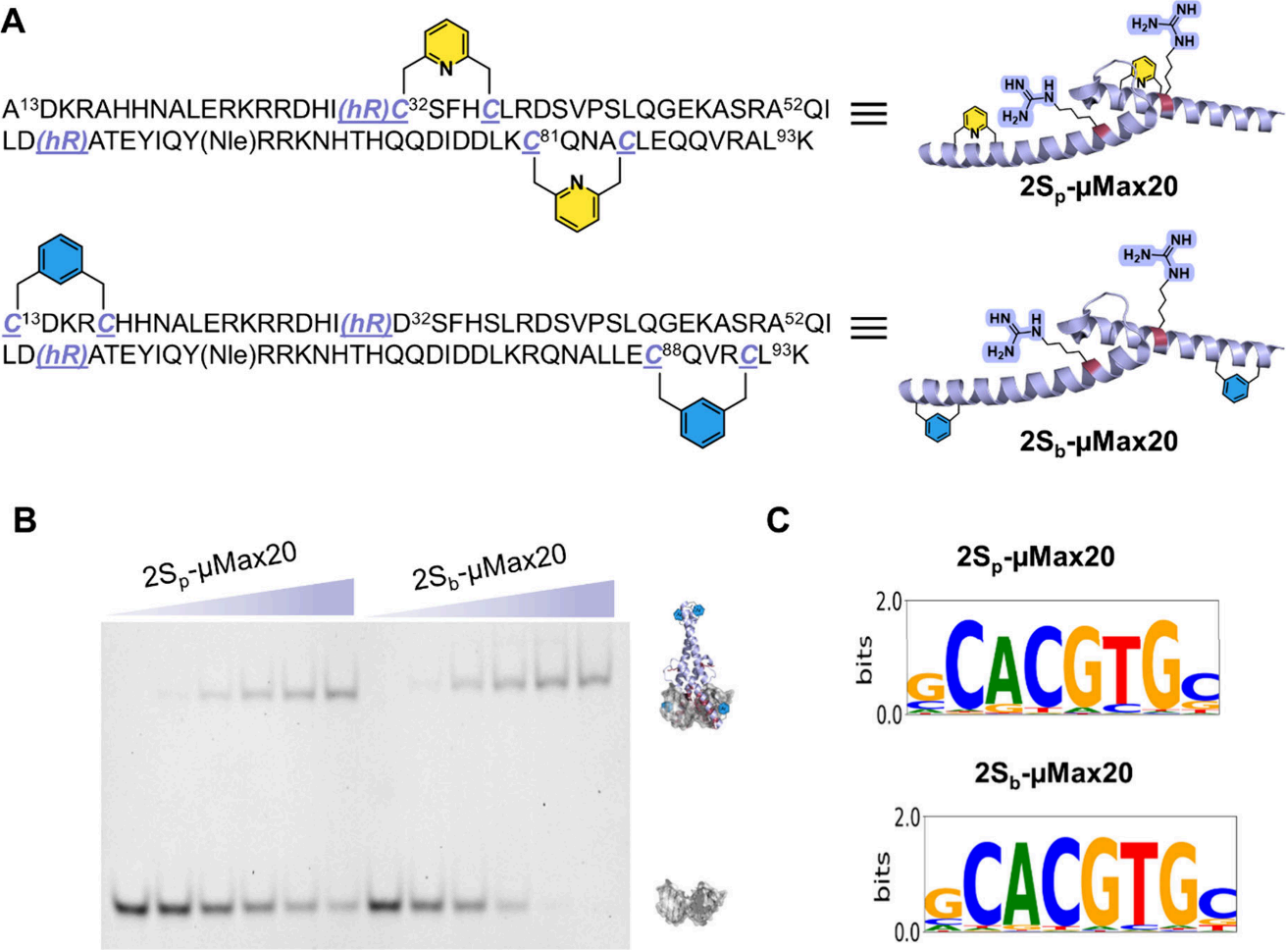
Development of stapled **μMax20** analogs. (A) Schematic representation of the stapled **μMax20** analogs (PDB ID: 1HLO). (B) Results of EMSA for **2S**_**p**_ **-μMax20** and **2S**_**b**_ **-μMax20**. Conditions: 1 μM DNA probe and 0–5 μM stapled **μMax20** analogs incubated in 10 mM MES, 150 mM KCl, 1 mM MgCl_2_, and 10% glycerol buffer (pH 6.0) at room temperature. This experiment was performed in duplicate. (C) Position weight matrix (PWM) logos of **2S**_**p**_ **-μMax20** and **2S**_**b**_ **-μMax20**; E-scores of 0.478 and 0.477 respectively, indicating strong specificity for their preferred binding sequences. This experiment was performed in duplicate (Figure S31).

To examine both the cellular uptake and localization of our developed Max analogs, we fluorescently labeled **μMax20** (**T-μMax20**) and the stapled analogs (**T-2S**_**p**_ **-μMax20** and **T-2S**_**b**_ **-μMax20)** with the TAMRA fluorophore. As controls, we used TAMRA-labeled WT Max (**T-WT Max**) and TAMRA-labeled full-length native Max (**T-Native Max**). Then, we assessed the internalization of our analogs at varying concentrations using confocal fluorescence microscopy (SI section 12). We evaluated the cellular permeability of **T-Native Max, T-WT Max, T-μMax20, T-2S**_**p**_ **-μMax20**, and **T-2S**_**b**_ **-μMax20** in HeLa cells after incubation with every analog at 0.25, 1, and 4 μM (Figure 7A,B). As expected, **T-Native Max**, which contains the full-length Max sequence and lacks modifications that can enhance permeability, did not internalize at any of the concentrations tested (Figure 7B,C). These results confirmed that TAMRA conjugation alone did not drive nonspecific cellular uptake. Next, the truncated native analog (**T-WT Max**) showed limited permeability, with intracellular fluorescence observed only at 4 μM and negligible signals at 1 μM and 0.25 μM. In contrast, **T-μMax20** exhibited markedly enhanced uptake, with bright intracellular fluorescence signals at 1 μM. The quantification of TAMRA fluorescence emission normalized to nuclear staining (green:red ratio) corroborated the increased uptake of **T-μMax20** over **T-WT Max** (0.69 ± 0.01 vs 0.33 ± 0.03, respectively; Figure 7C).

**Figure 7:**
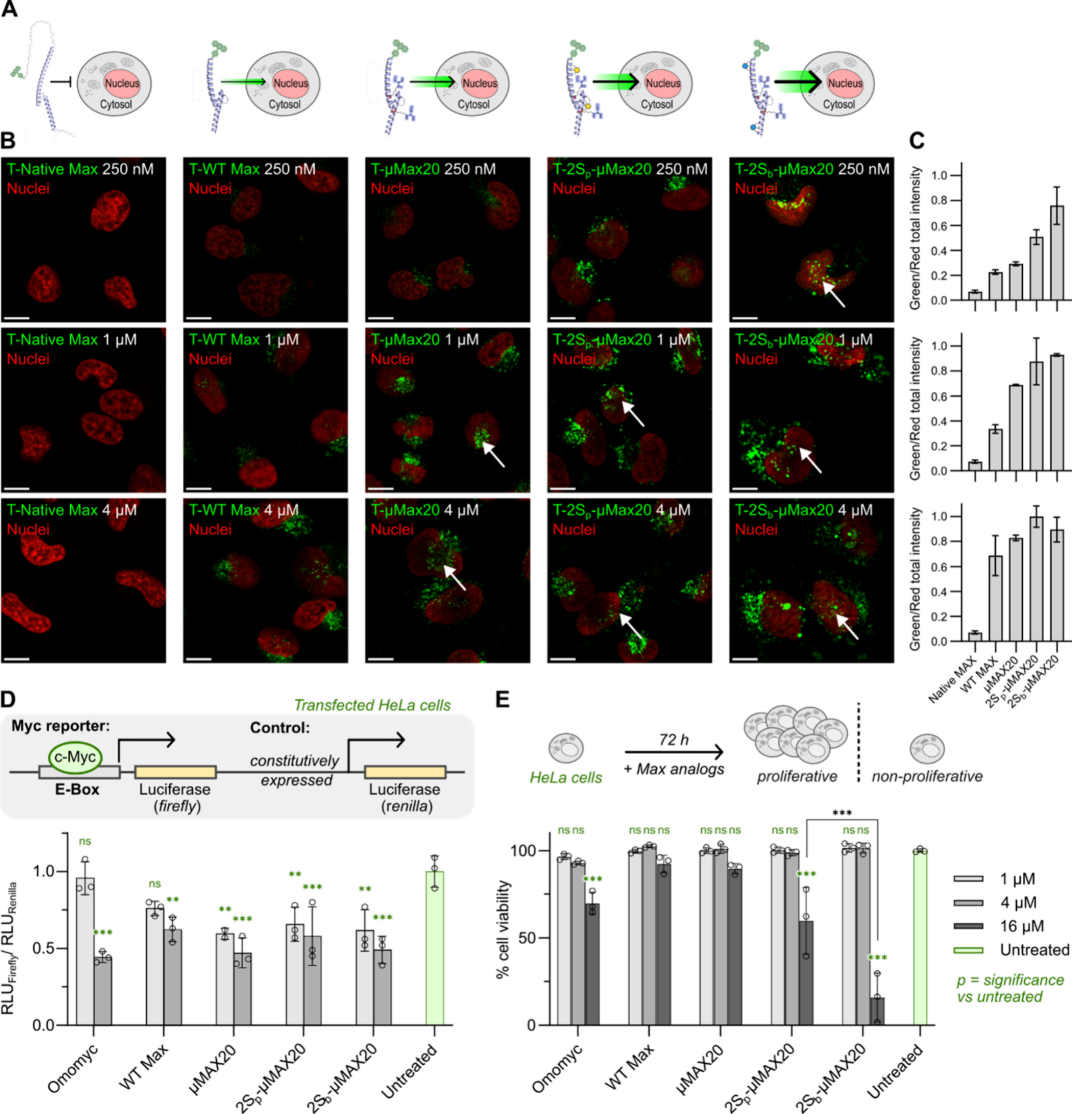
Abiotic **μMax20** analogs enhance cellular uptake and nuclear localization compared to native Max analogs. (A) Schematic overview of the tested Max analogs and their cellular permeability. The structure of **T-Native Max** was predicted using AlphaFold (PDB ID: 1HLO). (B) Representative fluorescence microscopy images of HeLa cells treated with TAMRA-labeled Max analogs (**T-Native Max**, **T-WT Max**, **T-μMax20**, **T-2S**_**p**_ **-μMax20**, and **T-2S**_**b**_ **-μMax20**) at increasing concentrations: 250 nM (top), 1 μM (middle), and 4 μM (bottom). TAMRA fluorescence (green; exc/em 552/578 nm) visualizes protein localization, and nuclei were costained with Hoechst 33342 (red; exc/em 405/461 nm). White arrows indicate nuclear accumulation of μMax-based analogs. Figure S36 includes all brightfield microscopy images. Figures S37–S39 contain single-channel fluorescence images of TAMRA and Hoechst signals. Scale bars: 10 μm. (C) Quantification of intracellular TAMRA fluorescence normalized to Hoechst nuclear signals. Mean fluorescence values were calculated using FIJI from three independent fields of view per condition. Data are presented as means ± SD. (D) Myc transcriptional activity in HeLa cells transfected with a luciferase-based Myc reporter system and treated with Max analogs (1 or 4 μM for 48 h). Data represents the ratio of firefly luciferase expression (driven by Myc binding to E-box elements) to renilla luciferase (constitutively expressed), normalized to untreated cells. Data shown as means ± SD from three independent experiments. (E) Cell viability of HeLa cells after incubation with Max analogs (1, 4, or 16 μM for 72 h), measured by resazurin-based assays. PBS and 0.01% (v/v) Triton X-100 were included as negative and positive controls, respectively, and used for normalization. Statistical significance relative to untreated was calculated by two-way ANOVA: *p* < 0.01 (**), *p* < 0.001 (***), p > 0.05 (ns). Data shown as means ± SD from three independent experiments.

Strikingly, the stapled analogs **T-2S**_**p**_ **-μMax20** and **T-2S**_**b**_ **-μMax20** showed efficient cellular internalization even at 250 nM, with notable nuclear accumulation at higher concentrations (Figure 7B,C). Among the two analogs, **T-2S**_**b**_ **-μMax20** displayed superior uptake with substantially brighter intracellular signals at 250 nM (green:red ratios: 0.51 ± 0.06 for **T-2S**_**p**_ **-μMax20** and 0.76 ± 0.15 for **T-2S**_**b**_ **-μMax20**). Notably, **T-2S**_**b**_ **-μMax20** also exhibited nuclear localization at submicromolar concentrations as confirmed by Z-stack fluorescence microscopy (SI section 12, Figure S40, and Movie S1). The bright fluorescent clusters in the nuclei may reflect chromatin-associated transcription factors; however, these could also be due to protein aggregation and it will be examined in future investigations. Finally, brightfield images revealed that treated cells retained normal morphology and adhesion, suggesting preserved membrane integrity and low toxicity across all analogs.

Fluorescence patterns of internalizing analogs revealed a punctate distribution (Figure 7B), particularly at lower concentrations, indicative of endosomal localization and suggesting an endocytosis-driven uptake mechanism. To confirm this, we treated the cells with the endocytosis inhibitor MiTMAB,^34^ which effectively blocked the internalization of both **T-WT Max** and **T-μMax20** (SI section 13). The cellular uptake of the stapled analogs **T-2S**_**p**_ **-μMax20** and **T-2S**_**b**_ **-μMax20** was markedly reduced but not completely abolished under the same conditions (SI section 13 and Figure S41), indicating that their internalization involves a dual mechanism of both endocytosis and direct membrane translocation. This observation is consistent with the reported behavior of stapled peptides, which are known to exploit multiple routes of cellular entry.^35^ Overall, these findings highlight the impact of the novel modifications in **μMax20** for promoting efficient cellular uptake and nuclear localization. The development of intrinsically cell-permeable **μMax20** analogs offers a promising route to engineer cell permeable TF mimetics for various applications without requiring additional delivery sequences or specialized delivery systems.^36^

### Functional Assays with μMax20 Analogs Confirm Inhibition of Myc Activity and Cancer Cell Proliferation

Having established that **μMax20** and its stapled analogs efficiently penetrate cells and localize into the nucleus, we assessed their ability to inhibit Myc activity. For these experiments, we employed a luciferase-based Myc reporter assay in HeLa cells that were cotransfected with a luciferase construct containing: (1) firefly luciferase under the control of Myc-responsive E-box elements and (2) constitutively expressed renilla luciferase for normalization (SI section 14). Cells were treated with low micromolar concentrations of **WT Max, μMax20, 2S**_**p**_ **-μMax20, 2S**_**b**_ **-μMax20**, or the dominant Myc inhibitor **Omomyc** as a positive control. **WT Max** induced only a modest reduction in luciferase expression, with significant effects observed only at 4 μM (Figure 7D). In contrast, all μMax-based analogs significantly suppressed Myc-driven transcription at the tested concentrations, with the stapled **μMax20** analogs showing potent inhibition even at submicromolar doses (e.g., 0.25 μM) and outperforming **Omomyc** (Figures 7D and S42). Importantly, the transcriptional inhibition of Myc by **μMax20** analogs aligns well with their cellular uptake observed by confocal microscopy. Specifically, the TAMRA-labeled versions of **WT Max**, **μMax20**, and the stapled analogs required 4 μM, 1 μM, and 0.25 μM, respectively, to achieve robust intracellular fluorescence (Figure 7B,C), demonstrating that transcriptional activity is linked to intracellular accumulation. These results confirm that **μMax20** analogs are not only cell-permeable but also retain the capacity to interfere with Myc-driven transcriptional function in live cancer cells. The enhanced inhibition by the stapled analogs compared to the linear counterparts highlights the advantages of double-stapling, likely driven by enhanced cell permeability observed in the imaging experiments.

Finally, we assessed the impact of synthetic Max analogs on cancer cell proliferation. HeLa cells were treated with increasing concentrations (1, 4, and 16 μM) of every analog for 72 h and cell viabilities were quantified using resazurin-based assays (SI section 15). None of the analogs induced significant inhibition at 1 μM or 4 μM (Figure 7E); however, the stapled proteins **2S**_**p**_ **-μMax20** and **2S**_**b**_ **-μMax20** exhibited a significant reduction in cell viability at 16 μM, with **2S**_**b**_ **-μMax20** outperforming **2S**_**p**_ **-μMax20** (cell viabilities of 16% and 60%, respectively, Figure 7E). Altogether, these results corroborate the functional activity of the modified **μMax20** analogs as intracellular inhibitors of Myc-driven oncogene expression, showing a consistent trend of enhanced cell penetration (Figure 7B,C), nuclear localization (Figures 7B and S40), transcriptional repression (Figures 7D and S42), and reduced cancer cell proliferation (Figure 7E). The construct **2S**_**b**_ **-μMax20** displays the most potent activity and represents a promising prototype for abiotic TF mimetic in Myc-dependent cancer models.

## Conclusion

The design of homogeneous proteins with novel reactivity represents a cutting-edge frontier at the interface of chemistry and biology.^37^ Creating advanced protein analogs requires versatile strategies to edit specific residues in a precise and controlled manner;^38^ however, the incorporation of novel modifications into native protein sequences remains challenging by conventional biological methods.^39^ Here, we integrated structure-based design, peptide synthesis, and chemoselective ligation to develop novel abiotic TFs with refined functional activity. We chemically synthesized a library of abiotic TF analogs derived from Max TF. Importantly, systematic DNA-binding and cellular studies led to the discovery of **μMax20**, an analog bearing two mutations (e.g., Lys31 and Lys57 to hArg), which exhibited potent DNA-binding to the core E-box site. Although some TFs have been previously described as cell permeable,^40^ likely due to their basic DNA-binding domains, the designed **μMax20** analogs exhibited exceptional cellular internalization at low concentrations and outcompeted natural TFs in functional assays.^41^ Our results indicate that the internalization of stapled **μMax20** analogs might involve both endocytosis and direct translocation, yet further mechanistic analysis will be required in future studies to fully understand their internalization pathways.^42^ Moreover, we found that the stapled advanced analog **2S**_**b**_ **-μMax20** is amenable to cellular delivery at submicromolar concentrations in HeLa cells. Remarkably, this lead analog markedly suppressed Myc-driven transcription and inhibited the proliferation of Myc-dependent cancer cells. In summary, this work demonstrates that site-specific incorporation of noncanonical modifications can reveal novel protein reactivities and accelerate the design of advanced bioactive analogs for therapeutic applications.^43^

## Supplementary Material









## Notes

Synthesis and methods, biochemical and biophysical characterization, and cellular studies of the synthesized compounds (PDF)
Movie S1 (MP4)
Raw and normalized data (XLSX)
8-mer enrichment (XLSX)
