# Supplementary material for: Chemical Engineering of Transcription Factors Uncovered Cell-Permeable μMax Modulators
Source: J Am Chem Soc. 2025 Oct 14;147(46):42647–58. doi: 10.1021/jacs.5c13964 (PMC12636033; doi:10.1021/jacs.5c13964)
Supplement: Supplementary file 1 [file ja5c13964_si_001.pdf]

Supporting Information

Chemical Engineering of Transcription Factors Uncovered Cell-Permeable µMax Modulators

Omer Harel,[1+] Ferran Nadal-Bufi,[2+] Raj V. Nithun,[1] Yumi Minyi Yao,[3] Ariel Afek,[3] Marc Vendrell,[2*] and Muhammad Jbara[1*]

[1]School of Chemistry, Raymond and Beverly Sackler Faculty of Exact Sciences, Tel Aviv University, Tel Aviv, 69978 Israel

[2]Centre for Inflammation Research and IRR Chemistry Hub, Institute for Regeneration and Repair, The University of Edinburgh, EH16 4UU Edinburgh, U.K.

[3]Department of Chemical and Structural Biology, Weizmann Institute of Science, Rehovot 7610001, Israel

*Correspondence to: marc.vendrell@ed.ac.uk, jbaram@tauex.tau.ac.il

Table of Contents

1. EXPERIMENTAL 4
1.1 MATERIALS 4
1.2 LCMS ANALYSIS 5
1.3 PREPARATIVE RP-HPLC PURIFICATION 6
2. DNA AND PROTEIN SEQUENCES 7
3. PREPARATION OF 2-CHLOROTRITYL-HYDRAZINO-RESIN 9
4. CHEMICAL SYNTHESIS OF µMAX ANALOGS SEGMENTS 10
4.1 SYNTHESIS OF SEGMENTS 1 & 4-18 MAX(13-51)-$NHNH_2$ 10
4.2 SYNTHESIS OF SEGMENTS 2, 3, 23 & 24 MAX(13-51)-$NHNH_2$ 13
4.3 SYNTHESIS OF SEGMENT 26 MAX(13-35)-$NHNH_2$ 15
4.4 SYNTHESIS OF SEGMENT 32 MAX(13-51)-$NHNH_2$ 17
4.5 SYNTHESIS OF SEGMENT 19 CYS-MAX(53-93) 18
4.6 SYNTHESIS OF SEGMENTS 20 CYS-MAX(53-93)-LYS(TAMRA) & 25 CYS-MAXLYS57HARG(53-93)-LYS(TAMRA) 20
4.7 SYNTHESIS OF SEGMENTS 21 CYS-MAXLYS57ARG(53-93) & 22 CYS-MAXLYS57HARG(53-93) 22
4.8 SYNTHESIS OF SEGMENTS 27 CYS-MAX(37-93)-LYS & 28 CYS-MAX(37-93)-LYS(TAMRA) 24
4.9 SYNTHESIS OF SEGMENTS 33 CYS-MAX(53-93)-LYS & 34 CYS-MAX(53-93)-LYS(TAMRA) 26
5. CHEMICAL SYNTHESIS OF µMAX VARIANTS 28
5.1 SYNTHESIS OF µMAX VARIANTS VIA ONE-POT NATIVE CHEMICAL LIGATION AND DESULFURIZATION 28
5.2 SYNTHESIS OF TAMRA-LABELED µMAX VARIANTS VIA ONE-POT NATIVE CHEMICAL LIGATION AND DESULFURIZATION 33
5.3 CHEMICAL SYNTHESIS OF STAPLED µMAX $2S_P$-µMAX20 36
5.3.1 Native chemical ligation of $2S_p$-µMax20 segments 36
5.3.2 Stapling of segment 29 38
5.3.3 Decaging of segment 30 40
5.3.4 Stapling of segment 31 42
5.3.5 Synthesis of T-$2S_p$-µMax20 43
5.4 CHEMICAL SYNTHESIS OF STAPLED µMAX $2S_B$-µMAX20 44
5.4.1 Stapling of segment 32 44
5.4.2 One-pot stapling and decaging of segment 33 46
5.4.3 Synthesis of $2S_b$-µMax20 via one-pot native chemical ligation and desulfurizaiton 48
5.4.4 Synthesis of T-$2S_b$-µMax20 51
5.5 SYNTHESIS OF T-NATIVE MAX 51
6. CHEMICAL SYNTHESIS OF OMOMYC 52
6.1 SYNTHESIS OF OMOMYC'S SEGMENTS 52
6.1.1 Synthesis of Segment Omo1 Omomyc(1-51)-$NHNH_2$ 52
6.1.2 Synthesis of segment Omo2 Cys-Omomyc(53-92) 53
6.2 SYNTHESIS OF OMOMYC VIA ONE-POT NATIVE CHEMICAL LIGATION AND DESULFURIZATION FOLLOWED BY CYS DECAGING 55
7. DNA-BINDING ANALYSIS AND ELECTROPHORETIC MOBILITY-SHIFT ASSAY (EMSA) 58
7.1 SINGLE-POINT µMAX ANALOGS EMSA 58
7.2 MULTI-POINT µMAX ANALOGS EMSA 60

7.3 STAPLED µMAX ANALOGS EMSA 61
8. CIRCULAR DICHROISM (CD) ANALYSIS 63
9. PROTEIN BINDING MICROARRAY ANALYSES 63
10. OCTET BIOLAYER INTERFEROMETRY BINDING ASSAY (BLI) 66
11. PROTEOLYTIC STABILITY ASSAYS 67
12. CELL PERMEABILITY AND NUCLEAR LOCALIZATION ASSAYS 68
13. CELLULAR UPTAKE MECHANISM ANALYSIS 72
14. MYC REPORTER GENE ASSAYS 73
15. CELL VIABILITY ASSAYS 74
16. REFERENCES 75

1. Experimental

1.1 Materials

Fmoc-L-Phe-OH, Fmoc-L-Asn(Trt)-OH, Fmoc-L-Gln(Trt)-OH, Fmoc-L-Arg(Pbf)-OH, Fmoc-L-Tyr(tBu)-OH, Fmoc-L-Glu(OtBu)-OH, Fmoc-L-Ala-OH, Fmoc-L-Leu-OH, Fmoc-L-His(Trt)-OH, Fmoc-L-Asp(OtBu)-OH, Fmoc-L-Pro-OH, Fmoc-L-Cys(Trt)-OH, Fmoc-L-Lys(Boc)-OH, Fmoc-L-Lys(alloc)-OH, Fmoc-L-Ile-OH, Fmoc-L-Ser(tBu)-OH, Fmoc-Gly-OH, Fmoc-L-Nle-OH, Fmoc-L-Ser(PO(OBzl)OH)-OH, Boc-L-Cys(Trt)-OH, Boc-L-Cys(Acm)-OH, Fmoc-L-Val-OH, Fmoc-L-Thr(tBu)-OH, Fmoc-Aib-OH, Palladium (II) chloride, Guanidine hydrochloride, Sodium phosphate dibasic, Magnesium chloride, Sodium chloride, Calcium chloride, Potassium chloride, Sodium nitrite, Tris(hydroxymethyl)aminomethane (Tris), L-Glutathione reduced (GSH), Thionyl chloride, 1,3-Bis(bromomethyl)benzene, 2,6-Bis(bromomethyl)pyridine, 5-Carboxytetramethylrhodamine (TAMRA), DL-Dithiothreitol (DTT), Hydrazine hydrate 50%, TWEEN® 20, and Bovine serum albumin were purchased from Sigma-Aldrich. Fmoc-L-homoArg(Pbf)-OH, Fmoc-L-Aad(OtBu)-OH, Fmoc-L-Ala(4-thiazoyl)-OH, and Fmoc-L-Ala(4'-pyridyl)-OH were purchased from Chem-Impex International. Fmoc-L-Cit-OH, Fmoc-L-Cys(Acm)-OH, Tris(2-carboxyethyl)phosphine hydrochloride (TCEP), 4-Mercaptophenylacetic acid (MPAA), and Triisopropylsilane (TIS, 98%) were purchased from S. L. Moran. MES hydrate (99%) was purchased from Holland Moran. Fmoc-L-Cav(Boc)-OH was purchased from Iris-Biotech. Fmoc-L-Gla(OtBu)$_2$-OH and Amicon Ultra-2 Centrifugal Filter Unit with Ultracel-3 membrane were purchased from Mercury Scientific & Industrial Products Ltd. 2,2'-Azobis[2-(2-imidazolin-2-yl)propane] Dihydrochloride (VA-044) was purchased from TCI. Fmoc-L-His(Boc)-OH, HO-TCP(Cl)-ProTide resin 0.3 mmol/g, and Rink Amide ProTide (LL) resin 0.18 mmol/g were purchased from CEM. 1-[Bis(dimethylamino)methylene]-1*H*-1,2,3-triazolo[4,5-*b*]pyridinium 3-oxid hexafluorophosphate (HATU), (2-(1H-benzotriazol-1-yl)-1,1,3,3-tetramethyluronium hexafluorophosphate (HBTU), Hydroxybenzotriazole (HOBt) hydrate were purchased from Luxembourg Bio Technologies Ltd. Oligonucleotides were purchased from Integrated DNA Technologies (IDT, Coralville, IA). Diethyl ether (Et_2O, 99.5% stabilized, AR grade), Dichloromethane (CH_2Cl_2, ≥99.5% stabilized with 50 ppm Amylene), N,N-dimethylformamide (DMF, peptide synthesis grade), Acetonitrile (ACN,

LC/MS grade and HPLC grade), Trifluoroacetic acid (TFA, ≥99% ReagentPlus®), Diisopropylethylamine (DIEA, ≥99% ReagentPlus®), Piperidine (≥99% ReagentPlus®), Formic acid (F.A., 98-100% for LC/MS), and Dimethyl sulfoxide (DMSO, ≥99.5% ReagentPlus®) were purchased from Bio-Lab ltd. Glycerol (>99%), TBE Running buffer (5X), Ethidium Bromide DNA Gel Stain, SureCast Acrylamide Solution 40%, SureCast TEMED, and SureCast APS were purchased from Rhenium. Biosensor Strepavidin (FA) was purchased from Sartorius. Water for all reactions carried out on proteins and for reverse-phase purification was obtained via filtration of deionized water through a MilliporeSigma™ Milli-Q™ Ultrapure Water System. All chemicals obtained from the supplier were used as received without further purification.

1.2 LCMS analysis

LCMS chromatograms were acquired using Thermo Scientific Vanquish HPLC and Thermo Scientific ISQ EM Mass spectrometer. Mobile phases used were A (0.05% F.A. in water) and B (0.05% F.A. in ACN). UV spectrum was acquired at 214 nm.

Method A: bioZen™ 2.6 µm-C4 Widepore LC column (150 x 2.1 mm); LC conditions: 5% B from 0–1.0 min, then a linear gradient from 5% to 50% B from 1.0–11.0 min (i.e. 4.5% per min), 0.3 mL/min flow rate.

Method B: bioZen™ 2.6 µm-C4 Widepore LC column (150 x 2.1 mm); LC conditions: 1% B from 0-1.0 min, then a linear gradient from 1% to 30% B from 1.0-11.0 min (i.e. 2.9% per min), 0.3 mL/min flow rate.

1.3 Preparative RP-HPLC purification

Preparative RP-HPLC was performed using Thermo Scientific DIONEX UltiMate 3000 Variable Wavelength Detector, equipped with a column of choice. Mobile phases used for LC analysis were A (0.05% TFA in water) and B (0.05% TFA in ACN). The following LC methods were used:

Method A: Jupiter® 5 µm C18 300 Å LC column (250 x 10 mm), LC conditions: 5% B from 0–5 min, linear gradient from 5% to 60% B from 5-60 min (i.e. 1% per min) 4 mL/min flow rate at 30 °C.

Method B: Jupiter® 5 µm C18 300 Å LC column (250 x 10 mm), LC conditions: 1% B from 0–5 min, linear gradient from 1% to 40% B from 5-44 min (i.e. 1% per min) 4 mL/min flow rate at 30 °C.

Method C: Jupiter® 5 µm C18 300 Å LC column (250 x 10 mm), LC conditions: 5% B from 0–5 min, linear gradient from 5% to 25% B from 5-10 min (i.e. 4% per min), then a linear gradient from 25%-60% from 10-45 min (i.e. 1% per min) 4 mL/min flow rate at 30 °C.

Method D: XBridge BEH 5µm C4 300 Å LC column (250 x 10 mm), LC conditions: 5% B from 0–5 min, linear gradient from 5% to 60% B from 5-60 min (i.e. 1% per min) 4 mL/min flow rate at 30 °C.

Method E: XBridge BEH 5µm C4 300 Å LC column (250 x 10 mm), LC conditions: 5% B from 0–5 min, then linear gradient from 5% to 20% B from 5-10 min (i.e. 3% per min), then a linear gradient from 20%-60% from 10-50 min (i.e. 1% per min) 4 mL/min flow rate at 30 °C.

2. DNA and protein sequences

Max(13-93) original sequence:

ADKRAHHN^{20}ALERKRRDHI^{30}KDSFHSLRDS^{40}VPSLQGEKAS^{50}RAQILDKATE^{60}YIQYM RRKNH^{70}THQQDIDDLK^{80}RQNALLEQQV^{90}RAL

The Met residue at position 65 was mutated with the homologous norleucine (Nle) residue to avoid Met oxidation.
Underscored are residues that were mutated to canonical/non-canonical counterparts.

Mutations in the sequences of peptide segments 1-28 and 32-34:

Max(13-51)-$NHNH_2$ segments:

1: No mutations; **2:** Lys31Arg; **3:** Lys31hArg; **4:** Arg27hArg; **5:** Arg27Cit; **6:** Arg27Cav; **7:** Arg24hArg; **8:** Glu23Aad; **9:** Glu23Gla; **10:** Glu23pSer; **11:** Ala21Aib; **12:** Asn20Gln; **13:** Asn20Cit; **14:** His19Pyr; **15:** His19Tha; **16:** His19Tyr; **17:** Ala17Aib; **18:** Arg16hArg **23:** Lys31hArg, Ala21Aib, His19Tha; **24:** Lys31hArg, Glu23Aad, Ala21Aib, His19Tha; **32:** Lys31hArg, Ala17Cys, Ala13Cys. (Table 1)

Cys-Max(53-93) segments:

19: No mutations; **20:** Lys addition in C-term; **21:** Lys57Arg; **22:** Lys57hArg; **25:** Lys addition in C-term, Lys57hArg; **33-34**: Lys addition in C-term, Ala92Cys, Gln88Cys, Lys57hArg. (Table 1)

Max(13-35)-$NHNH_2$ segment:

26: Asp32Cys, Lys31hArg. (Table 1)

Cys-Max(37-93) segments:

27-28: Lys addition in C-term, Leu85Cys, Arg81Cys, Lys57hArg. (Table 1)

Mutations in the sequences of different µMax analogs:

Single-point mutated Max (µMax):

µMax1: Lys57Arg; **µMax2:** Lys57hArg; **µMax3:** Lys31Arg; **µMax4:** Lys31hArg; **µMax5:** Arg27hArg; **µMax6:** Arg27Cit; **µMax7:** Arg27Cav; **µMax8:** Arg24hArg; **µMax9:** Glu23Aad; **µMax10:** Glu23Gla; **µMax11:** Glu23pSer; **µMax12:** Ala21Aib; **µMax13:**

Asn20Gln; **μMax14:** Asn20Cit; **μMax15:** His19Pyr; **μMax16:** His19Tha; **μMax17:** His19Tyr; **μMax18:** Ala17Aib; **μMax19:** Arg16hArg. (Table 1)

Multi-point μMax:

μMax20: Lys57hArg, Lys31hArg; **μMax21:** Lys57hArg, Lys31hArg, Ala21Aib, His19Tha; **μMax22:** Lys57hArg, Lys31hArg, Glu23Aad, Ala21Aib, His19Tha. (Table 1)

Stapled μMax:

2S$_p$-μMax20: Lys addition in C-term, Leu85Cys, Arg81Cys, Lys57hArg, Ser36Cys, Asp32Cys, Lys31hArg; **2S$_b$-μMax20:** Lys addition in C-term, Ala92Cys, Gln88Cys, Lys57hArg, Lys31hArg, Ala17Cys, Ala13Cys. (Table 1)

Origin residue	Mutated residues	Origin residue	Mutated residues
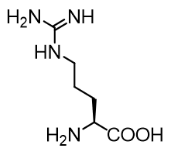 L-Arg	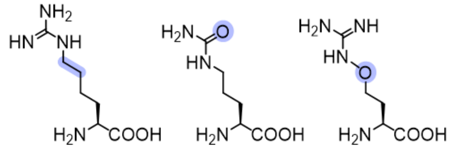 L-hArg L-Cit L-Cav	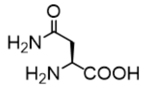 L-Asn	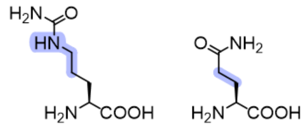 L-Cit L-Gln
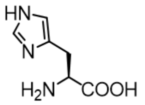 L-His	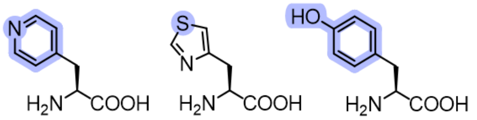 L-Pyr L-Tha L-Tyr	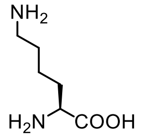 L-Lys	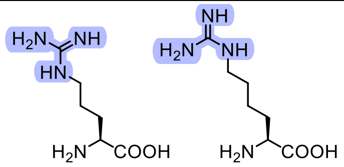 L-Arg L-hArg
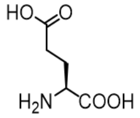 L-Glu	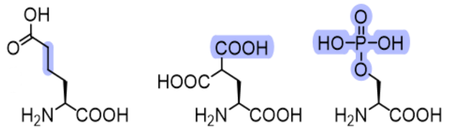 L-Aad L-Gla L-pSer	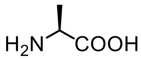 L-Ala	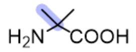 Aib

Table 1: Original amino acid residues and their mutated counterparts.

Omomyc(1-92) sequence

SDTEENVKRR^{10} THNVLERQRR^{20} NELKRSFFAL^{30} RDQIPELENN^{40} EKAPKVVILK^{50} KATAYILSVQ^{60} AETQKLISEI^{70} DLLRKQNEQL^{80} KHKLEQLRNS^{90} CA

E-box DNA probe: (Integrated DNA Technologies)

5’-CCGGCTGA*CACGTG*GTATTAAT-3’

3. Preparation of 2-chlorotrityl-hydrazino-resin

The synthesis was carried out according to the following scheme:

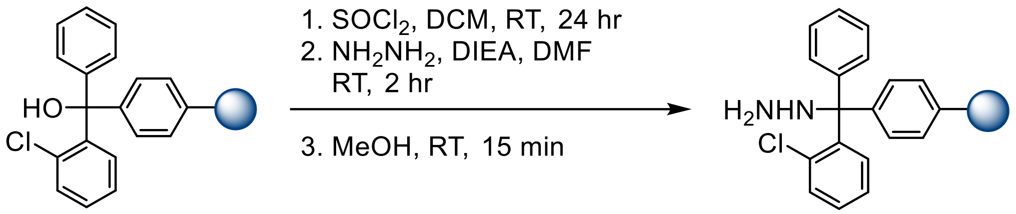

HO-TCP(Cl) ProTide resin
Loading: 0.3 mmol/g

6.6 mL DCM were mixed with $SOCl_2$ (835 µL, 11.5 mmol, 1.5 M) in a septum-capped oven-dried round-bottom-flask that was cooled with N_2. HO-TCP(Cl) resin (loading 0.3 mmol/g, 0.25 mmol scale) was added and the flask was vented 5 times. The mixture was mixed overnight. $SOCl_2$ (335 µL, 4.6 mmol) was added in the following morning and mixed for another 6 hours. The resin was then transferred into a fritted syringe and washed with DCM (8 mL X 6 times). The resin was vacuum dried for 5 minutes and cooled to 0 °C using an ice bath. A solution of DIEA (950 µL, 5.5 mmol, 1.3 M) and Hydrazine hydrate 50% (476 µL, 7.4 mmol, 1.7 M) in DMF (2855 µL) was added to the dried resin in 500 µL portions which resulted in a dark purple suspension. The suspension was stirred at room temperature for 2 hours to receive brown-yellow resin. MeOH (460 µL, 11.4 mmol, 2.4 M) was then added and stirred for an additional 15 min. Finally, the resin was washed with DMF, H_2O, DMF, MeOH, and Et_2O (6 mL X 3 each) and dried under a vacuum.[1]

4. Chemical synthesis of μMax analogs segments

4.1 Synthesis of segments 1 & 4-18 Max(13-51)-NHNH2

The synthesis was carried out according to the following scheme:

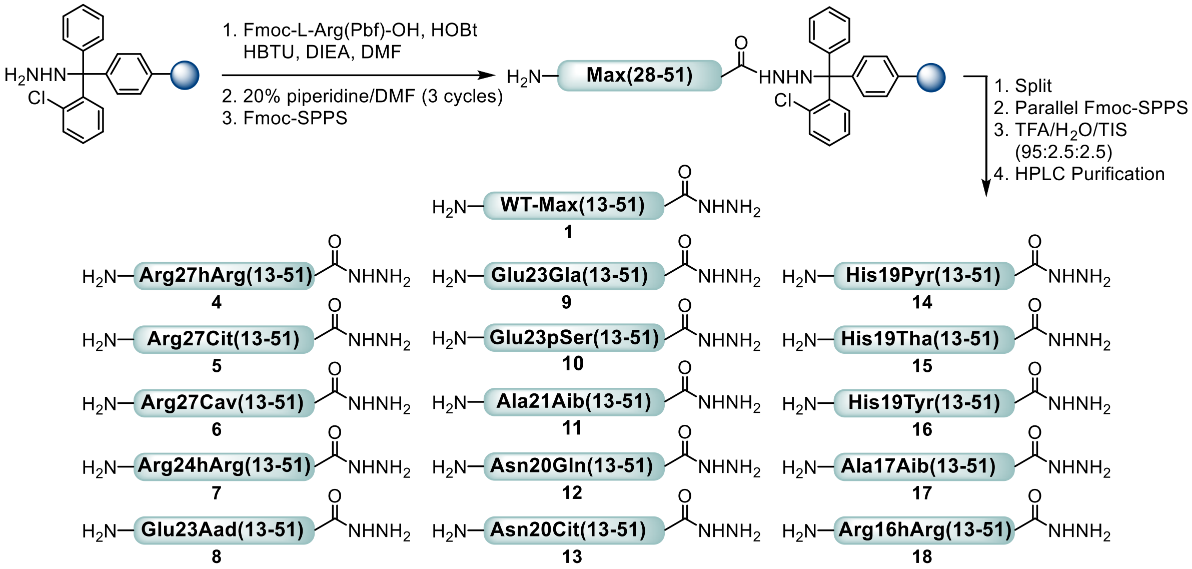

The synthesis of segments **1** and **4-18** was carried out using stepwise Fmoc-SPPS chemistry on hydrazino resin (833.3 mg, loading 0.3 mmol/g, 0.25 mmol scale). The resin was double-coupled with Fmoc-L-Arg(Pbf)-OH (10 equiv., 2.5 mmol, 625 mM), using HBTU (10 equiv., 2.5 mmol, 625 mM), HOBt (10 equiv., 2.5 mmol, 625 mM), and DIEA (10 equiv., 2.5 mmol, 625 mM) in 4 mL DMF for 30 minutes at room temperature (RT). The resin was then transferred to the CSBio automated peptide synthesizer and residues 28-50 were incorporated in a stepwise fashion using Fmoc-L-AA-OH (10 equiv., 2.5 mmol, 166.7 mM), HBTU/HOBt (10 equiv. each, 2.5 mmol, 166.7 mM), and DIEA (20 equiv., 5.0 mmol, 333.3 mM) in 15 mL DMF for 45 minutes at 30 °C per amino acid. Fmoc was deprotected after each coupling step by using three cycles of 20% piperidine and 0.05% F.A. in DMF (15 mL each) for 2, 4, and 2 minutes (Fmoc deprotection step). Then, the resin was split into 10 syringes each containing 0.025 mmol peptidyl bound resin. Each of the syringes was coupled manually with the rest of the sequence parallelly, according to the desired single-point mutated sequences (see Section 2), with Fmoc-L-AA-OH (5 equiv., 0.125 mmol, 125.0 mM), HBTU/HOBt (5 equiv. each, 0.125 mmol, 125.0 mM), and DIEA (5 equiv., 0.125 mmol, 125.0 mM) in 1 mL DMF at RT for 45 minutes followed by an Fmoc deprotection step. Aib was coupled using Fmoc-Aib-OH (10 equiv.,

0.25 mmol, 250.0 mM), HATU (10 equiv., 0.25 mmol, 250.0 mM), and DIEA (10 equiv., 0.25 mmol, 250.0 mM) in 1 mL DMF for 1 hour at RT. The amino acid following Aib was coupled using Fmoc-L-AA-OH (5 equiv., 0.125 mmol, 125.0 mM), HATU (5 equiv., 0.125 mmol, 125.0 mM), and DIEA (5 equiv., 0.125 mmol, 125.0 mM) in 1 mL DMF for 1 hour at RT. Finally, all peptide-bound resins were washed with DMF (5 mL x 3), MeOH (5 mL x 3), and DCM (5 mL x 3) and dried under vacuum. To remove side chain protecting groups and release the peptide chains, a mixture of TFA/H_2O/TIS (95:2.5:2.5, 5 mL for 0.025 mmol scale) was added to each resin and shaken for 4 hours at RT. The resin was removed by filtration and extracted with TFA (2 x 1 mL). To precipitate the peptide, each filtrate was added dropwise to cold diethyl ether (25 mL for 0.025 mmol resin) followed by centrifugation at 4000 rpm for 7 min. Then, the diethyl ether was decanted, followed by the dissolution of the peptide in 50% ACN/water, dilution to 25% ACN/water, and lyophilization to acquire white powders. The process was repeated one more time to obtain all needed hydrazide segments. The dry crude powders were purified by RP-HPLC (Method A described in Section 1.3) affording the products as white powders in the following yields: **1** (19.4 mg, 4.3 μmol, 17% yield), **4** (15.1 mg, 3.3 μmol, 13% yield), **5** (15.2 mg, 3.3 μmol, 13% yield), **6** (7.5 mg, 1.6 μmol, 6% yield), **7** (11.3 mg, 2.5 μmol, 10% yield), **8** (13.5 mg, 3.0 μmol, 12% yield), **9** (10.1 mg, 2.2 μmol, 9% yield), **10** (12.3 mg, 2.7 μmol, 11% yield), **11** (22.7 mg, 5.0 μmol, 20% yield), **12** (25.1 mg, 5.5 μmol, 22% yield), **13** (3.5 mg, 0.8 μmol, 3% yield), **14** (20.8 mg, 4.5 μmol, 18% yield), **15** (12.3 mg, 2.7 μmol, 11% yield), **16** (15.5 mg, 3.4 μmol, 13% yield), **17** (21.5 mg, 4.7 μmol, 19% yield), **18** (13.5 mg, 3.0 μmol, 12% yield).

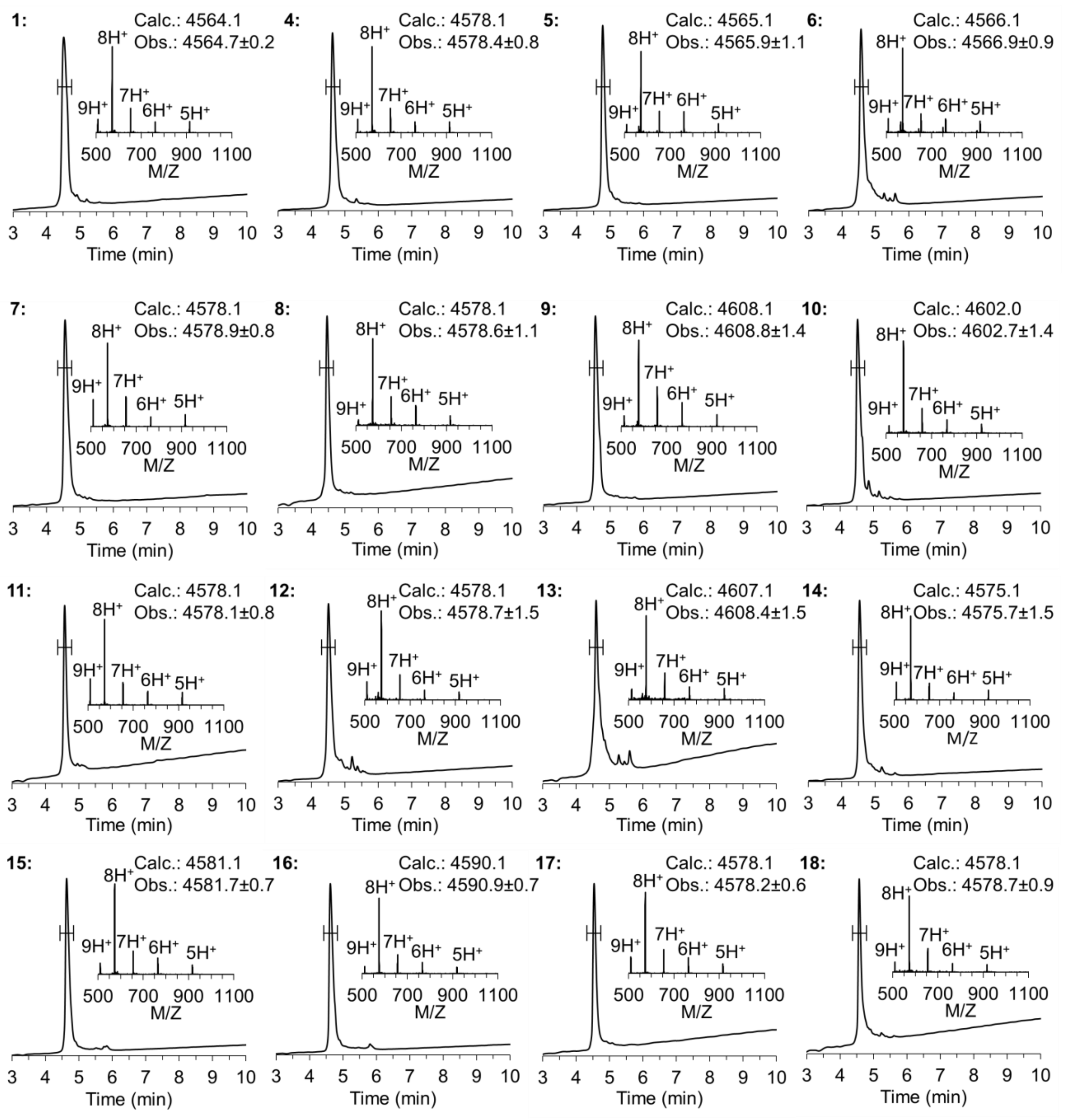

Figure S1. LCMS analysis of segments 1 and 4-18. LC of the UV absorbance at 214 nm and mass-to-charge (M/Z) spectrum. LCMS analysis was carried out with Method A (see section 1.2). M/Z data were acquired over the marked regions in the chromatograms. Calculated and observed masses are reported in Da (average isotopes).

4.2 Synthesis of segments 2, 3, 23 & 24 Max(13-51)-NHNH$_2$

The synthesis was carried out according to the following scheme:

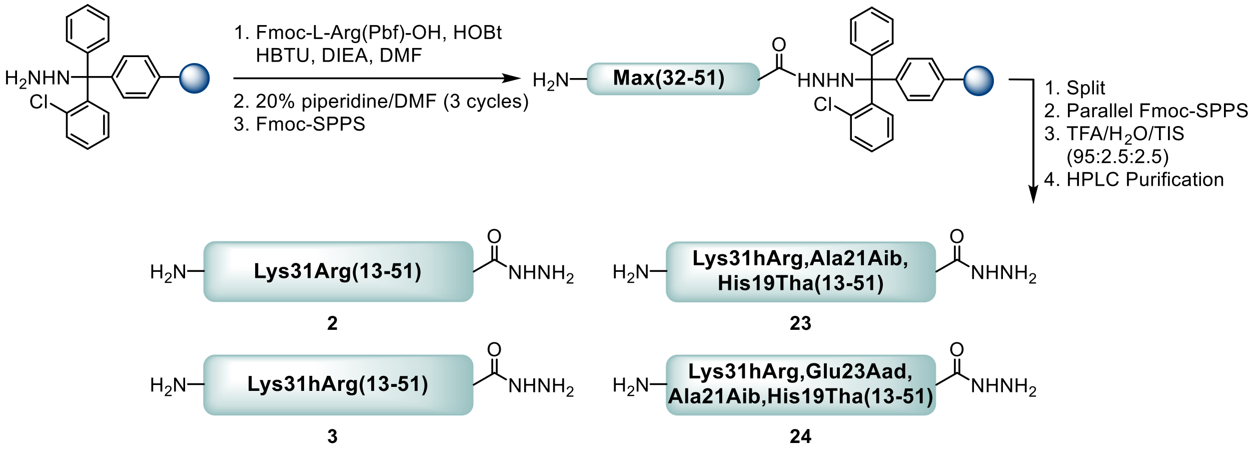

The synthesis of segments **2, 3, 23,** and **24** was carried out using stepwise Fmoc-SPPS chemistry on hydrazino resin (333.3 mg, loading 0.3 mmol/g, 0.1 mmol scale). The resin was double-coupled with Fmoc-L-Arg(Pbf)-OH (10 equiv., 1.0 mmol, 500 mM), using HBTU/HOBt (10 equiv. each, 1.0 mmol, 500 mM), and DIEA (10 equiv., 1.0 mmol, 500 mM) in 2 mL DMF for 30 minutes each. The resin was then transferred to the CSBio automated peptide synthesizer and residues 32-50 were incorporated in a stepwise fashion using Fmoc-L-AA-OH (10 equiv., 1.0 mmol, 66.7 mM), HBTU/HOBt (10 equiv. each, 1.0 mmol, 66.7 mM) and DIEA (20 equiv., 2.0 mmol, 133.4 mM) in 15 mL DMF for 45 minutes at 30 °C per amino acid, followed by an Fmoc deprotection step. Then, the resin was split into four syringes each containing 0.025 mmol. Fmoc-L-Arg(Pbf)-OH for segment **2** or Fmoc-L-hArg(Pbf)-OH for segments **3**, **23**, and **24** (5 equiv., 0.125 mmol, 125.0 mM) were coupled using HBTU/HOBt (5 equiv. each, 0.125 mmol, 125.0 mM), and DIEA (5 equiv., 0.125 mmol, 125.0 mM) in 1 mL DMF for 45 minutes at RT. After Fmoc deprotection, residues 24-30 were double coupled at RT for 30 minutes each round using Fmoc-L-AA-OH (5 equiv., 0.125 mmol, 125.0 mM), HATU (5 equiv., 0.125 mmol, 125.0 mM), and DIEA (5 equiv., 0.125 mmol, 125.0 mM) in 1 mL DMF. The rest of the sequences were coupled according to the desired mutated sequences (see Section 2) using Fmoc-L-AA-OH (5 equiv., 0.125 mmol, 125.0 mM), HBTU/HOBt (5 equiv. each, 0.125 mmol, 125.0 mM), and DIEA (5 equiv., 0.125 mmol, 125.0 mM) in 1 mL DMF at RT for 45 minutes. Aib was coupled using Fmoc-Aib-OH (10 equiv., 0.25 mmol, 250.0 mM),

HATU (10 equiv., 0.25 mmol, 250.0 mM), and DIEA (10 equiv., 0.25 mmol, 250.0 mM) in 1 mL DMF for 1 hour at RT. The amino acid following Aib was coupled using Fmoc-L-AA-OH (5 equiv., 0.125 mmol, 125.0 mM), HATU (5 equiv., 0.125 mmol, 125.0 mM), and DIEA (5 equiv., 0.125 mmol, 125.0 mM) in 1 mL DMF for 1 hour at RT. Finally, all peptide-bound resins were washed with DMF (5 mL x 3), MeOH (5 mL x 3), and DCM (5 mL x 3) and dried under vacuum. To remove side chain protecting groups and release the peptide chains, a mixture of TFA/H_2O/TIS (95:2.5:2.5, 5 mL for 0.025 mmol scale) was added to each resin and shaken for 4 hours at RT. The resin was removed by filtration and extracted with TFA (2 × 1 mL). To precipitate the peptides, each filtrate was added dropwise to cold diethyl ether (25 mL for 0.025 mmol resin) followed by centrifugation at 4000 rpm for 7 min. Then, the diethyl ether was decanted, followed by the dissolution of each peptide in 50% ACN/water, dilution to 25% ACN/water, and lyophilization to obtain white powders. The dry crude powders were purified by RP-HPLC (Method A described in Section 1.3) affording the products as white powders in the following yields: **2** (23.5 mg, 5.1 μmol, 21% yield), **3** (23.0 mg, 5.0 μmol, 21% yield), **23** (10.5 mg, 2.3 μmol, 10% yield), **24** (10.5 mg, 2.3 μmol, 10% yield).

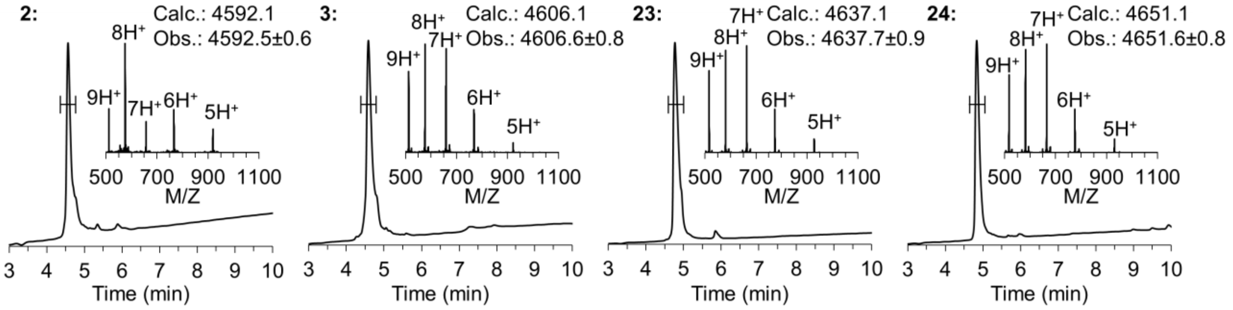

Figure S2. LCMS analysis of segments 2, 3, 23, and 24. LC of the UV absorbance at 214 nm and mass-to-charge (M/Z) spectrum. LCMS analysis was carried out with Method A (see section 1.2). M/Z data were acquired over the marked regions in the chromatograms. Calculated and observed masses are reported in Da (average isotopes).

4.3 Synthesis of segment 26 Max(13-35)-$NHNH_2$

The synthesis was carried out according to the following scheme:

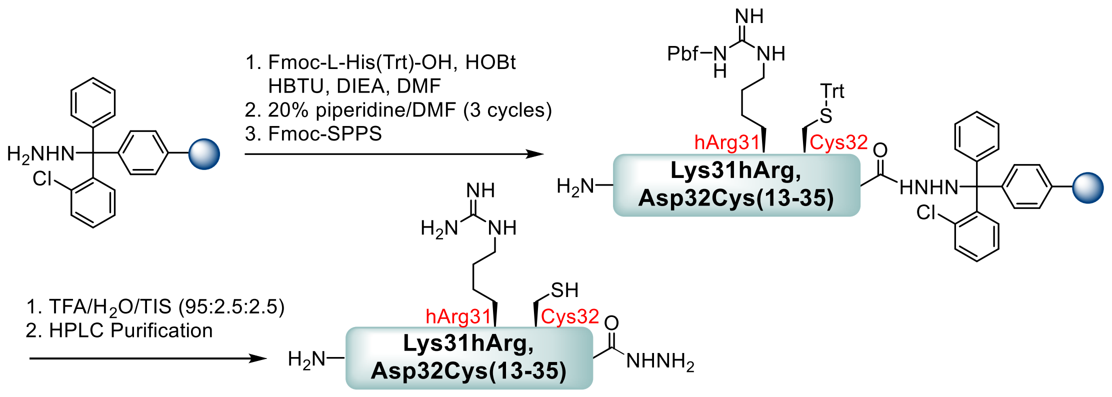

26

The synthesis of segment **26** was carried out using stepwise Fmoc-SPPS chemistry on hydrazino resin (333.3 mg, loading 0.3 mmol/g, 0.1 mmol scale). The resin was double coupled with Fmoc-L-His(Trt)-OH (10 equiv., 1.0 mmol, 500 mM), using HBTU (10 equiv., 1.0 mmol, 500 mM), HOBt (10 equiv., 1.0 mmol, 500 mM), and DIEA (10 equiv., 1.0 mmol, 500 mM) in 2 mL DMF for 30 minutes each. The resin was then transferred to the CSBio automated peptide synthesizer and the rest of the sequence (see Section 2) was coupled using Fmoc-L-AA-OH (10 equiv., 1.0 mmol, 66.7 mM), using HBTU/HOBt (10 equiv. each, 1.0 mmol, 66.7 mM), and DIEA (20 equiv., 2.0 mmol, 133.4 mM) in 15 mL DMF for 45 minutes at 30 °C per amino acid followed by an Fmoc deprotection step. Finally, the peptide-bound resin was washed with DMF (5 mL x 3), MeOH (5 mL x 3), and DCM (5 mL x 3) and dried under vacuum. To remove side chain protecting groups and release the peptide chain, a mixture of TFA/H_2O/TIS (95:2.5:2.5, 5 mL for 0.025 mmol scale) was added to the resin and shaken for 4 hours at RT. The resin was removed by filtration and extracted with TFA (2 × 1 mL). To precipitate the peptide, the filtrate was added dropwise to cold diethyl ether (25 mL for 0.025 mmol resin) followed by centrifugation at 4000 rpm for 7 min. Then, the diethyl ether was decanted, followed by the dissolution of the peptide in 50% ACN/water, dilution to 25% ACN/water, and lyophilization to obtain a white powder. The dry crude powder was purified by RP-HPLC (Method B described in Section 1.3) affording the product as a white powder in the following yield: (12.7 mg, 4.4 μmol, 9% yield based on 0.05 mmol).

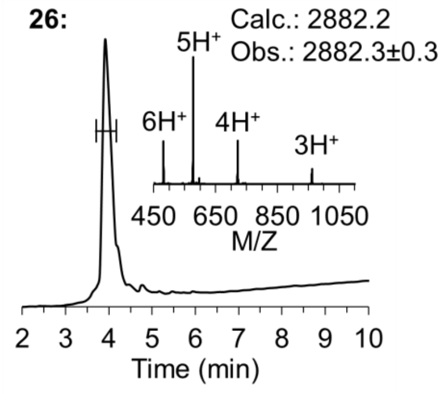

Figure S3. LCMS analysis of segments 26. LC of the UV absorbance at 214 nm and mass-to-charge (M/Z) spectrum. LCMS analysis was carried out with Method B (see section 1.2). M/Z data was acquired over the marked region in the chromatogram. Calculated and observed masses are reported in Da (average isotopes).

4.4 Synthesis of segment 32 Max(13-51)-$NHNH_2$

The synthesis was carried out according to the following scheme:

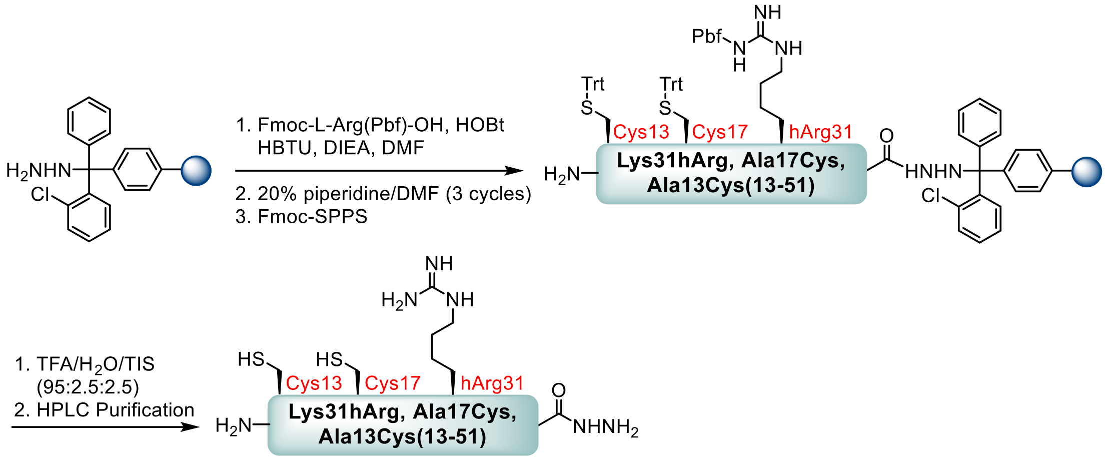

32

The synthesis of segment **32** was carried out in a similar manner to segment **3**. The segment was synthesized at a 0.1 mmol scale, incorporating Fmoc-L-Cys(Trt)-OH instead of Fmoc-L-Ala-OH at positions 13 and 17. The segment was cleaved, dissolved, and dried in a similar manner to segment **3** to obtain a white powder. The dry crude powder was purified by RP-HPLC (Method A described in Section 1.3) affording the product as a white powder in the following yield: (52.0 mg, 11.1 µmol, 11% yield).

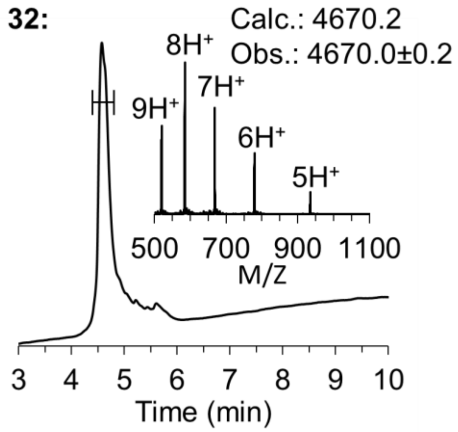

Figure S4. LCMS analysis of segments 32. LC of the UV absorbance at 214 nm and mass-to-charge (M/Z) spectrum. LCMS analysis was carried out with Method A (see section 1.2). M/Z data was acquired over the marked region in the chromatogram. Calculated and observed masses are reported in Da (average isotopes).

4.5 Synthesis of segment 19 Cys-Max(53-93)

The synthesis was carried out according to the following scheme:

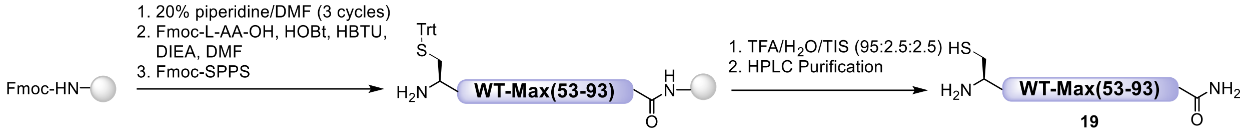

The synthesis of segment **19** was carried out using stepwise Fmoc-SPPS chemistry on Rink amide Protide^{TM} resin (1111.1 mg, loading 0.18 mmol/g, 0.2 mmol scale). The resin was pre-swelled in DMF for 30 min and then transferred to the CSBio automated peptide synthesizer starting with an Fmoc deprotection step followed by coupling of the full sequence using Fmoc-L-AA-OH (10 equiv., 2.0 mmol, 133.3 mM), HBTU/HOBt (10 equiv. each, 2.0 mmol, 133.3 mM), and DIEA (20 equiv., 4.0 mmol, 266.6 mM) in 15 mL DMF for 45 minutes at 30 °C per amino acid, followed by an Fmoc deprotection step. The peptide-bound resin was washed with DMF (5 mL x 3), MeOH (5 mL x 3), and DCM (5 mL x 3) and dried under vacuum. To remove side chain protecting groups and release the peptide chains, a mixture of TFA/H_2O/TIS (95:2.5:2.5, 10 mL for 0.025 mmol scale) was added to each resin and shaken for 4 hours at RT. The resin was removed by filtration and extracted with TFA (2 × 1 mL). To precipitate the peptide, the filtrate was added dropwise to cold diethyl ether (35 mL for 0.025 mmol resin) followed by centrifugation at 4000 rpm for 7 min. Then, the diethyl ether was decanted, followed by the dissolution of the peptide in 50% ACN/water, dilution to 25% ACN/water, and lyophilization to obtain a white powder. The dry crude powder was purified by RP-HPLC (Method A described in Section 1.3) affording the product as a white powder in the following yield: (35.3 mg, 6.9 µmol, 28% yield based on 0.025 mmol resin).

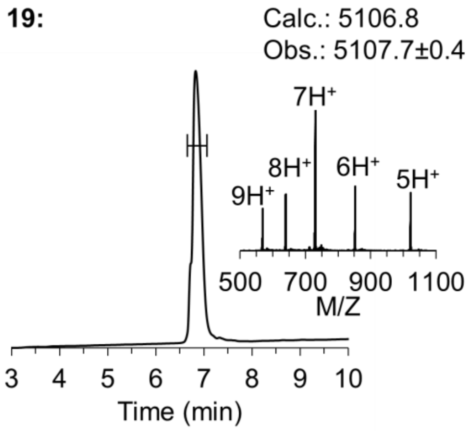

Figure S5. LCMS analysis of segment 19. LC of the UV absorbance at 214 nm and mass-to-charge (M/Z) spectrum. LCMS analysis was carried out with Method A (see section 1.2). M/Z data was acquired over the marked region in the chromatogram. Calculated and observed masses are reported in Da (average isotopes).

4.6 Synthesis of segments 20 Cys-Max(53-93)-Lys(TAMRA) & 25 Cys-MaxLys57hArg(53-93)-Lys(TAMRA)

The synthesis was carried out according to the following scheme:

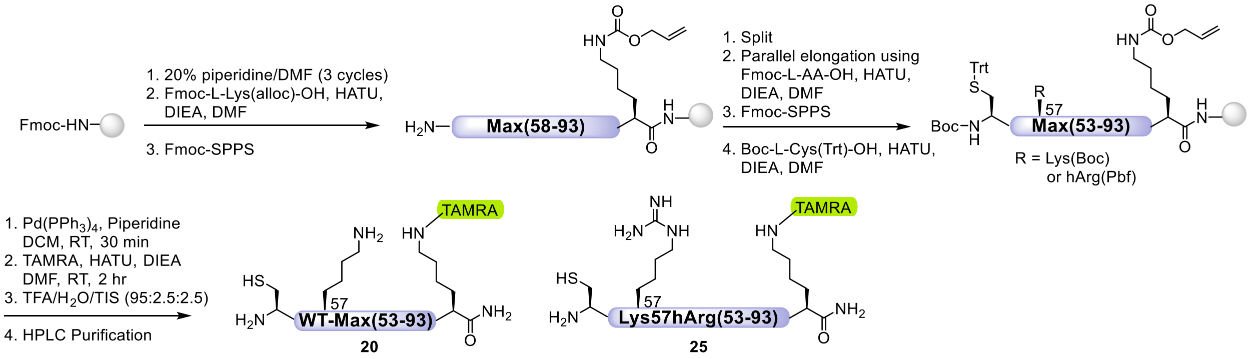

The synthesis of segments **20** and **25** was carried out using stepwise Fmoc-SPPS chemistry on Rink amide Protide^{TM} resin (555.6 mg, loading 0.18 mmol/g, 0.1 mmol scale). The resin was pre-swelled in DMF for 30 min and treated with an Fmoc deprotection step. Then, the deprotected resin was coupled with Fmoc-L-Lys(alloc)-OH (5 equiv., 0.5 mmol, 250 mM) using HATU (5 equiv., 0.5 mmol, 250 mM), and DIEA (10 equiv., 1.0 mmol, 500 mM) in 2 mL DMF for 45 minutes at RT. The resin was then transferred to the CSBio automated peptide synthesizer and residues 58-93 were coupled using Fmoc-L-AA-OH (10 equiv., 1.0 mmol, 66.7 mM), HBTU/HOBt (10 equiv. each, 1.0 mmol, 66.7 mM), and DIEA (20 equiv., 2.0 mmol, 133.4 mM) in 15 mL DMF for 15 minutes at 60 °C per amino acid followed by an Fmoc deprotection step. The resin was then split into two syringes each containing 0.05 mmol that were coupled with Fmoc-L-Lys(boc)-OH for segment **20** or with Fmoc-L-hArg(Pbf)-OH for segment **25**. The coupling of these residues was carried out using Fmoc-L-AA-OH (5 equiv., 0.25 mmol, 125.0 mM), HATU (5 equiv., 0.25 mmol, 125.0 mM), and DIEA (5 equiv., 0.25 mmol, 125 mM) in 2 mL DMF for 45 minutes at RT. Residues 53-56 were coupled in the same conditions to each resin. Finally, Boc-L-Cys(Trt)-OH (10 equiv., 0.5 mmol, 250.0 mM) was coupled to each resin using HATU (10 equiv., 0.5 mmol, 250.0 mM) and DIEA (10 equiv., 0.5 mmol, 250.0 mM) in 2 mL DMF for 1 hour at RT. Each peptide-bound resin was washed with DMF (5 mL x 3), MeOH (5 mL x 3), and DCM (5 mL x 3) and dried under vacuum. Subsequently, to remove the alloc protecting group, a mixture of $Pd(PPh_3)_4$ (1 equiv.), piperidine (640 µL), and DCM (2560 µL) was added to each resin and was shaken for 30 minutes at RT under

the exclusion of light. The resins were washed with DCM (6 mL x 6) and DMF (6 mL x 6). Then, each resin was coupled with TAMRA (2 equiv., 0.1 mmol, 50 mM) using HATU (2 equiv., 0.1 mmol, 50 mM) and DIEA (4 equiv., 0.2 mmol, 100 mM) in 2 mL DMF for 2 hours at RT. Finally, the peptide-bound resins were washed with DMF (5 mL x 3), MeOH (5 mL x 3), and DCM (5 mL x 3) and dried under vacuum. To remove side chain protecting groups and release the peptide chains, a mixture of TFA/H_2O/TIS (95:2.5:2.5, 10 mL for 0.025 mmol scale) was added to each resin and shaken for 4 hours at RT. The resin was removed by filtration and extracted with TFA (2 × 1 mL). To precipitate the peptide, each filtrate was added dropwise to cold diethyl ether (15 mL for 0.025 mmol resin) followed by centrifugation at 4000 rpm for 7 min. Then, the diethyl ether was decanted, followed by the dissolution of the peptide in 50% ACN/water, dilution to 25% ACN/water, and lyophilization to acquire pink powders. The dry crude powders were purified by RP-HPLC (Method C described in Section 1.3) affording the products as pink powders in the following yields: **20** (19.0 mg, 3.4 μmol, 13% yield based on 0.025 mmol resin), **25** (21.2 mg, 3.7 μmol, 15% yield based on 0.025 mmol resin).

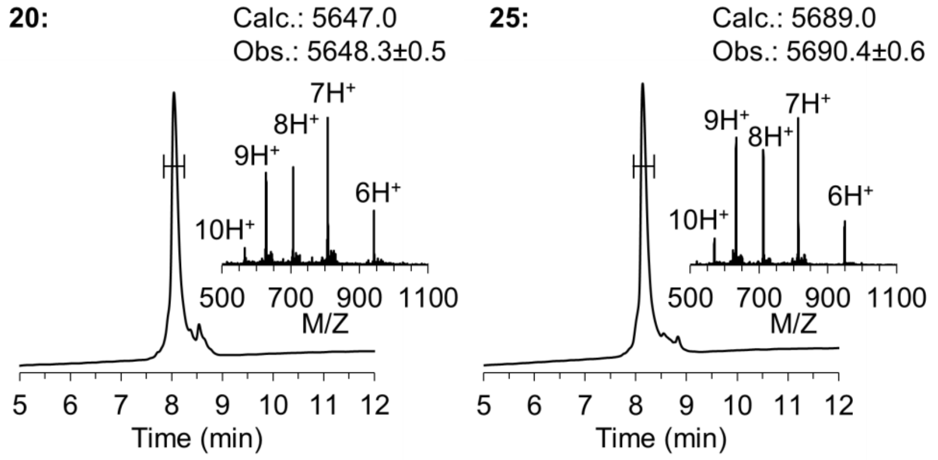

Figure S6. LCMS analysis of segments 20 and 25. LC of the UV absorbance at 214 nm and mass-to-charge (M/Z) spectrum. LCMS analysis was carried out with Method A (see section 1.2). M/Z data were acquired over the marked regions in the chromatograms. Calculated and observed masses are reported in Da (average isotopes).

4.7 Synthesis of segments 21 Cys-MaxLys57Arg(53-93) & 22 Cys-MaxLys57hArg(53-93)

The synthesis was carried out according to the following scheme:

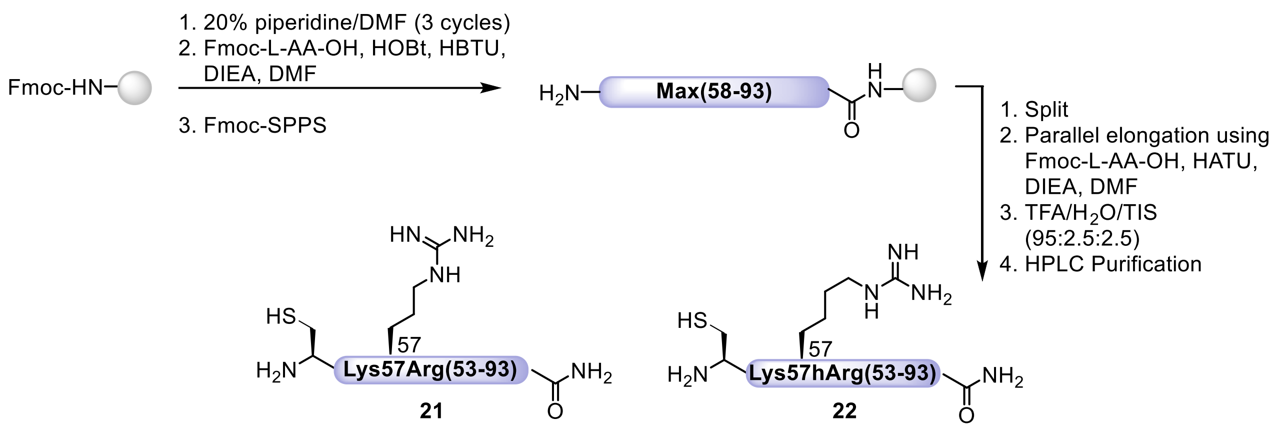

The synthesis of segments **21** and **22** was carried out using stepwise Fmoc-SPPS chemistry on Rink amide Protide™ resin (1111.2 mg, loading 0.18 mmol/g, 0.2 mmol scale). The resin was pre-swelled in DMF for 30 min, loaded onto the CSBio automated peptide synthesizer, and treated with an Fmoc deprotection step. Residues 58-93 were coupled using Fmoc-L-AA-OH (10 equiv., 2.0 mmol, 133.3 mM), HBTU/HOBt (10 equiv. each, 2.0 mmol, 133.3 mM), and DIEA (20 equiv., 4.0 mmol, 266.6 mM) in 15 mL DMF for 15 minutes at 60 °C per amino acid, followed by an Fmoc deprotection step. Then, 0.025 mmol peptide bound resin was transferred into two syringes. Fmoc-L-Arg(Pbf)-OH was coupled for segment **21** and Fmoc-L-hArg(Pbf)-OH was coupled for segment **22** (5 equiv., 0.125 mmol, 125 mM) using HATU (5 equiv., 0.125 mmol, 125 mM), and DIEA (5 equiv., 0.125 mmol, 125 mM) in 1 mL DMF for 45 minutes at RT. The rest of the sequence was coupled in each syringe using Fmoc-L-AA-OH and the same coupling cocktail. Finally, the peptide-bound resins were washed with DMF (5 mL x 3), MeOH (5 mL x 3), and DCM (5 mL x 3) and dried under vacuum. To remove side chain protecting groups and release the peptide chains, a mixture of TFA/H_2O/TIS (95:2.5:2.5, 10 mL for 0.025 mmol scale) was added to each resin and shaken for 4 hours at RT. Each resin was removed by filtration and extracted with TFA (2 × 1 mL). To precipitate the peptides, each filtrate was added dropwise to cold diethyl ether (35 mL for 0.025 mmol resin) followed by centrifugation at 4000 rpm for 7 min. Then, the diethyl ether was decanted, followed by the dissolution of the peptide in 50% ACN/water, dilution to 25% ACN/water, and

lyophilization to obtain white powders. The dry crude powders were purified by RP-HPLC (Method A described in Section 1.3) affording the products as white powders in the following yields: **21** (30.1 mg, 5.8 μmol, 23% yield), **22** (10.3 mg, 2.0 μmol, 8% yield).

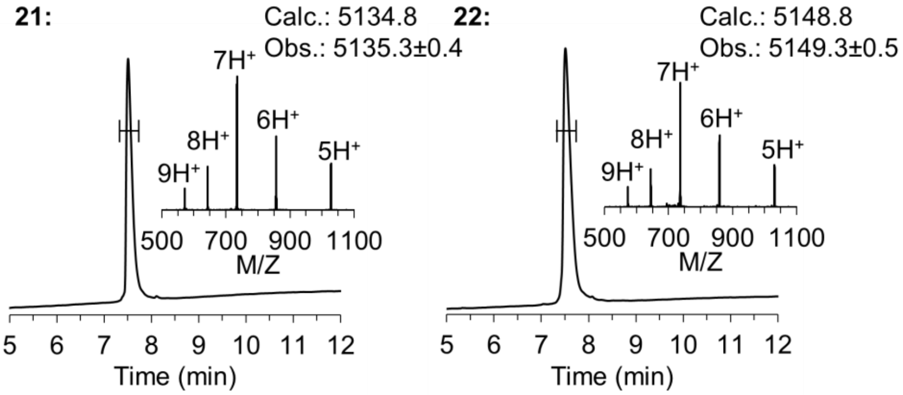

Figure S7. LCMS analysis of segments 21 and 22. LC of the UV absorbance at 214 nm and mass-to-charge (M/Z) spectrum. LCMS analysis was carried out with Method A (see section 1.2). M/Z data were acquired over the marked regions in the chromatograms. Calculated and observed masses are reported in Da (average isotopes).

4.8 Synthesis of segments 27 Cys-Max(37-93)-Lys & 28 Cys-Max(37-93)-Lys(TAMRA)

The synthesis was carried out according to the following scheme:

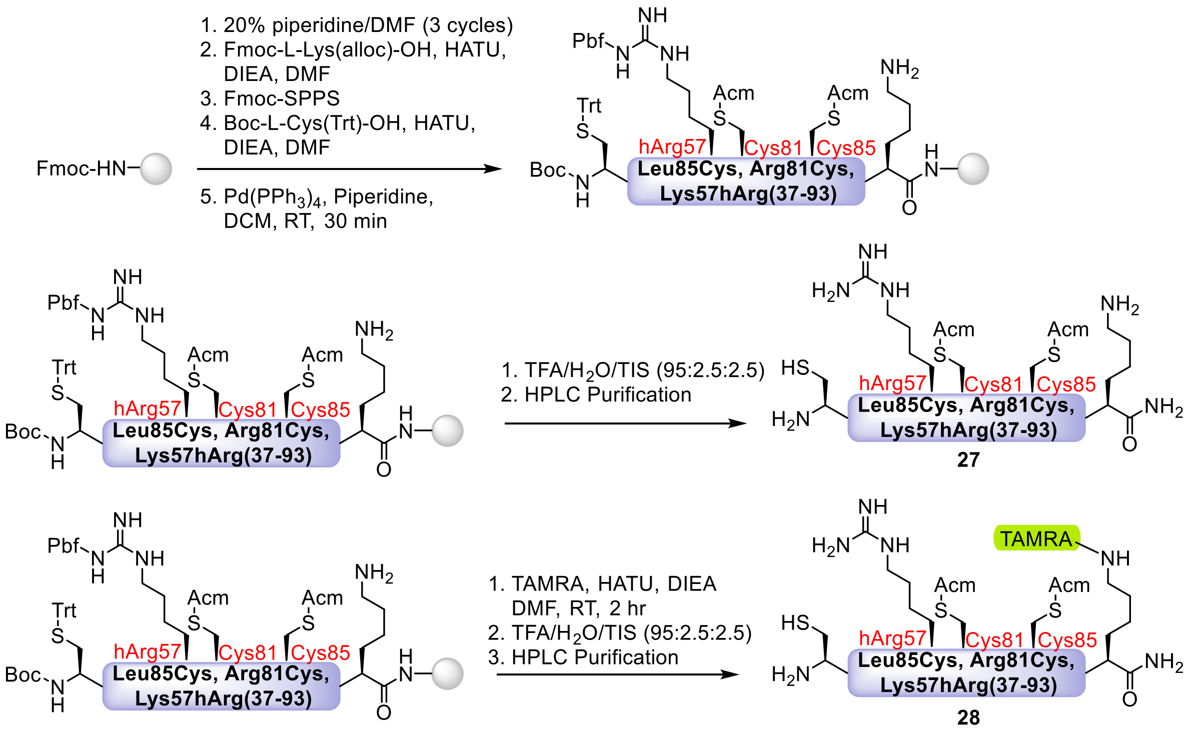

The synthesis of segments **27** and **28** was carried out using stepwise Fmoc-SPPS chemistry on Rink amide Protide^{TM} resin (555.6 mg, loading 0.18 mmol/g, 0.1 mmol scale). The resin was pre-swelled in DMF for 30 min and treated with an Fmoc deprotection step. Then, the deprotected resin was coupled with Fmoc-L-Lys(alloc)-OH (5 equiv., 0.5 mmol, 250 mM) using HATU (5 equiv., 0.5 mmol, 250 mM) and DIEA (10 equiv., 1.0 mmol, 500 mM) in 2 mL DMF for 45 minutes at RT. The resin was then loaded onto the CSBio automated peptide synthesizer and the full sequence was coupled using Fmoc-L-AA-OH (10 equiv., 1.0 mmol, 66.7 mM), HBTU/HOBt (10 equiv. each, 1.0 mmol, 66.7 mM), and DIEA (20 equiv., 2.0 mmol, 133.4 mM) in 15 mL DMF for 15 minutes at 60 °C per amino acid, followed by an Fmoc deprotection step. Boc-L-Cys(Trt)-OH (10 equiv., 1.0 mmol, 500 mM) was then coupled using HATU (10 equiv., 1.0 mmol, 500 mM), and DIEA (10 equiv., 1.0 mmol, 500 mM) in 2 mL DMF for 1 hour at RT. To obtain segment **28**, 0.05 mmol of the peptide bound resin was washed with DMF (5 mL x 3), MeOH (5 mL x 3), and DCM (5 mL x 3) and was subjected to alloc removal using a mixture of $Pd(PPh_3)_4$

(1 equiv.), piperidine (640 µL), and DCM (2560 µL) that was shaken with the resin for 30 minutes at RT under the exclusion of light. The resin was washed with DCM (6 mL x 6) and DMF (6 mL x 6). Then, TAMRA (2 equiv., 0.1 mmol, 50 mM) was coupled using HATU (2 equiv., 0.1 mmol, 50 mM) and DIEA (4 equiv., 0.2 mmol, 100 mM) in 2 mL DMF for 2 hours at RT. Finally, the peptide-bound resin was washed with DMF (5 mL x 3), MeOH (5 mL x 3), and DCM (5 mL x 3) and dried under vacuum. To obtain segment **27**, the same process was repeated with the rest of the resin (0.05 mmol) without the TAMRA coupling step. To remove side chain protecting groups and release the peptide chains, a mixture of TFA/H_2O/TIS (95:2.5:2.5, 10 mL for 0.025 mmol scale) was added to each resin and shaken for 4 hours at RT. The resins were removed by filtration and extracted with TFA (2 × 1 mL). To precipitate the peptides, each filtrate was added dropwise to cold diethyl ether (15 mL for 0.025 mmol resin) followed by centrifugation at 4000 rpm for 7 min. Then, the diethyl ether was decanted, followed by the dissolution of the peptide in 50% ACN/water, dilution to 25% ACN/water, and lyophilization to obtain pink or white powders. The dry crude powders were purified by RP-HPLC (Method C described in Section 1.3) affording the products in the following yields: **27** (17.6 mg, 2.5 µmol, 10% yield based on 0.025 mmol resin), **28** (17.2 mg, 2.3 µmol, 9% yield based on 0.025 mmol resin).

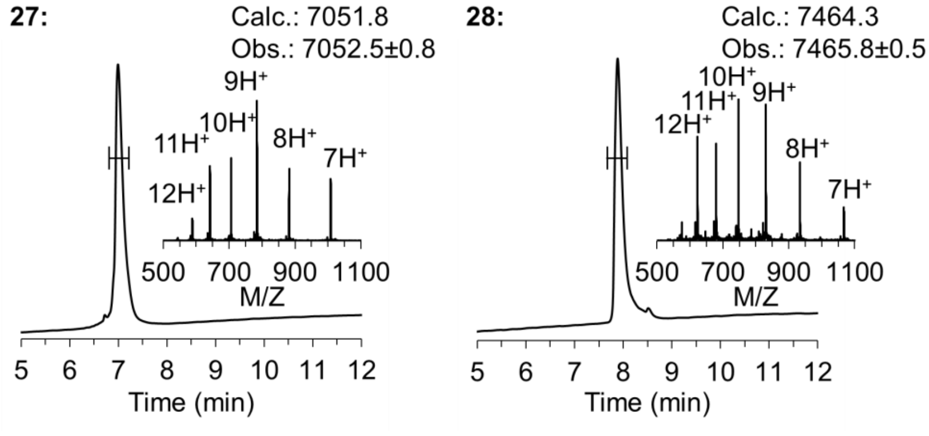

Figure S8. LCMS analysis of segments 27 and 28. LC of the UV absorbance at 214 nm and mass-to-charge (M/Z) spectrum. LCMS analysis was carried out with Method A (see section 1.2). M/Z data were acquired over the marked regions in the chromatograms. Calculated and observed masses are reported in Da (average isotopes).

4.9 Synthesis of segments 33 Cys-Max(53-93)-Lys & 34 Cys-Max(53-93)-Lys(TAMRA)

The synthesis was carried out according to the following scheme:

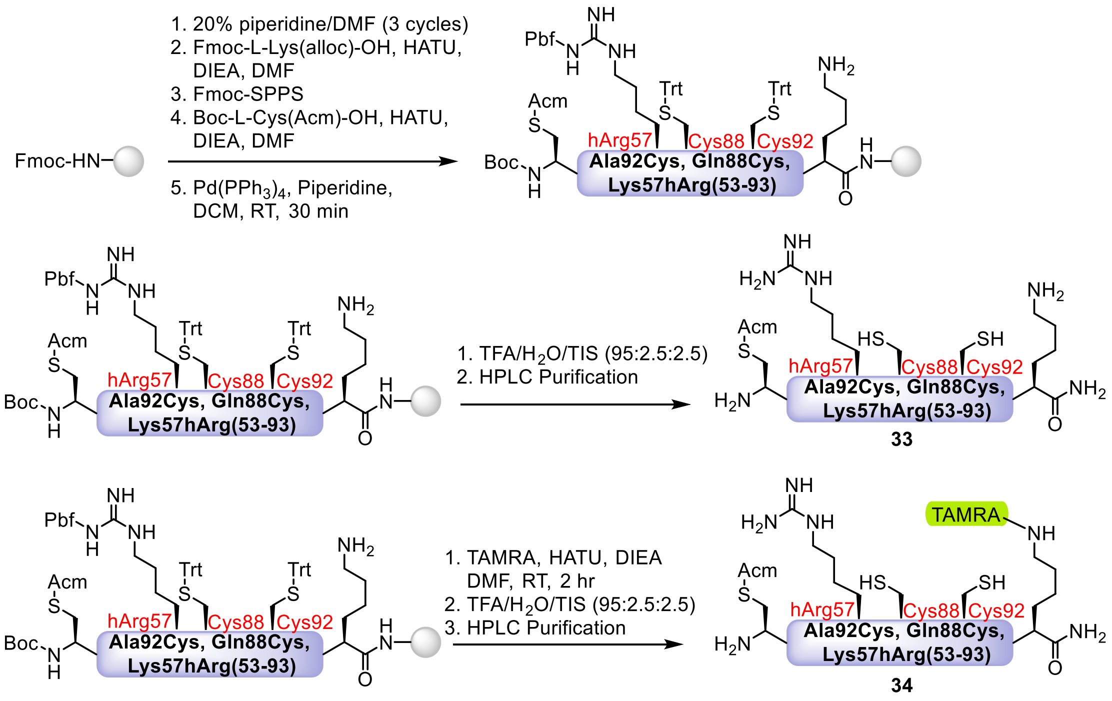

The synthesis of segments **33** and **34** was carried out using stepwise Fmoc-SPPS chemistry on Rink amide Protide™ resin (555.6 mg, loading 0.18 mmol/g, 0.1 mmol scale). The resin was pre-swelled in DMF for 30 min and treated with an Fmoc deprotection step. Then, the deprotected resin was coupled with Fmoc-L-Lys(alloc)-OH (5 equiv., 0.5 mmol, 250 mM) using HATU (5 equiv., 0.5 mmol, 250 mM) and DIEA (10 equiv., 1.0 mmol, 500 mM) in 2 mL DMF for 45 minutes at RT. The resin was then loaded onto the CSBio automated peptide synthesizer and residues 58-93 were coupled using Fmoc-L-AA-OH (10 equiv., 1.0 mmol, 66.7 mM), HBTU/HOBt (10 equiv. each, 1.0 mmol, 66.7 mM), and DIEA (20 equiv., 2.0 mmol, 133.3 mM) in 15 mL DMF for 15 minutes at 60 °C per amino acid, followed by an Fmoc deprotection step. Fmoc-L-hArg(Pbf)-OH was coupled (5 equiv., 0.5 mmol, 250 mM) using HATU (5 equiv., 0.5 mmol, 250 mM), and DIEA (5 equiv., 0.5 mmol, 250 mM) in 2 mL DMF for 45 minutes at RT. Residues 53-56 were coupled using Fmoc-L-AA-OH and the same coupling cocktail. Finally, Boc-L-Cys(Acm)-OH (10 equiv., 1.0 mmol, 500 mM) was then coupled using HATU (10 equiv., 1.0 mmol, 500 mM), and DIEA (10 equiv., 1.0 mmol, 500 mM) in 2 mL DMF for 1 hour at

RT. To obtain segment **34**, 0.05 mmol of the peptide bound resin was washed with DMF (5 mL x 3), MeOH (5 mL x 3), and DCM (5 mL x 3) and was subjected to alloc removal using a mixture of $Pd(PPh_3)_4$ (1 equiv.), piperidine (640 μL), and DCM (2560 μL) that was shaken with the resin for 30 minutes at RT under the exclusion of light. The resin was washed with DCM (6 mL x 6) and DMF (6 mL x 6). Then, TAMRA (2 equiv., 0.1 mmol, 50 mM) was coupled using HATU (2 equiv., 0.1 mmol, 50 mM) and DIEA (4 equiv., 0.2 mmol, 100 mM) in 2 mL DMF for 2 hours at RT. Finally, the peptide-bound resin was washed with DMF (5 mL x 3), MeOH (5 mL x 3), and DCM (5 mL x 3) and dried under vacuum. To obtain segment **33**, the same process was repeated with the rest of the resin (0.05 mmol) without the TAMRA coupling step. To remove side chain protecting groups and release the peptide chains, a mixture of TFA/H_2O/TIS (95:2.5:2.5, 10 mL for 0.025 mmol scale) was added to each resin and shaken for 4 hours at RT. The resins were removed by filtration and extracted with TFA (2 × 1 mL). To precipitate the peptides, each filtrate was added dropwise to cold diethyl ether (15 mL for 0.025 mmol resin) followed by centrifugation at 4000 rpm for 7 min. Then, the diethyl ether was decanted, followed by the dissolution of the peptide in 50% ACN/water, dilution to 25% ACN/water, and lyophilization to obtain pink or white powders. The dry crude powders were purified by RP-HPLC (Method A described in Section 1.3) affording the products in the following yields: **33** (21.9 mg, 4.1 μmol, 16% yield based on 0.025 mmol resin), **34** (20.2 mg, 3.5 μmol, 14% yield based on 0.025 mmol resin).

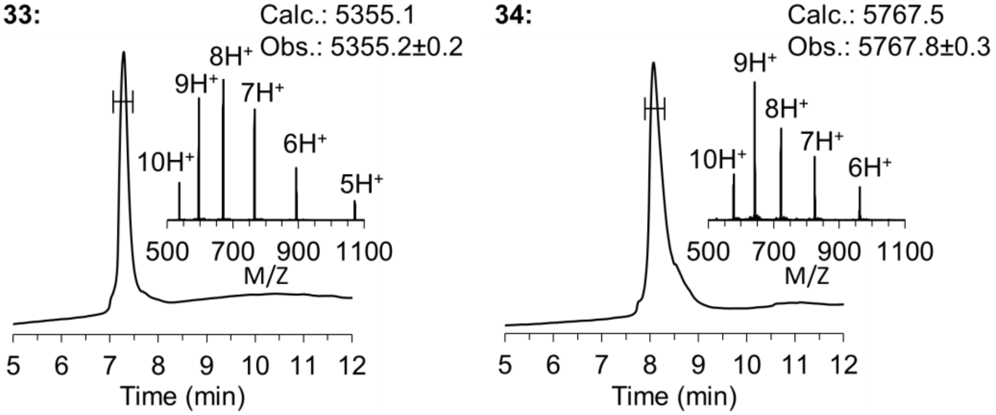

Figure S9. LCMS analysis of segments 33 and 34. LC of the UV absorbance at 214 nm and mass-to-charge (M/Z) spectrum. LCMS analysis was carried out with Method A (see section 1.2). M/Z data were acquired over the marked regions in the chromatograms. Calculated and observed masses are reported in Da (average isotopes).

5. Chemical synthesis of μMax variants

5.1 Synthesis of μMax variants via one-pot native chemical ligation and desulfurization

The reaction was carried out according to the following scheme:

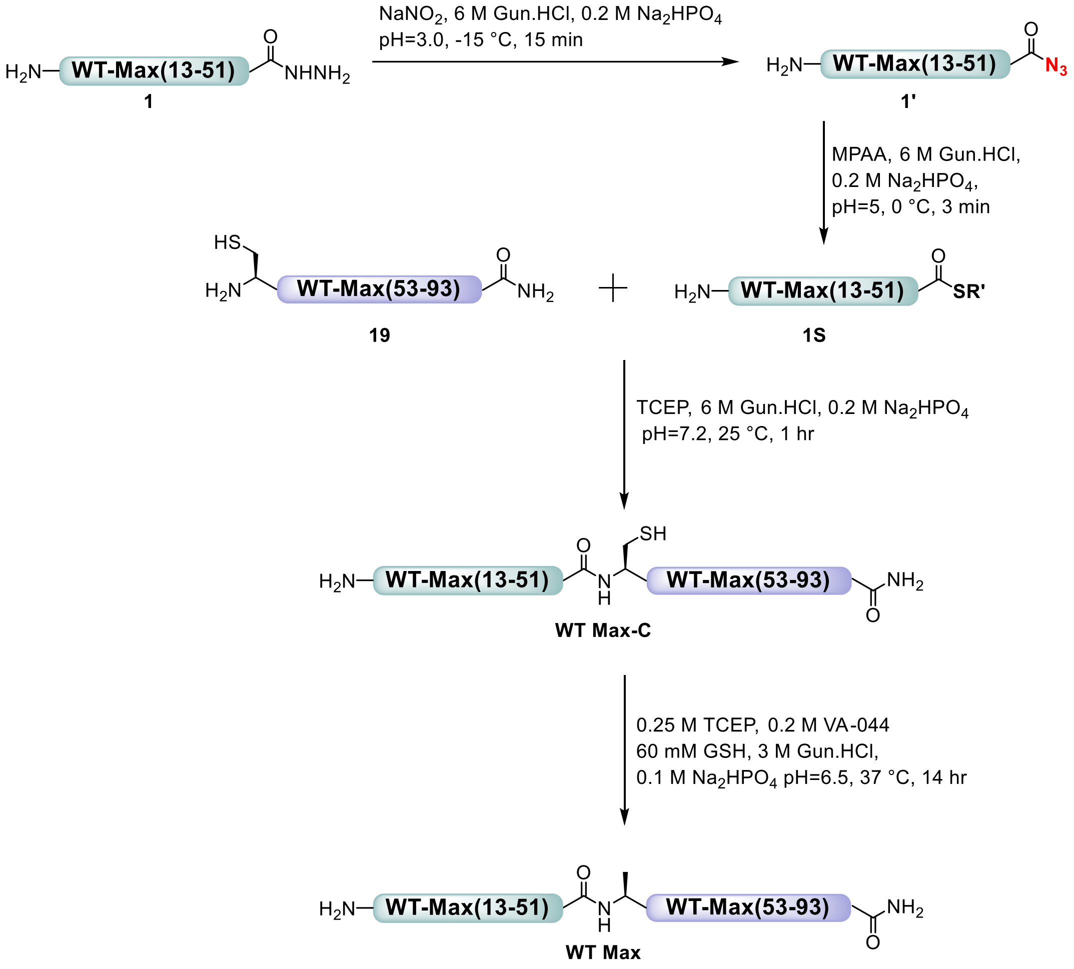

In a 1.5 mL Eppendorf, segment **1** Max(13-51)-NHNH2 (2.1 mg, 1.5 equiv., 0.45 μmol, 8.8 mM) was dissolved in 50 μL of 6 M Gun.HCl, 0.2 M Na_2HPO_4 buffer (NCL buffer) at pH=3.0 and cooled down to -15 °C by placing it in an ice/salt bath. 5 μL of $NaNO_2$ (10 equiv. based on **1**, 4.5 μmol, 0.9 M) dissolved in water was added to the reaction mixture and allowed to react for 15 min at -15 °C with gentle mixing in repeated intervals.[2,3] After 15 min, 50 μL of MPAA (50 equiv. based on **1**, 22.5 μmol, 0.45 M) in NCL buffer at pH=7 were added to the mixture and gently mixed for three minutes in an ice bath. Then, 50 μL of TCEP (50 equiv. based on **1**, 22.5 μmol, 0.45 M) in NCL buffer at pH=7 were added to the reaction mixture followed by the addition of the reaction mixture to segment **19** Cys-

Max(53-93) (1.5 mg, 1 equiv. 0.3 µmol).[2,3] The pH of the reaction mixture was then adjusted to 7.2 using 5 *N* NaOH. The final concentration of the segments was: (**1**) 3 mM and (**19**) 2 mM. The mixture was incubated at 25 °C and the reaction was monitored using LCMS (Method A described in Section 1.2). After 1 hour of ligation, the crude reaction was desalted by pipetting the reaction mixture into a 3 kDa molecular weight cutoff spin filter (Amicon® Ultra- 2mL, 3K). The reaction mixture was diluted with a 3 M Gun.HCl, 0.1 M Na_2HPO_4 buffer (pH=7.2) to 2.0 mL and concentrated to 1.0 mL by centrifuging the spin filter at 5000 rpm for 15 min. This process was repeated five more times until all MPAA was removed. In the final round, the reaction mixture was concentrated to 250 µL. After that, the reaction mixture was collected by reverse centrifuge and then treated with TCEP (250 equiv. based on **19**, 75 µmol) followed by the addition of VA-044 (200 equiv. based on **19**, 60 µmol). 50 µL of L-Glutathione (GSH, 60 equiv. based on **19**, 18 µmol, 0.36 M) in 3 M Gun.HCl, 0.1 M Na_2HPO_4 buffer were added, the pH was adjusted to 6.5, and the reaction was incubated for 14 hours at 37 °C.[4,5] The progress of the reaction was monitored using LCMS (Method A described in Section 1.2). After the completion of the reaction, purification was carried out using RP-HPLC (Method D described in Section 1.3) affording the final product **WT Max** (1.0 mg, 0.10 µmol, 33% yield, based on the limiting segment **19**).

The same protocol was used in a ~2.5 mg scale of the limiting segments **19**, **21**, or **22** to obtain the final products **µMax1-22**: **µMax1** (1.6 mg, 0.17 µmol, 34% yield), **µMax2** (1.0 mg, 0.11 µmol, 22% yield), **µMax3** (0.9 mg, 0.10 µmol, 20% yield), **µMax4** (0.9 mg, 0.10 µmol, 20% yield), **µMax5** (0.9 mg, 0.10 µmol, 20% yield), **µMax6** (0.8 mg, 0.08 µmol, 16% yield), **µMax7** (0.7 mg, 0.07 µmol, 14% yield), **µMax8** (1.5 mg, 0.16 µmol, 32% yield), **µMax9** (1.1 mg, 0.12 µmol, 24% yield), **µMax10** (1.6 mg, 0.17 µmol, 34% yield), **µMax11** (1.0 mg, 0.11 µmol, 22% yield), **µMax12** (0.5 mg, 0.05 µmol, 10% yield), **µMax13** (1.1 mg, 0.12 µmol, 24% yield), **µMax14** (0.6 mg, 0.06 µmol, 12% yield), **µMax15** (0.8 mg, 0.08 µmol, 16% yield), **µMax16** (1.2 mg, 0.13 µmol, 26% yield), **µMax17** (1.1 mg, 0.12 µmol, 24% yield), **µMax18** (1.1 mg, 0.12 µmol, 24% yield), **µMax19** (0.8 mg, 0.09 µmol, 18% yield), **µMax20** (1.5 mg, 0.15 µmol, 32% yield), **µMax21** (1.6 mg, 0.16 µmol, 34% yield), and **µMax22** (1.5 mg, 0.15 µmol, 32% yield).

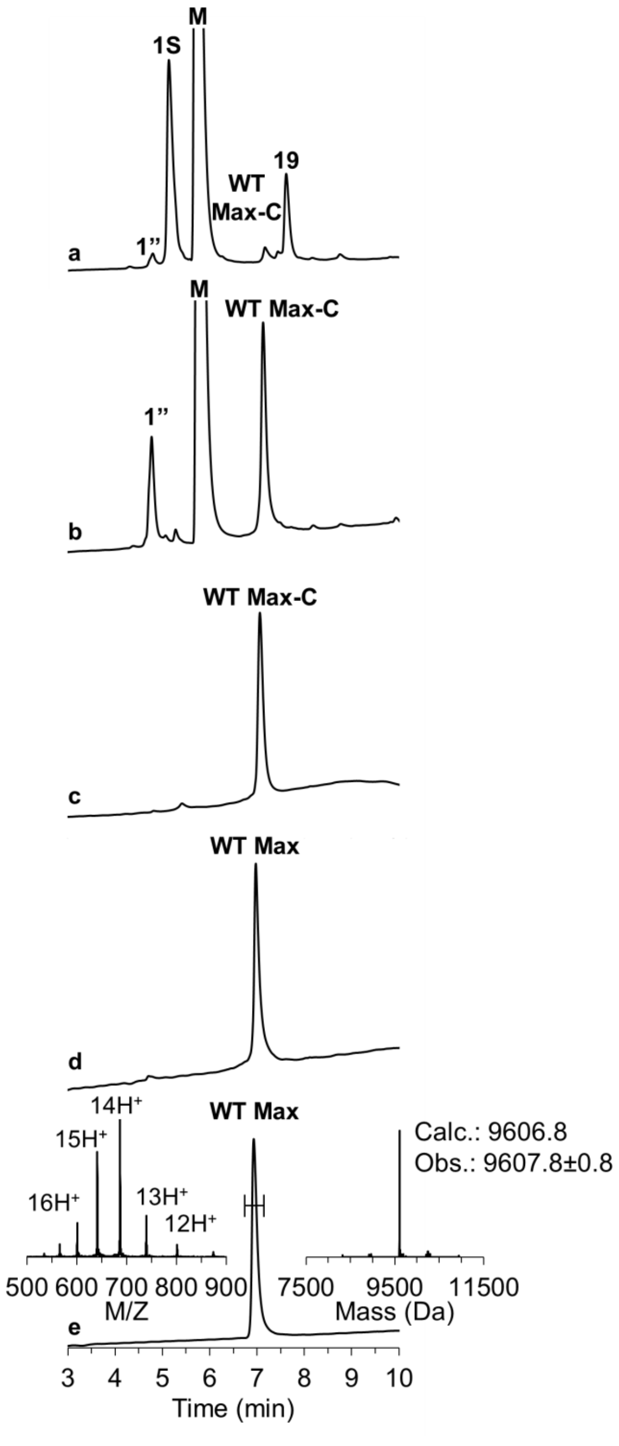

Figure S10. LCMS analysis of the progress of segments 1 and 19 one-pot ligation and desulfurization (WT Max). a) Ligation at t=0 min; segment 1 thioester (**1S**), hydrolysis of segment 1 thioester (**1’’**), segment **19**, ligated product (**WT Max-C**), and M=MPAA. b) Crude ligation reaction at t=60 min. c) Crude ligation reaction after desalting. d) Crude reaction after 14 hours desulfurization; desulfurized ligation product (**WT Max**). e) RP-HPLC purified final product **WT Max**. LC of the UV absorbance at 214 nm, mass-to-charge (M/Z) spectrum, and deconvoluted spectrum. LCMS analysis was carried out with Method A (see section 1.2). M/Z data was acquired over the marked region in the chromatogram. Calculated and observed masses are reported in Da (average isotopes).

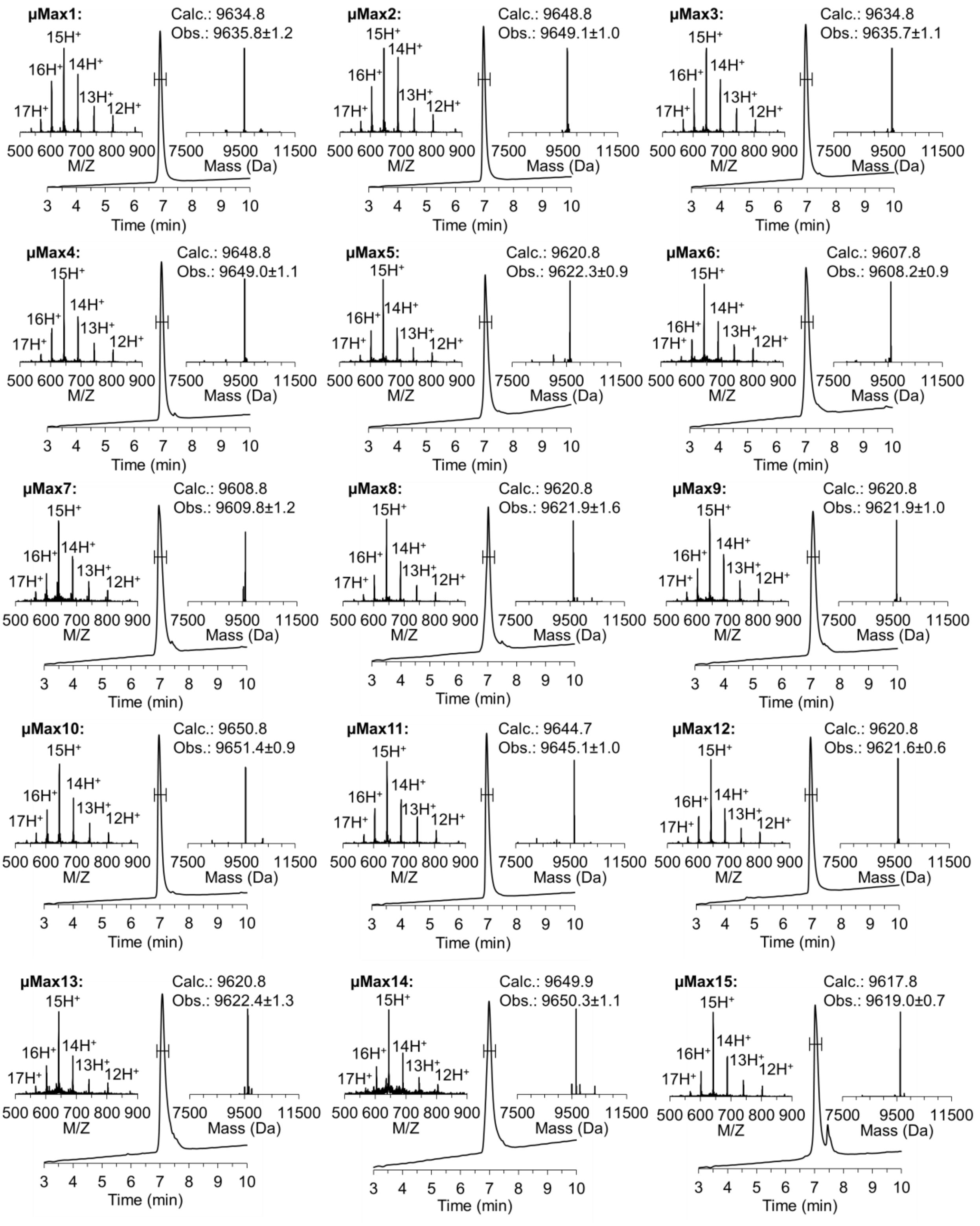

Figure S11. LCMS analysis of final products μMax1-15. LC of the UV absorbance at 214 nm, mass-to-charge (M/Z) spectrum, and deconvoluted spectrum. LCMS analysis was carried out with Method A (see section 1.2). M/Z data were acquired over the marked regions in the chromatograms. Calculated and observed masses are reported in Da (average isotopes).

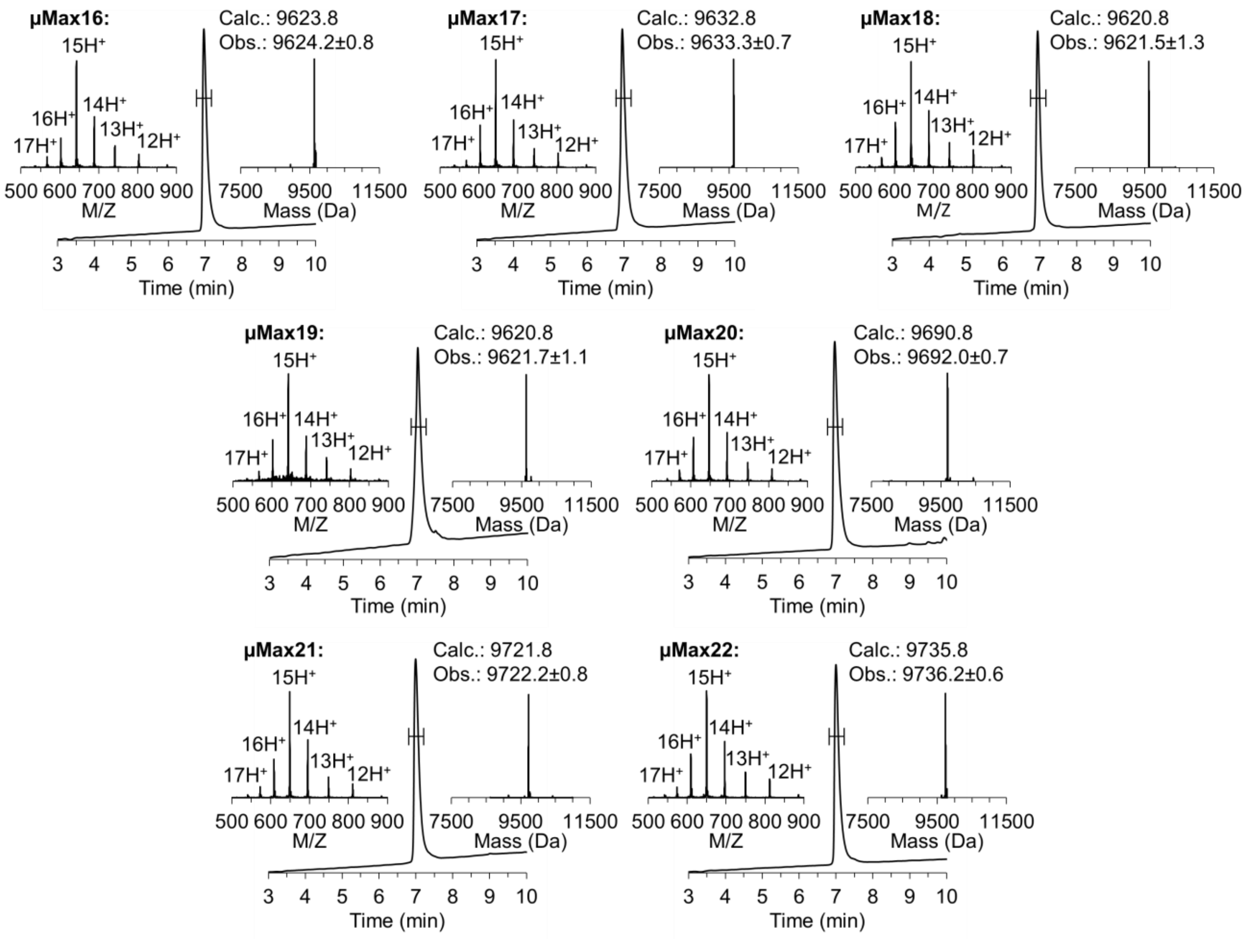

Figure S12. LCMS analysis of final products µMax16-22. LC of the UV absorbance at 214 nm, mass-to-charge (M/Z) spectrum, and deconvoluted spectrum. LCMS analysis was carried out with Method A (see section 1.2). M/Z data were acquired over the marked regions in the chromatograms. Calculated and observed masses are reported in Da (average isotopes).

5.2 Synthesis of TAMRA-labeled µMax variants via one-pot native chemical ligation and desulfurization

The reaction was carried out according to the following scheme:

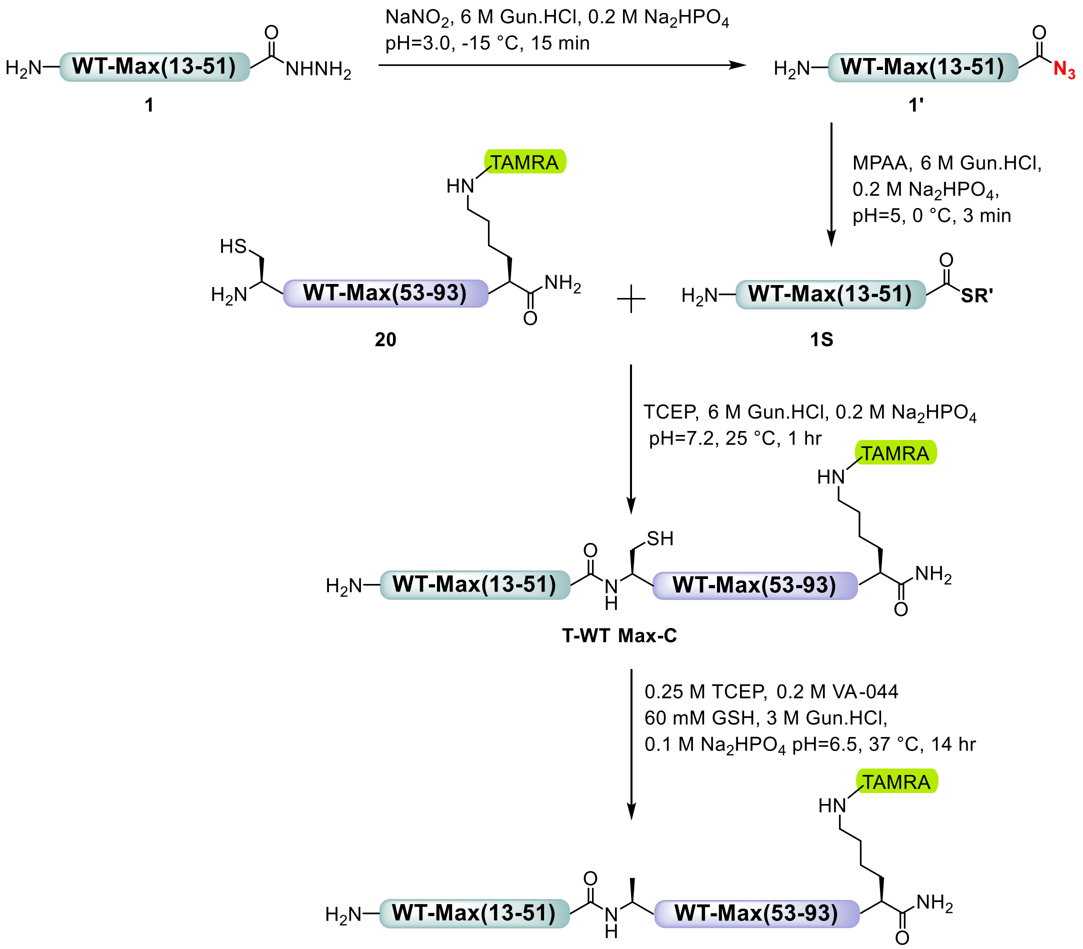

The ligation of the TAMRA-labeled products was carried out in the same manner as detailed in section 5.1 to obtain the following: **T-WT Max** (0.3 mg, 0.03 µmol, 7% yield), **T-µMax20** (0.2 mg, 0.02 µmol, 5% yield), and **T-µMax21** (0.3 mg, 0.03 µmol, 7% yield).

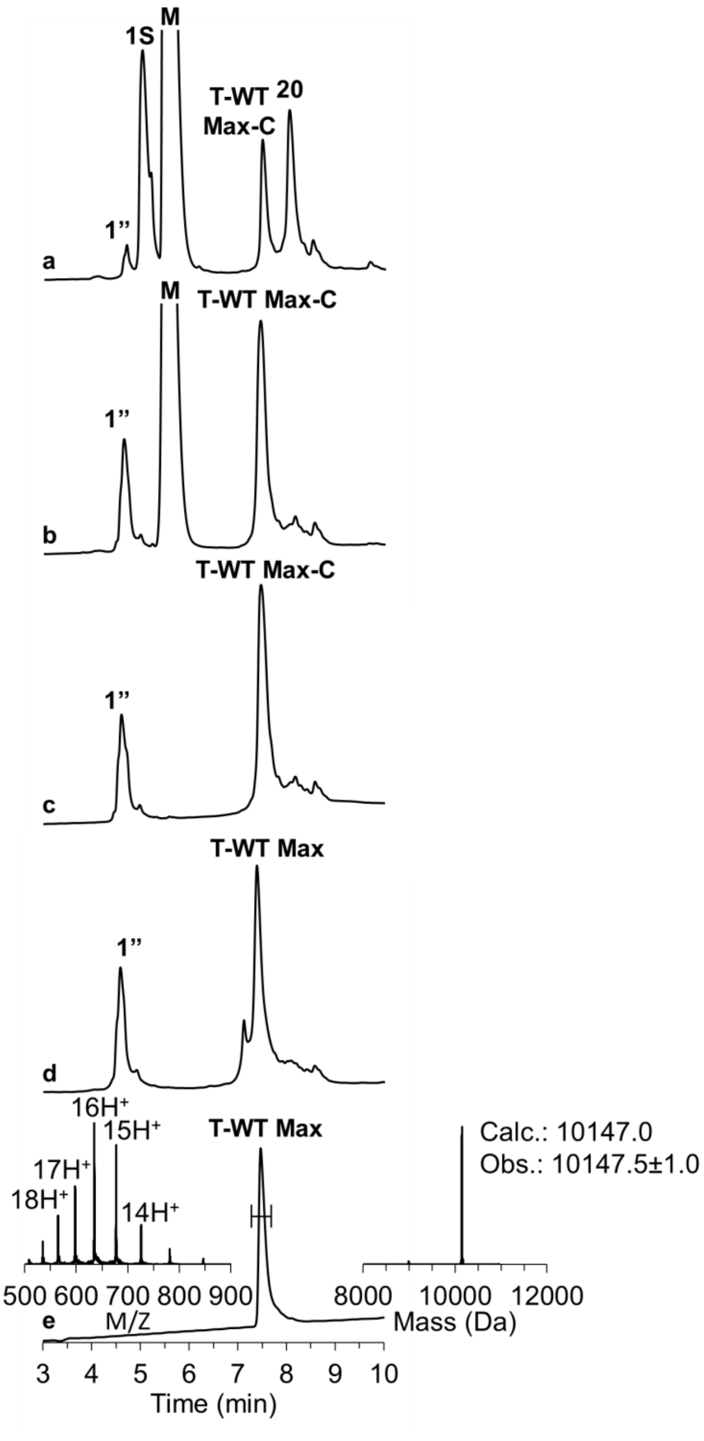

Figure S13. LCMS analysis of the progress of segments 1 and 20 one-pot ligation and desulfurization (**T-WT Max**)**.** a) Ligation at t=0 min; segment 1 thioester (**1S**), hydrolysis of segment 1 thioester (**1’’**), segment **20**, ligated product (**T-WT Max-C**), and M=MPAA. b) Crude ligation reaction at t=60 min. c) Crude ligation reaction after desalting. d) Crude reaction after 14 hours desulfurization; desulfurized ligation product (**T-WT Max**). e) RP-HPLC purified final product **T-WT Max**. LC of the UV absorbance at 214 nm, mass-to-charge (M/Z) spectrum, and deconvoluted spectrum. LCMS analysis was carried out with Method A (see section 1.2). M/Z data was acquired over the marked region in the chromatogram. Calculated and observed masses are reported in Da (average isotopes).

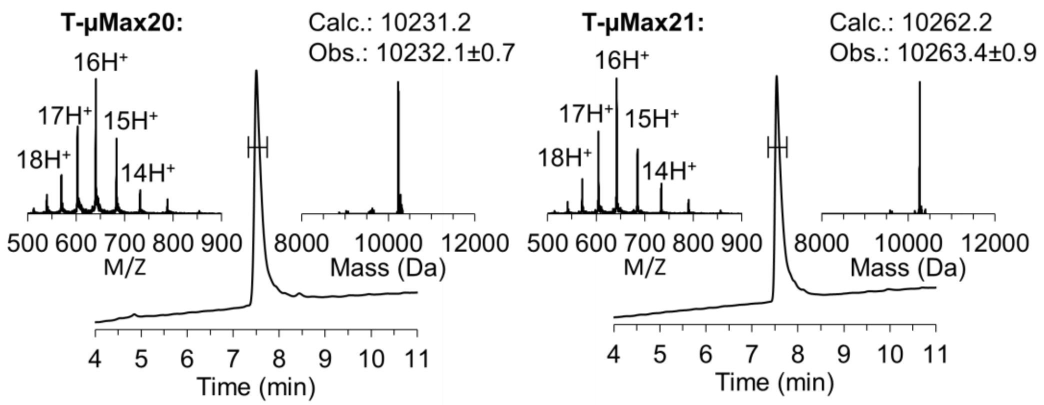

Figure S14. LCMS analysis of TAMRA-labeled final products T-μMax20-21. LC of the UV absorbance at 214 nm, mass-to-charge (M/Z) spectrum, and deconvoluted spectrum. LCMS analysis was carried out with Method A (see section 1.2). M/Z data were acquired over the marked regions in the chromatograms. Calculated and observed masses are reported in Da (average isotopes).

5.3 Chemical synthesis of stapled μMax 2S$_p$-μMax20

Note: The following stapling chemistries and positions were scanned, ultimately yielding the stapled analogs detailed in sections 5.3.1 and 5.4.1:
Staplers: Perfluorobenzene, 1,3-bis(bromomethyl)benzene, 2,6-bis(bromomethyl)pyridine, and α-methyl-phenylalanine.
Positions: 13-17; 17-21; 32-36; 81-85; 84-88; 88-92.

5.3.1 Native chemical ligation of 2S$_p$-μMax20 segments

The reaction was carried out according to the following scheme:

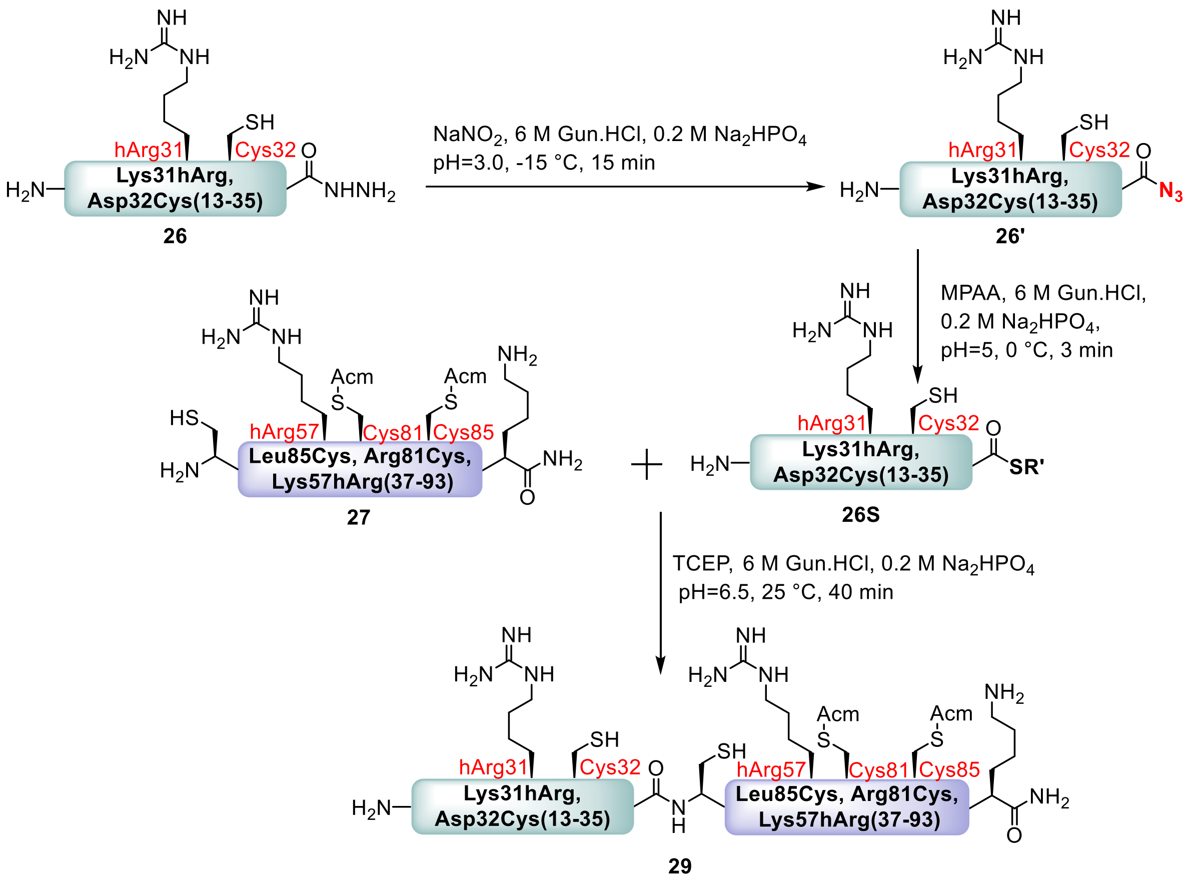

In 1.5 mL Eppendorf, segment **26** (8.4 mg, 1.6 equiv., 2.9 μmol) was dissolved in 330 μL of NCL buffer at pH=3.0 and cooled down to -15 °C by placing it in an ice/salt bath. 20 μL of $NaNO_2$ (10 equiv. based on **26**, 29.0 μmol, 1.5 M) dissolved in water were added to the reaction mixture and allowed to react for 15 min at -15 °C with gentle mixing in repeated intervals.[2,3] After 15 min, 330 μL of MPAA (50 equiv. based on **26**, 145.0 μmol, 0.4 M) in NCL buffer at pH=7 were added to the mixture and gently mixed for three minutes in an ice bath. 312 μL of TCEP (50 equiv. based on **26**, 145.0 μmol, 0.4 M) in

NCL buffer at pH=7 were added followed by the addition of segment **27** (13.0 mg, 1 equiv. 1.8 µmol). The pH was adjusted to 6.5 using 5 *N* NaOH and the mixture was incubated at 25 °C. The reaction was monitored using LCMS (Method A described in Section 1.2).[2,3] The final concentrations of the segments were: (**26**) 3 mM and (**27**) 2 mM. Upon reaction completion, the ligated product was purified using RP-HPLC (Method E described in Section 1.3) affording ligated product **29** (9.6 mg, 0.97 µmol, 54% yield, based on the limiting segment **27**).

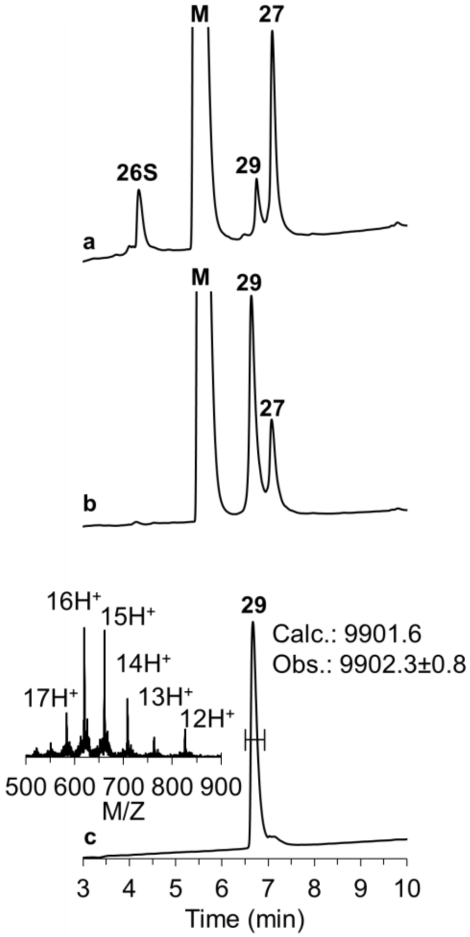

Figure S15. LCMS analysis of the progress of segments 26 and 27 ligation. a) Ligation at t=0 min; segment 26 thioester (**26S**), segment **27**, ligated product (**29**), and M=MPAA. b) Crude ligation reaction at t=40 min. c) RP-HPLC purified product **29**. LC of the UV absorbance at 214 nm and mass-to-charge (M/Z) spectrum. LCMS analysis was carried out with Method A (see section 1.2). M/Z data was acquired over the marked region in the chromatogram. Calculated and observed masses are reported in Da (average isotopes).

5.3.2 Stapling of segment 29

The reaction was carried out according to the following scheme:

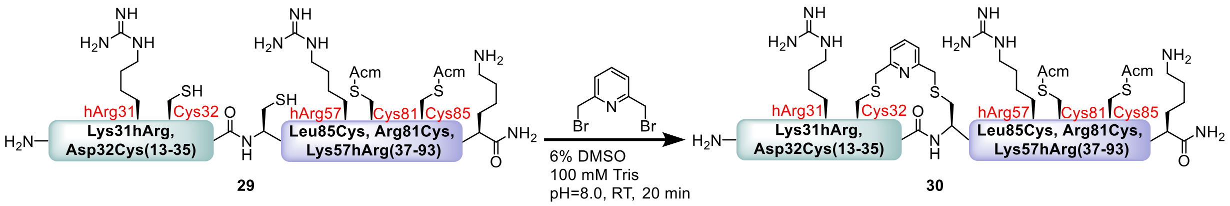

In a 1.5 mL Eppendorf, segment **29** (1 equiv., 8.5 mg, 0.86 µmol) was dissolved in 807 µL of 100 mM Tris in H_2O pH=8.0. 17.1 µL of 2,6-bis(bromomethyl)pyridine in DMSO were added 3 times every 2 minutes (total of 20 equiv., 17.2 µmol, 20 mM).[6] The final concentration of segment **29** was 1 mM in the presence of 6% DMSO. The reaction was incubated at room temperature for 20 min protected by aluminum foil and was monitored using LCMS (Method A described in Section 1.2). Upon reaction completion, 10 µL of 5 *N* HCl were added to quench it. The crude was then purified using RP-HPLC (Method E described in Section 1.3) affording the stapled product **30** (4.0 mg, 0.40 µmol, 47% yield).

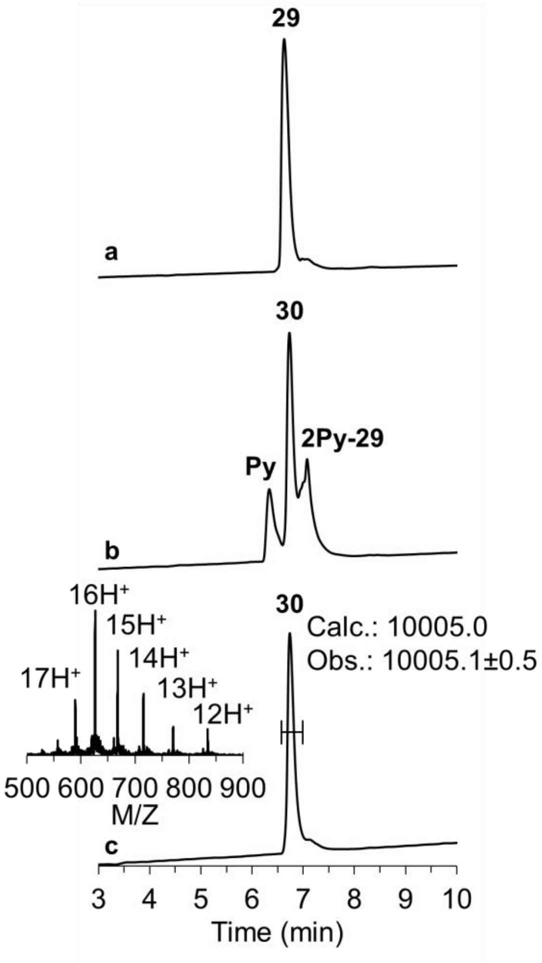

Figure S16. LCMS analysis of the progress of segment 29 stapling. a) Starting material at t=0 min; segment **29**. b) Crude stapling reaction at t=20 min; product **30**, Py=2,6-bis(bromomethyl)pyridine, and dialkylation by-product (**2Py-29**). c) RP-HPLC purified product **30**. LC of the UV absorbance at 214 nm and mass-to-charge (M/Z) spectrum. LCMS analysis was carried out with Method A (see section 1.2). M/Z data was acquired over the marked region in the chromatogram. Calculated and observed masses are reported in Da (average isotopes).

5.3.3 Decaging of segment 30

The reaction was carried out according to the following scheme:

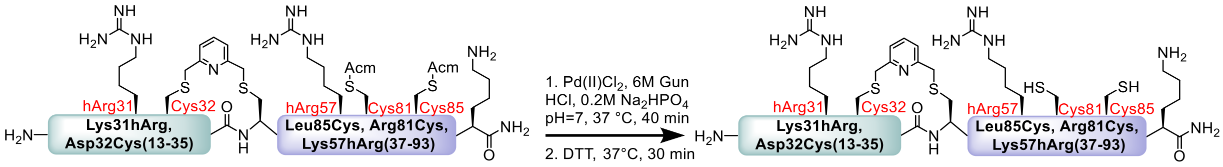

In a 1.5 mL Eppendorf, segment **30** (1 equiv., 4.0 mg, 0.4 μmol) was dissolved in 100 μL of NCL buffer. 100 μL of Pd(II)Cl_2 (20 equiv., 8.0 μmol, 80 mM) in NCL buffer were added in one portion and the pH was adjusted to 7.[7] The final concentration of segment **30** was 2 mM. The reaction was incubated at 37 °C for 40 min and was monitored using LCMS (Method A described in Section 1.2). After the reaction was completed, DTT (3 equiv. based on Pd(II)Cl_2, 3.7 mg, 24.0 μmol) was added and the crude turned bright orange. The reaction was kept for 30 minutes at 37 °C. The crude was diluted to 600 μL using 0.1% TFA in H_2O in portions which led to massive precipitation of an orange solid. The reaction was centrifuged, and the pellet was recovered using 50 μL of 50% ACN/H_2O with 0.1% TFA. The recovery process was repeated 2 more times. Finally, the combined reaction crude was purified using RP-HPLC (Method C described in Section 1.3) affording the decaged product **31** (1.9 mg, 0.19 μmol, 48% yield).

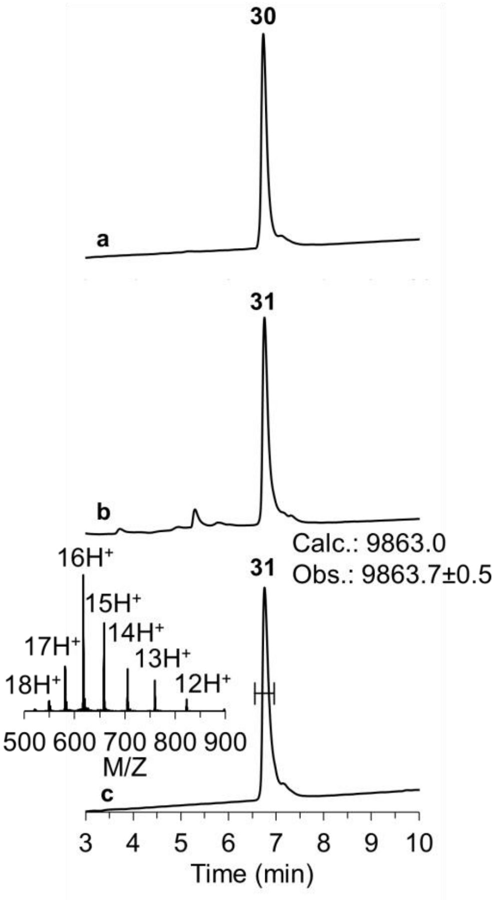

Figure S17. LCMS analysis of the progress of segment 30 decaging. a) Acm removal at t=0 min; segment **30**. b) Crude stapling reaction at t=40 min; product **31**. c) RP-HPLC purified product **31**. LC of the UV absorbance at 214 nm and mass-to-charge (M/Z) spectrum. LCMS analysis was carried out with Method A (see section 1.2). M/Z data was acquired over the marked region in the chromatogram. Calculated and observed masses are reported in Da (average isotopes).

5.3.4 Stapling of segment 31

The reaction was carried out according to the following scheme:

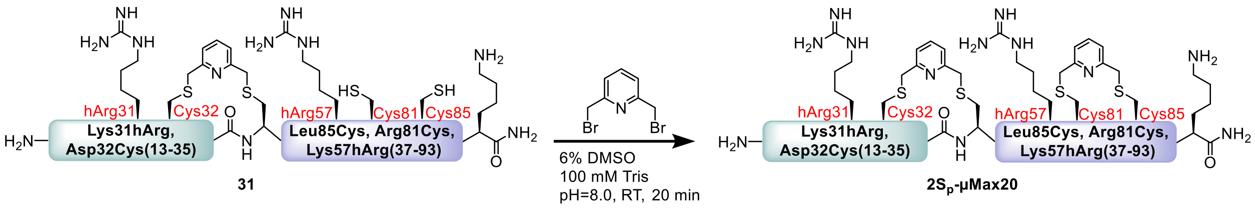

The stapling of **31** was carried out in the same manner as detailed in section 5.3.2 to obtain the final product **2S$_p$-µMax20** (0.7 mg, 0.07 µmol, 26% yield based on the limiting segment **31**).

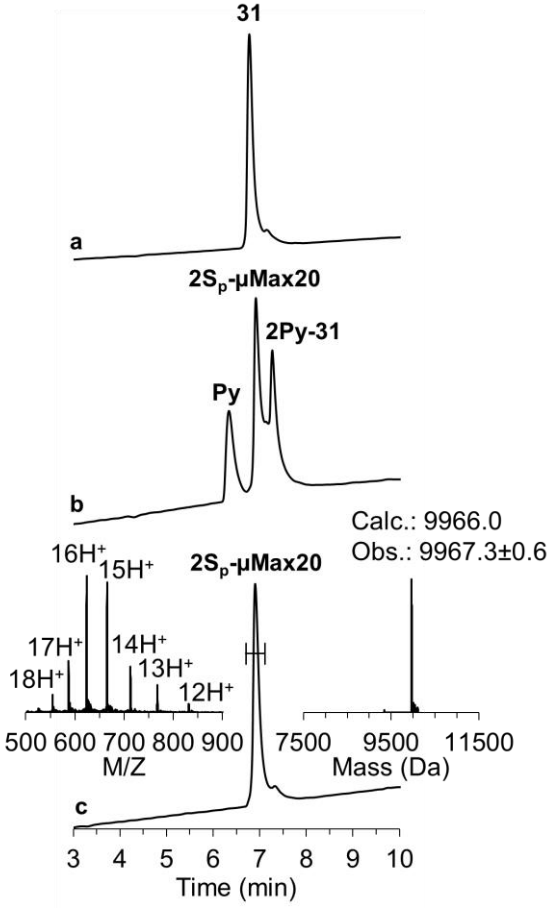

Figure S18. LCMS analysis of the progress of segment 31 stapling. a) Starting material at t=0 min; segment **31**. b) Crude stapling reaction at t=20 min; product **2S$_p$-µMax20**, Py=2,6-bis(bromomethyl)pyridine, and dialkylation by-product (**2Py-31**). c) RP-HPLC purified product **2S$_p$-µMax20**. LC of the UV absorbance at 214 nm, mass-to-charge (M/Z) spectrum, and deconvoluted spectrum. LCMS analysis was carried out with Method A (see section 1.2). M/Z data was acquired over the marked region in the chromatogram. Calculated and observed masses are reported in Da (average isotopes).

5.3.5 Synthesis of T-2S_p-µMax20

The synthesis of the TAMRA-labeled double pyridine stapled product, **T-2S_p-µMax20**, was performed in a similar manner as detailed in sections 5.3.1-5.3.4, starting with the ligation of segment **26** with segment **28**.

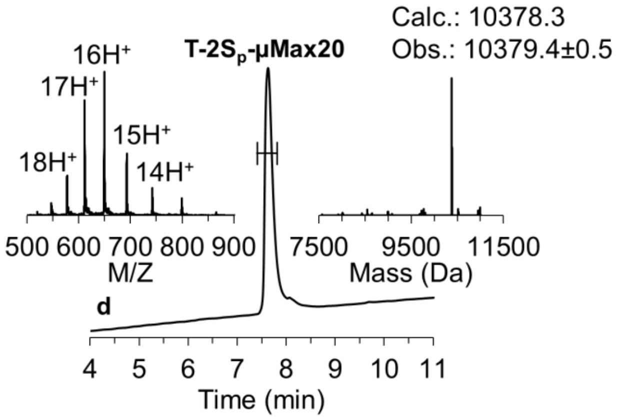

Figure S19. LCMS analysis of T-2S_p-µMax20. LC of the UV absorbance at 214 nm, mass-to-charge (M/Z) spectrum, and deconvoluted spectrum. LCMS analysis was carried out with Method A (see section 1.2). M/Z data was acquired over the marked region in the chromatogram. Calculated and observed masses are reported in Da (average isotopes).

5.4 Chemical synthesis of stapled μMax 2S$_b$-μMax20
5.4.1 Stapling of segment 32

The reaction was carried out according to the following scheme:

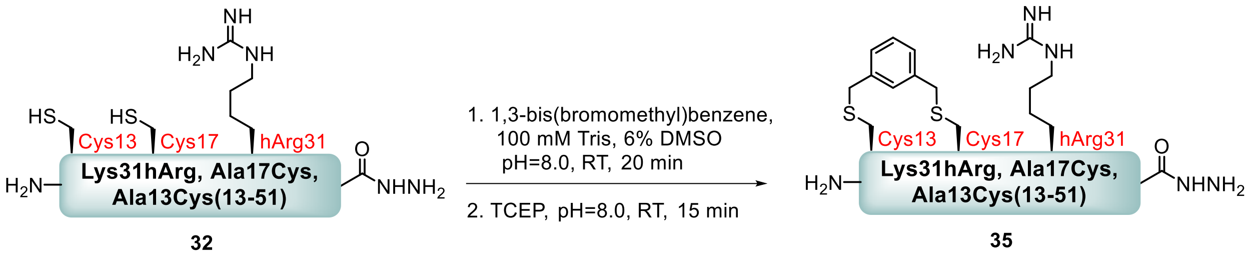

In a 4 mL glass vial equipped with a magnetic stirring bar, segment **32** (1 equiv., 22.7 mg, 4.86 μmol) was dissolved in 2285 μL of 100 mM Tris in H_2O pH=8.0. 36.4 μL of 1,3-bis(bromomethyl)benzene in DMSO were added dropwise 4 times every 2 minutes (total of 2 equiv., 9.89 μmol, 68 mM) resulting in a cloudy white suspension. The final concentration of segment **32** was 2 mM in the presence of 6% DMSO. The reaction was incubated at room temperature for 20 min protected by aluminum foil and was monitored using LCMS (Method A described in Section 1.2). Then, 227 μL of TCEP (7 equiv. 34.0 μmol, 0.15 M) in 100 mM Tris in H_2O pH=8.0 were added to reduce disulfide bonds and mixed at room temperature for 15 min. After 15 min the solution became transparent. The crude was then purified using RP-HPLC (Method A described in Section 1.3) affording the stapled product **35** (9.3 mg, 1.95 μmol, 40% yield).

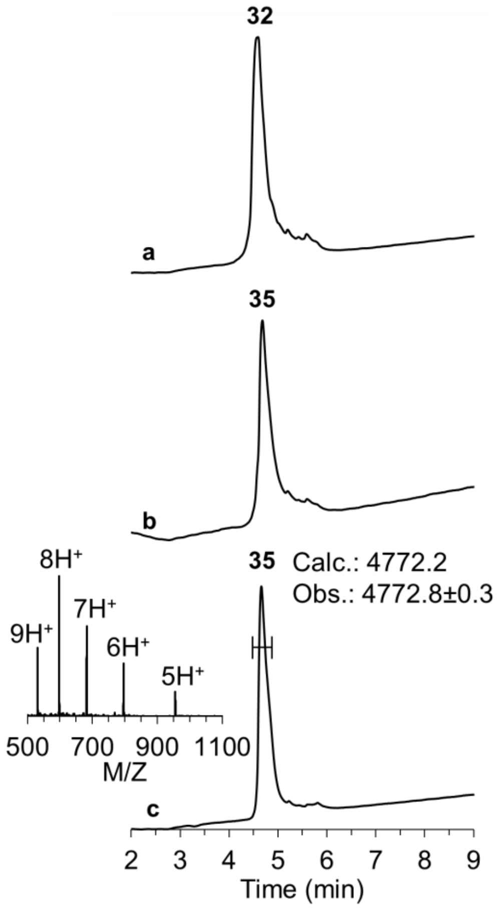

Figure S20. LCMS analysis of the progress of segment 32 stapling. a) Starting material at t=0 min; segment **32**. b) Crude stapling reaction after addition of TCEP and mixing for 15 min; product **35**. c) RP-HPLC purified product **35**. LC of the UV absorbance at 214 nm and mass-to-charge (M/Z) spectrum. LCMS analysis was carried out with Method A (see section 1.2). M/Z data was acquired over the marked region in the chromatogram. Calculated and observed masses are reported in Da (average isotopes).

5.4.2 One-pot stapling and decaging of segment 33

The reaction was carried out according to the following scheme:

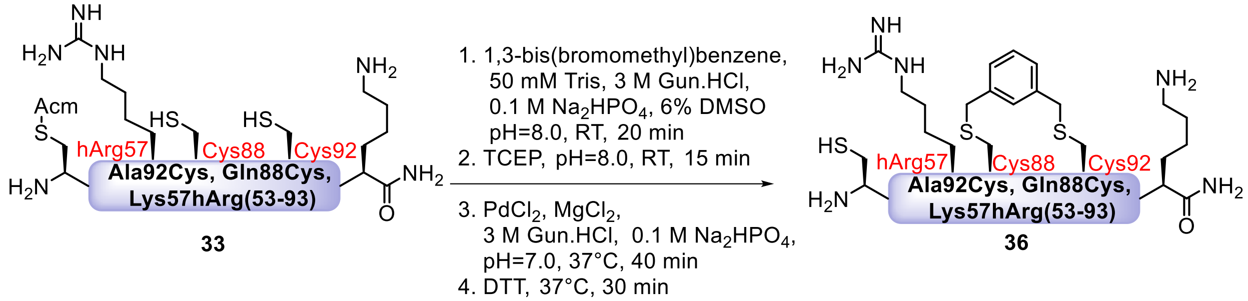

In a 4 mL glass vial equipped with a magnetic stirring bar, segment **33** (1 equiv., 10.0 mg, 1.87 µmol) was dissolved in 438 µL of NCL buffer and 438 µL of 100 mM Tris in H_2O pH=8.0. 13.2 µL of 1,3-bis(bromomethyl)benzene in DMSO were added dropwise 4 times every 2 minutes (total of 3 equiv., 5.6 µmol, 0.11 M) resulting in a cloudy white suspension. The final concentration of segment **33** was 2 mM in the presence of 6% DMSO. The reaction was incubated at room temperature for 20 min protected by aluminum foil and was monitored using LCMS (Method A described in Section 1.2). Then, 100 µL of TCEP (7 equiv. 13.1 µmol, 0.13 M) in 100 mM Tris in H_2O pH=8.0 were added to reduce disulfide bonds and mixed at room temperature for 15 min. After 15 min the solution became transparent. Then, 464 µL of $MgCl_2$ (50 equiv. 93.5 µmol, 0.2 M) in NCL buffer were added and the reaction mixture was mixed for 10 minutes at 37 °C in a water bath. Parallelly, $PdCl_2$ (10 equiv., 3.3 mg, 18.7 µmol) was dissolved in 464 µL of NCL buffer and incubated at 37 °C for 10 minutes until the salt was fully dissolved. Upon 10 minutes, the $PdCl_2$ solution was added to the reaction mixture and mixed at 37 °C for 40 minutes. Finally, 200 µL of DTT (50 equiv. 93.5 µmol, 0.47 M) in NCL buffer were added to quench the reaction. The mixture was mixed at 37 °C for 30 minutes resulting in an orange suspension. The reaction mixture was acidified to pH=3.0 using 5 N HCl and mixed at 37 °C for 10 more minutes. Then, the suspension was centrifuged at 5000 rpm for 5 minutes and the pellet was recovered with 50% ACN/H_2O solution containing 0.1% TFA (150 µL X2). The combined supernatants containing **36** were then purified using RP-HPLC (Method C described in Section 1.3) affording the stapled product **36** (4.3 mg, 0.80 µmol, 43% yield).

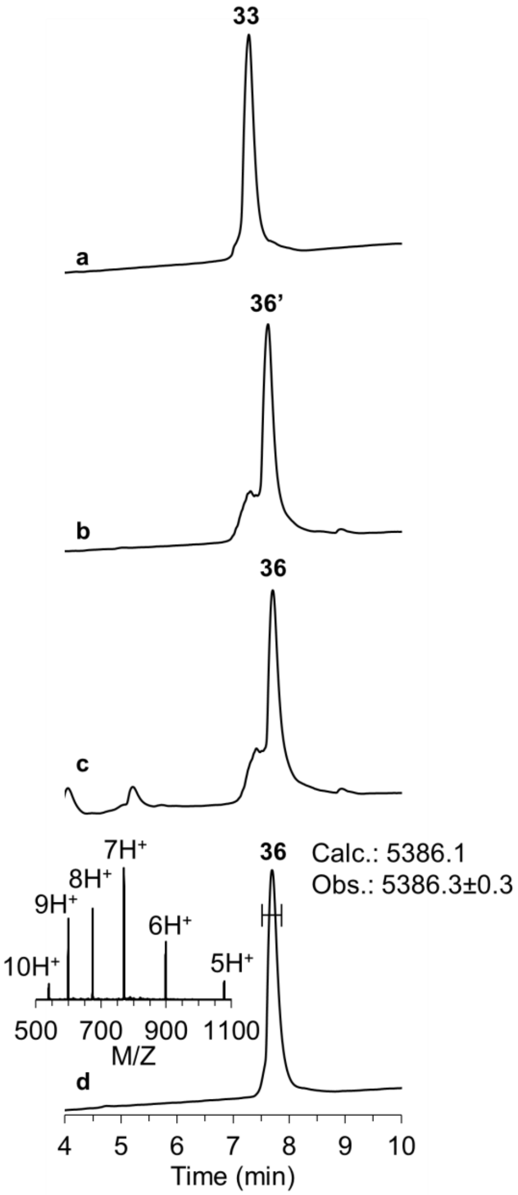

Figure S21. LCMS analysis of the progress of segment 33 stapling and decaging. a) Starting material at t=0 min; segment **33**. b) Crude stapling reaction after addition of TCEP and mixing for 15 min; stapled caged product **36'**. c) Crude decaging reaction after quenching with DTT and mixing for 40 min; stapled decaged product **36**. d) RP-HPLC purified stapled and decaged product **36**. LC of the UV absorbance at 214 nm and mass-to-charge (M/Z) spectrum. LCMS analysis was carried out with Method A (see section 1.2). M/Z data was acquired over the marked region in the chromatogram. Calculated and observed masses are reported in Da (average isotopes).

5.4.3 Synthesis of 2Sb-μMax20 via one-pot native chemical ligation and desulfurizaiton

The reaction was carried out according to the following scheme:

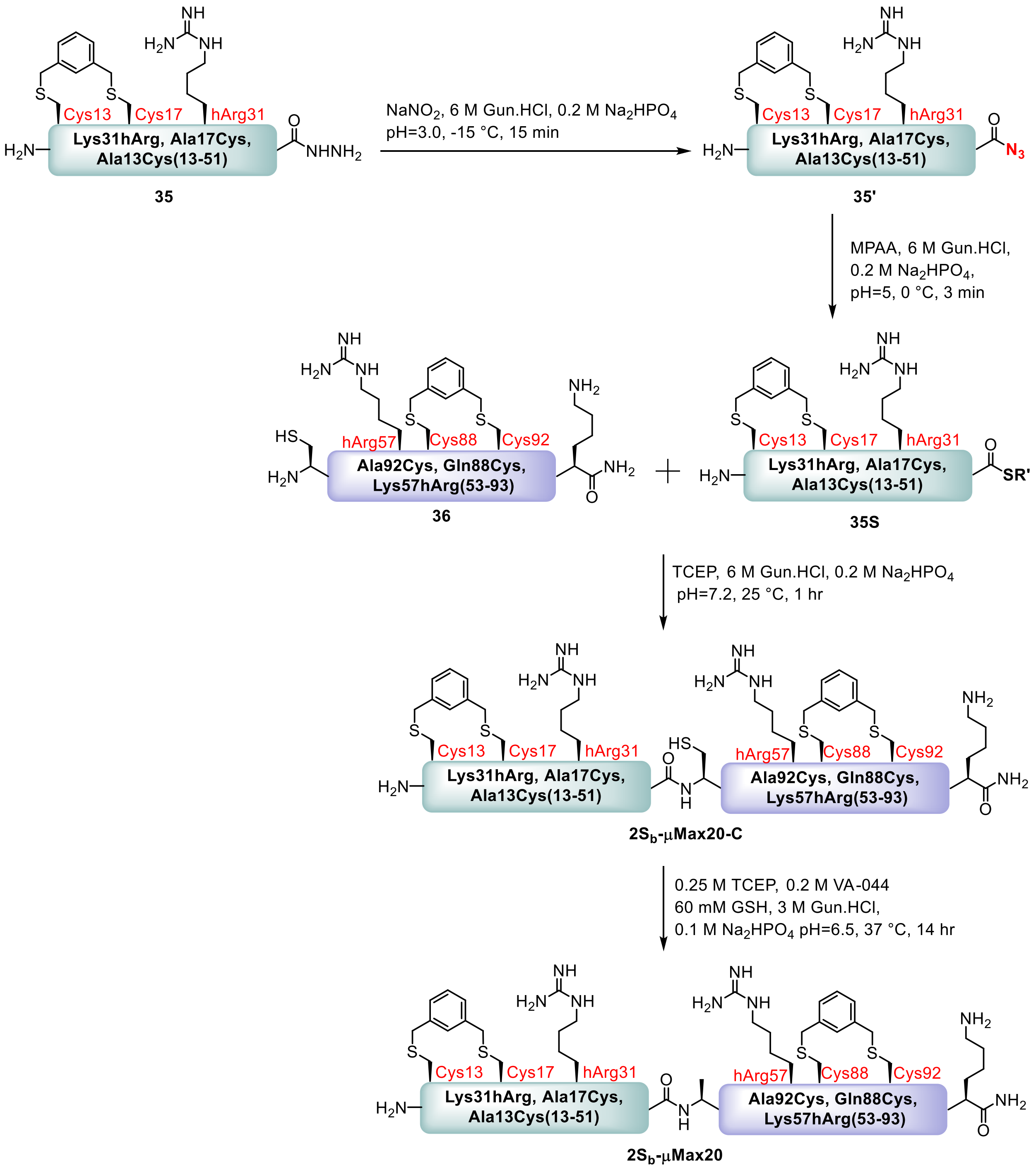

In a 1.5 mL Eppendorf, segment **35** (1.3 equiv., 5.2 mg, 1.1 μmol) was dissolved in 195 μL of NCL buffer at pH=3.0 and cooled down to -15 °C by placing it in an ice/salt bath.

20.0 µL of $NaNO_2$ (10 equiv. based on **35**, 11.0 µmol, 0.55 M) dissolved in water were added to the reaction mixture and allowed to react for 15 min at -15 °C with gentle mixing in repeated intervals.[2,3] After 15 min, 195 µL of MPAA (50 equiv. based on **35**, 55.0 µmol, 0.3 M) in NCL buffer at pH=7 were added to the mixture and gently mixed for three minutes in an ice bath. Then, the reaction mixture was added to segment **36** (4.5 mg, 1 equiv. 0.8 µmol).[2,3] The pH of the reaction mixture was then adjusted to 7.2 using 5 *N* NaOH. The final concentration of the segments was: (**35**) 2.6 mM and (**36**) 2 mM. The mixture was incubated at 25 °C and the reaction was monitored using LCMS (Method A described in Section 1.2). After 1 hour, 80 µL of TCEP (50 equiv. based on **35**, 55.0 µmol, 0.7 M) in NCL buffer at pH=7 were added to the crude reaction to reduce disulfide bonds, and the reaction was incubated at 25 °C for 15 minutes. The reaction was then desalted by pipetting it into a 3 kDa molecular weight cutoff spin filter (Amicon® Ultra- 2mL, 3K). The reaction mixture was diluted with a 3 M Gun.HCl, 0.1 M Na_2HPO_4 buffer (pH=7.2) to 2.0 mL and concentrated to 1.0 mL by centrifuging the spin filter at 5000 rpm for 15 min. This process was repeated five more times until all MPAA was removed. In the final round, the reaction mixture was concentrated to 245 µL. After that, the reaction mixture was collected by reverse centrifuge and then treated with TCEP (250 equiv. based on **36**, 200.0 µmol) followed by the addition of VA-044 (200 equiv. based on **36**, 160.0 µmol). 100 µL of L-Glutathione (GSH, 60 equiv. based on **36**, 48.0 µmol, 0.48 M) in 3 M Gun.HCl, 0.1 M Na_2HPO_4 buffer were added, the pH was adjusted to 6.5, and the reaction was incubated for 14 hours at 37 °C.[4,5] The progress of the reaction was monitored using LCMS (Method A described in Section 1.2). After the completion of the reaction, purification was carried out using RP-HPLC (Method E described in Section 1.3) affording the final product **2S$_b$-µMax20** (2.9 mg, 0.29 µmol, 36% yield, based on the limiting segment **36**).

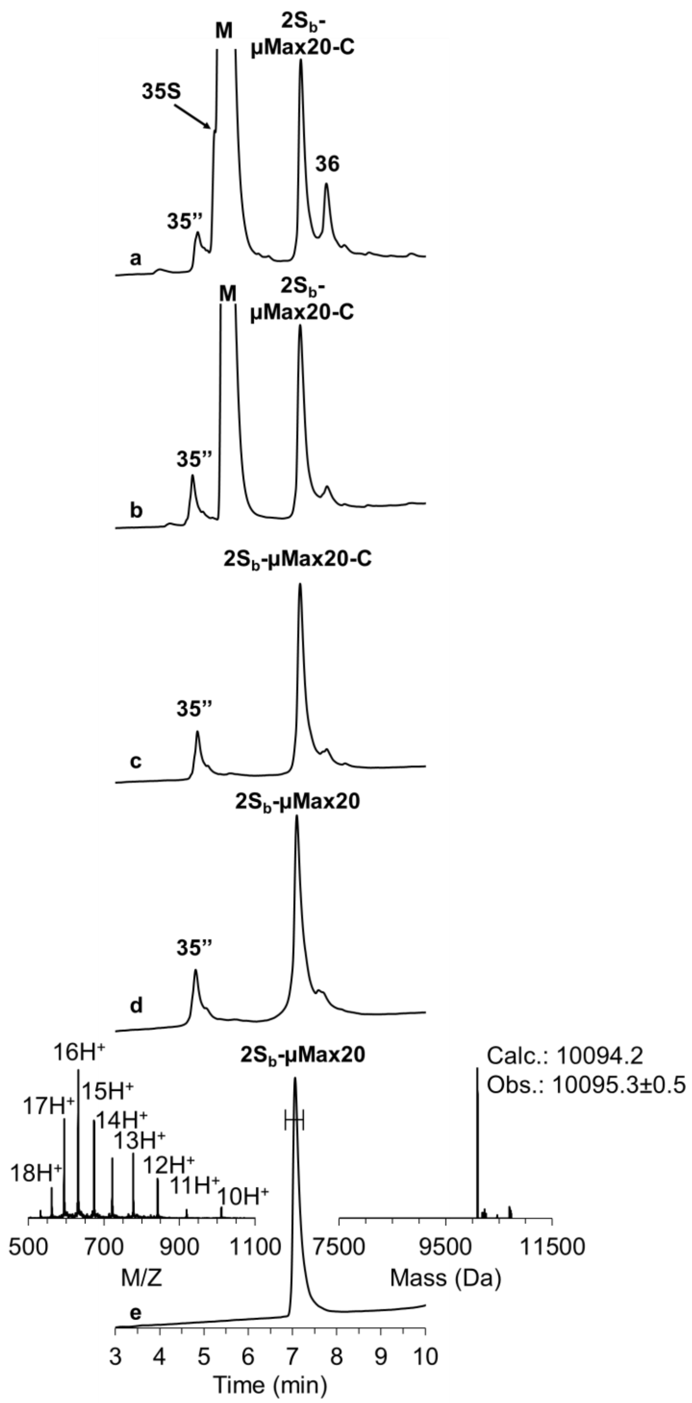

Figure S22. LCMS analysis of the progress of segments 35 and 36 one-pot ligation and desulfurization (2S$_b$-μMax20). a) Ligation at t=0 min; segment 35 thioester (**35S**), hydrolysis of segment 35 thioester (**35”**), segment **36**, ligated product (**2S$_b$-μMax20-C**), and M=MPAA. b) Crude ligation reaction after the addition of TCEP. c) Crude ligation reaction after desalting. d) Crude reaction after 14 hours desulfurization; desulfurized ligation product (**2S$_b$-μMax20**). e) RP-HPLC purified final product **2S$_b$-μMax20**. LC of the UV absorbance at 214 nm, mass-to-charge (M/Z) spectrum, and deconvoluted spectrum. LCMS analysis was carried out with Method A (see section 1.2). M/Z data was acquired over the marked region in the chromatogram. Calculated and observed masses are reported in Da (average isotopes).

5.4.4 Synthesis of T-2S$_b$-µMax20

The synthesis of the TAMRA-labeled double benzene stapled product, **T-2S$_b$-µMax20**, was performed in a similar manner as detailed in sections 5.4.1-5.4.3 starting with the segments **32** and **34**.

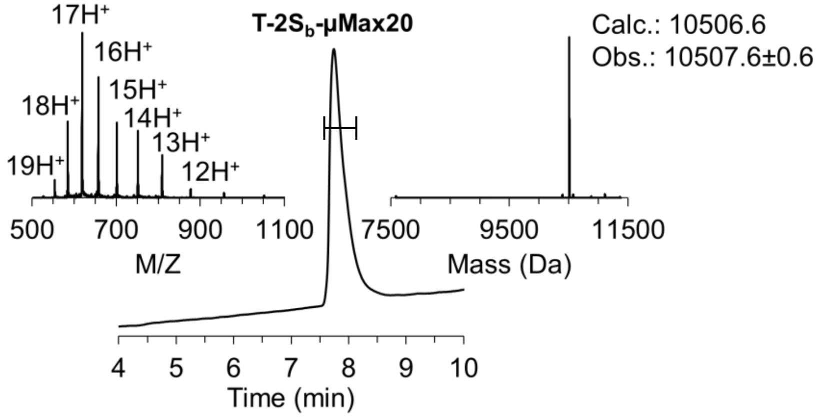

Figure S23. LCMS analysis of T-2S$_b$-µMax20. LC of the UV absorbance at 214 nm, mass-to-charge (M/Z) spectrum, and deconvoluted spectrum. LCMS analysis was carried out with Method A (see section 1.2). M/Z data was acquired over the marked region in the chromatogram. Calculated and observed masses are reported in Da (average isotopes).

5.5 Synthesis of T-Native Max

This protein was synthesized as previously reported.[8]

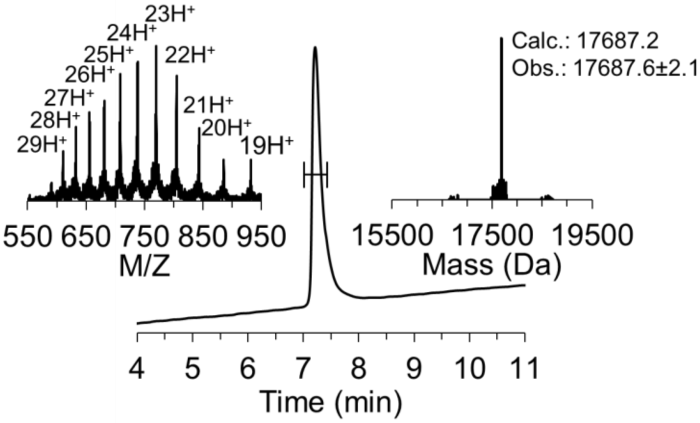

Figure S24. LCMS analysis of T-Native Max. LC of the UV absorbance at 214 nm, mass-to-charge (M/Z) spectrum, and deconvoluted spectrum. LCMS analysis was carried out with Method A (see section 1.2). M/Z data was acquired over the marked region in the chromatogram. Calculated and observed masses are reported in Da (average isotopes).

6. Chemical synthesis of Omomyc

6.1 Synthesis of Omomyc's segments

6.1.1 Synthesis of Segment Omo1 Omomyc(1-51)-$NHNH_2$

The synthesis was carried out according to the following scheme:

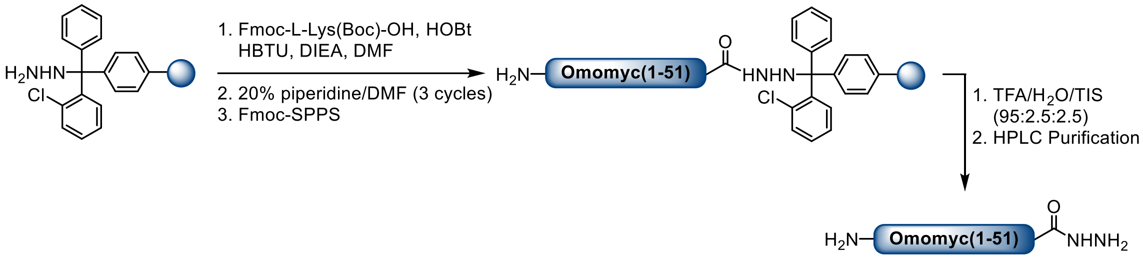

The synthesis of **Omo1** was carried out using stepwise Fmoc-SPPS chemistry on hydrazino resin (416.7 mg, loading 0.24 mmol/g, 0.1 mmol scale). The resin was coupled with Fmoc-L-Lys(Boc)-OH (5 equiv., 0.5 mmol, 166.7 mM), using HBTU (5 equiv., 0.5 mmol, 166.7 mM), HOBt (5 equiv., 0.5 mmol, 166.7 mM), and DIEA (10 equiv., 1.0 mmol, 333.3 mM) in 3 mL DMF for 45 minutes at RT. The resin was then transferred to the CSBio automated peptide synthesizer and the rest of the sequence was incorporated in a stepwise fashion using Fmoc-L-AA-OH (10 equiv., 1.0 mmol, 66.7 mM), HBTU/HOBt (10 equiv. each, 1.0 mmol, 66.7 mM), and DIEA (20 equiv., 2.0 mmol, 133.3 mM) in 15 mL DMF for 15 minutes at 60 °C per amino acid. Fmoc was deprotected after each coupling step by using three cycles of 20% piperidine and 0.05% F.A. in DMF (15 mL each) for 2, 4, and 2 minutes. Finally, the peptide-bound resin was washed with DMF (5 mL x 3), MeOH (5 mL x 3), and DCM (5 mL x 3) and dried under vacuum. To remove side chain protecting groups and release the peptide chains, a mixture of TFA/H_2O/TIS (95:2.5:2.5, 7 mL for 0.025 mmol scale) was added to the resin and shaken for 4 hours at RT. The resin was removed by filtration and extracted with TFA (2 x 1 mL). To precipitate the peptide, the filtrate was added dropwise to cold diethyl ether (35 mL for 0.025 mmol resin) followed by centrifugation at 4000 rpm for 7 min. Then, the diethyl ether was decanted, followed by the dissolution of the peptide in 50% ACN/water, dilution to 25% ACN/water, and lyophilization to acquire a white powder. The dry crude powder was purified by RP-HPLC (Method A described in Section 1.3) affording the product as white powder in the following yield: **Omo1** (36.6 mg, 5.91 µmol, 12% yield based on 0.05 mmol resin).

6.1.2 Synthesis of segment Omo2 Cys-Omomyc(53-92)

The synthesis was carried out according to the following scheme:

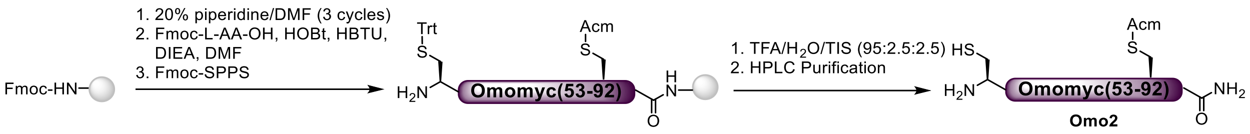

The synthesis of **Omo2** was carried out using stepwise Fmoc-SPPS chemistry on Rink amide Protide[TM] resin (277.8 mg, loading 0.18 mmol/g, 0.05 mmol scale). The resin was pre-swelled in DMF for 30 min, and the first 10 amino acids were coupled using Fmoc-L-AA-OH (5 equiv., 0.25 mmol, 125.0 mM), using HBTU (5 equiv., 0.25 mmol, 125.0 mM), HOBt (5 equiv., 0.25 mmol, 125.0 mM), and DIEA (10 equiv., 0.5 mmol, 250.0 mM) in 2 mL DMF for 15 minutes at RT. Cys91 was coupled as Fmoc-L-Cys(Acm)-OH. The resin was then transferred to the CSBio automated peptide synthesizer and the rest of the sequence was incorporated in a stepwise fashion using Fmoc-L-AA-OH (10 equiv., 0.5 mmol, 33.3 mM), HBTU/HOBt (10 equiv. each, 0.5 mmol, 33.3 mM), and DIEA (20 equiv., 1.0 mmol, 66.7 mM) in 15 mL DMF for 45 minutes at 30 °C per amino acid. Fmoc was deprotected after each coupling step by using three cycles of 20% piperidine and 0.05% F.A. in DMF (15 mL each) for 2, 4, and 2 minutes. Finally, the peptide-bound resin was washed with DMF (5 mL x 3), MeOH (5 mL x 3), and DCM (5 mL x 3) and dried under vacuum. To remove side chain protecting groups and release the peptide chains, a mixture of TFA/H_2O/TIS (95:2.5:2.5, 6 mL for 0.025 mmol scale) was added to the resin and shaken for 4 hours at RT. The resin was removed by filtration and extracted with TFA (2 x 1 mL). To precipitate the peptide, the filtrate was added dropwise to cold diethyl ether (30 mL for 0.025 mmol resin) followed by centrifugation at 4000 rpm for 7 min. Then, the diethyl ether was decanted, followed by the dissolution of the peptide in 50% ACN/water, dilution to 25% ACN/water, and lyophilization to acquire a white powder. The dry crude powder was purified by RP-HPLC (Method A described in Section 1.3) affording the product as a white powder in the following yield: **Omo2** (19.2 mg, 3.98 µmol, 8% yield).

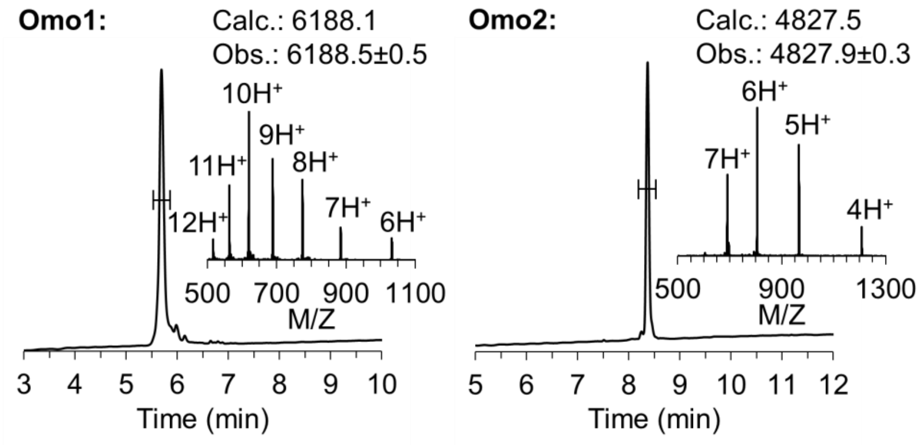

Figure S25. LCMS analysis of Omo1 and Omo2. LC of the UV absorbance at 214 nm and mass-to-charge (M/Z) spectrum. LCMS analysis was carried out with Method A (see section 1.2). M/Z data were acquired over the marked regions in the chromatograms. Calculated and observed masses are reported in Da (average isotopes).

6.2 Synthesis of Omomyc via one-pot native chemical ligation and desulfurization followed by Cys decaging

The reaction was carried out according to the following scheme:

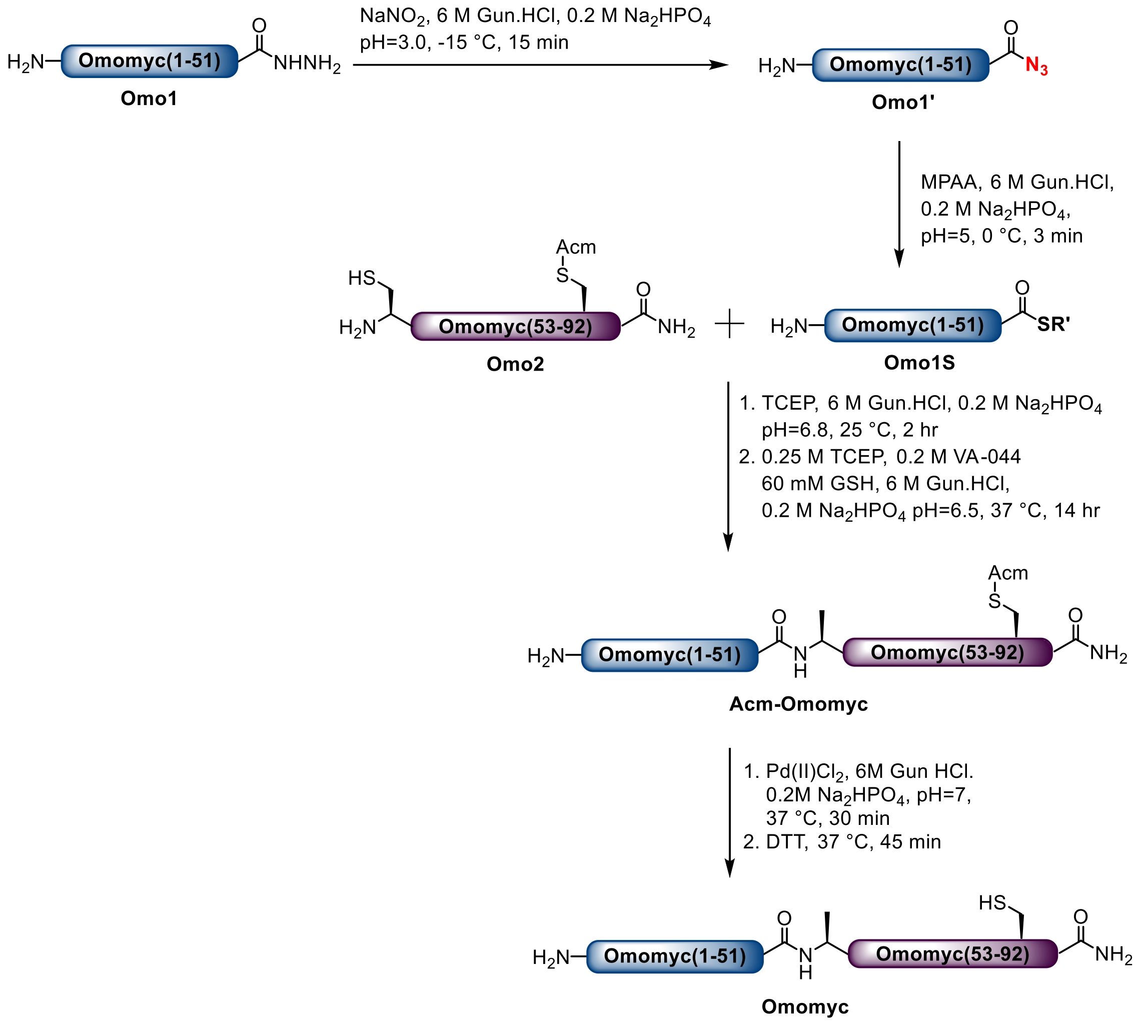

In a 1.5 mL Eppendorf, segment **Omo1** (18.5 mg, 1.3 equiv., 2.99 µmol) was dissolved in 350 µL of NCL buffer at pH=3.0 and cooled down to -15 °C by placing it in an ice/salt bath. 50 µL of $NaNO_2$ (10 equiv. based on **Omo1**, 29.9 µmol, 0.6 M) dissolved in water was added to the reaction mixture and allowed to react for 15 minutes at -15 °C with gentle mixing in repeated intervals. After 15 minutes, 350 µL of MPAA (50 equiv. based on **Omo1**, 0.15 mmol, 0.43 M) in NCL buffer at pH=7 were added to the mixture and gently mixed for two-three minutes. The segment **Omo2** (10.7 mg, 1 equiv., 2.3 µmol) was then dissolved in the reaction mixture, and the pH was adjusted to 6.8 using 6 *N* NaOH at 0 °C. The final concentration of the segments was: (**Omo1**) 3.9 mM and (**Omo2**) 3 mM. The mixture was incubated for 1.5 hours at 25 °C, and then 350 µL of TCEP (40

equiv. based on **Omo1**, 0.12 mmol, 0.34 M) in NCL buffer at pH=6.8 were added and continued incubating for 30 minutes at 25 °C. The reaction was monitored using LCMS (Method A described in Section 1.2). The ligation was completed in 2 hours. After completion of the reaction, the crude reaction was desalted by pipetting the reaction mixture into a 10 kDa molecular weight cutoff spin filter (Amicon® Ultra- 2mL, 10K). The reaction mixture was diluted with NCL buffer (pH=7.2) to 2.0 mL and concentrated to 1.0 mL by centrifuging the spin filter at 5000 rpm for 15 minutes. This process was repeated four more times. In the final process, the reaction mixture was concentrated to 1 mL. After that, the reaction mixture was collected by reverse centrifuge and then treated with VA-044 (0.2 mmol, 200 mM), TCEP (0.25 mmol, 250 mM), and GSH (60 µmol, 60 mM) for 12 hours. The progress of the reaction was monitored by LCMS (Method A in Section 1.2). After the completion of the reaction, purification was carried out using RP-HPLC (Method E described in Section 1.3), affording the caged product **Acm-Omomyc(1-92)** as white powder (13.8 mg, 1.26 µmol, 55% yield, based on the limiting segment **Omo2**).

The caged product **Acm-Omomyc(1-92)** was dissolved in 400 µL of NCL buffer (1 equiv., 13.8 mg, 1.26 µmol) and 250 µL of Pd(II)Cl_2 (10 equiv., 12.6 µmol, 50.4 mM) were added. The reaction solution was mixed and kept at 37 °C for 30 min. After that, 650 µL of DTT (50 equiv., 63.0 µmol, 99.0 mM) in NCL buffer were added to quench the reaction, and the solution was incubated at 37 °C for 45 min resulting in orange suspension. The progress of the reaction was monitored by LCMS (Method A in Section 1.2). Then, the reaction mixture was diluted with 0.1% TFA water to 2.5 mL and spun down to remove the precipitate. The filtrate was purified by RP-HPLC (Method A as described in section 1.3) to afford the decaged product **Omomyc** as a white powder (7.6 mg, 0.72 µmol, 55%).

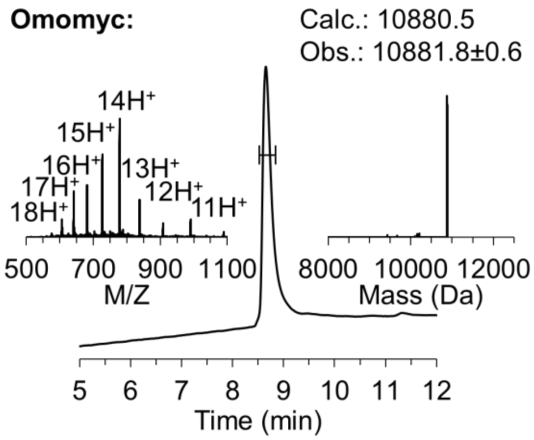

Figure S26. LCMS analysis of Omomyc. LC of the UV absorbance at 214 nm, mass-to-charge (M/Z) spectrum, and deconvoluted spectrum. LCMS analysis was carried out with Method A (see section 1.2). M/Z data was acquired over the marked region in the chromatogram. Calculated and observed masses are reported in Da (average isotopes).

7. DNA-Binding Analysis and Electrophoretic Mobility-Shift Assay (EMSA)

7.1 Single-point μMax analogs EMSA

To 0.2 mL Eppendorf tube, 16.0 μL of 10 mM MES, 150 mM KCl, 1 mM $MgCl_2$, 10% glycerol buffer pH=6.0 (folding buffer) were added (except for the well containing only DNA which in this case contained 18.0 μL of the folding buffer). E-box DNA probe (2.0 μL, 10.0 μM; 1 equiv.) as a solution in the folding buffer and **μMax1-19** analogs (2.0 μL, 30.0 μM; 3 equiv.) as a solution in the folding buffer were added.[8] The final incubation concentration of the major components was the following: DNA (1.0 μM) and protein (3.0 μM) in the folding buffer. The Eppendorf tube was closed, mixed, and incubated at room temperature for 30 minutes. Next, the DNA-binding activity of each analog was analyzed by EMSA: 5.0 μL of each DNA-protein mixture were mixed 1.0 μL DNA Loading Dye (6X). 5.0 μL of each mixture was loaded to a 10% TBE gel, 1.0 mm x 10 well and ran in 1xTBE at 90V for 75 min. The gel was washed three times with water for 30 seconds and then stained using Ethidium Bromide in 1xTBE buffer for 15 min at room temperature. Bands were visualized on A2S Vilber Fusion FX imager.

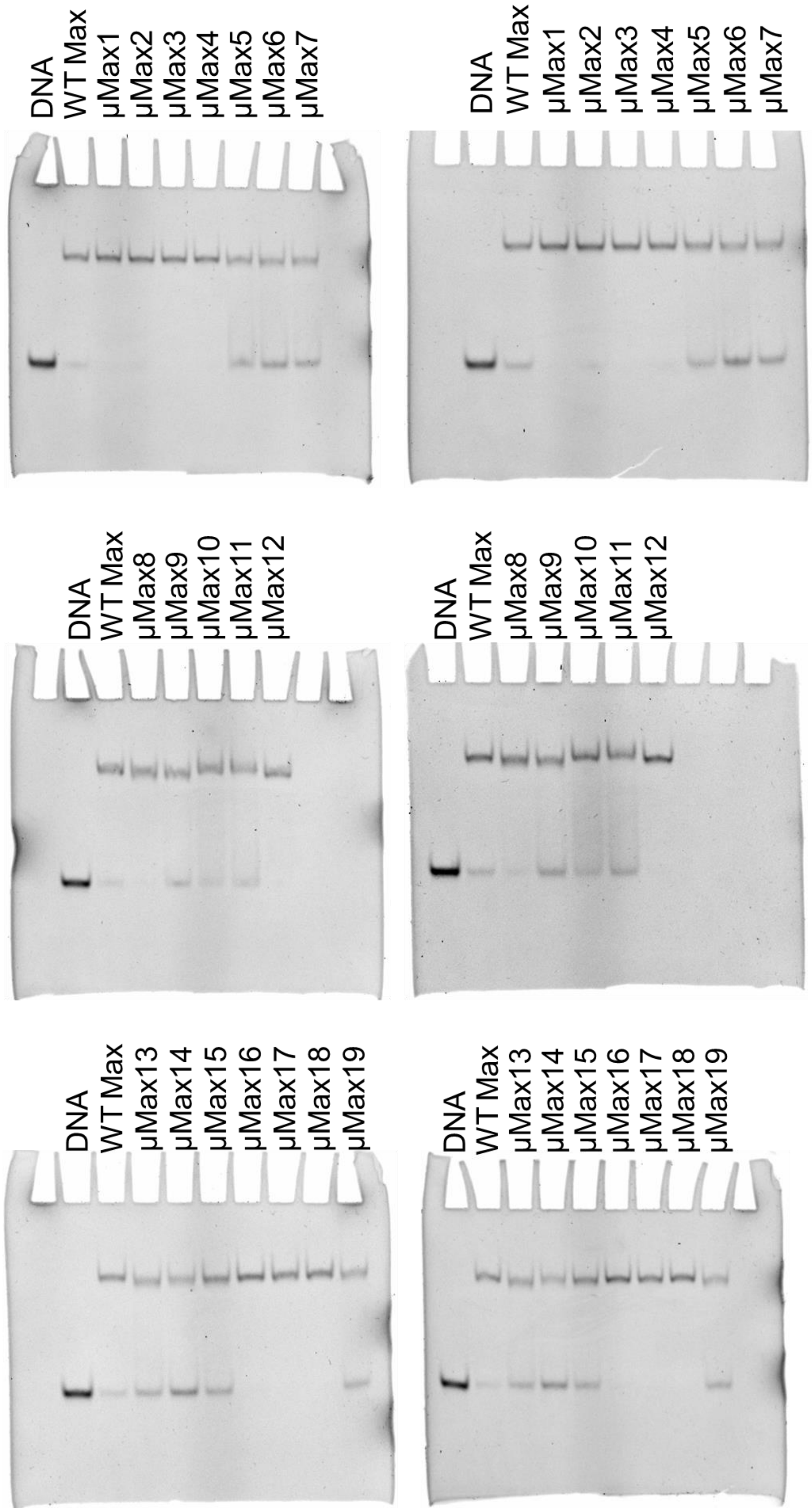

Figure S27. EMSA experiment of all single point mutated μMax analogs, with replicate. Conditions: 1 μM DNA probe and 3 μM protein in 10 mM MES, 150 mM KCl, 1 mM $MgCl_2$, 10% glycerol buffer pH=6.0.

7.2 Multi-point μMax analogs EMSA

To 0.2 mL Eppendorf tube, 25.0 μL or 24.0 μL folding buffer were added (except for the well containing only DNA which in this case contained 27.0 μL of the folding buffer). E-box DNA probe (3.0 μL, 10.0 μM; 1 equiv.) as a solution in the folding buffer and **μMax20-22** analogs (2.0 μL or 3.0 μL, 30.0 μM, 2 or 3 equiv. respectively) as a solution in the folding buffer were added. The final incubation concentration of the major components was the following: DNA (1.0 μM) and protein (2.0 μM or 3.0 μM) in the folding buffer. The Eppendorf tube was closed, mixed, and incubated at room temperature for 30 minutes. Next, the DNA-binding activity of each analog was analyzed by EMSA: 5.0 μL of each DNA-protein mixture were mixed 1.0 μL DNA Loading Dye (6X). 5.0 μL of each mixture was loaded to a 10% TBE gel, 1.0 mm x 10 well and ran in 1xTBE at 90V for 75 min. The gel was washed three times with water for 30 seconds and then stained using Ethidium Bromide in 1xTBE buffer for 15 min at room temperature. Bands were visualized on A2S Vilber Fusion FX imager.

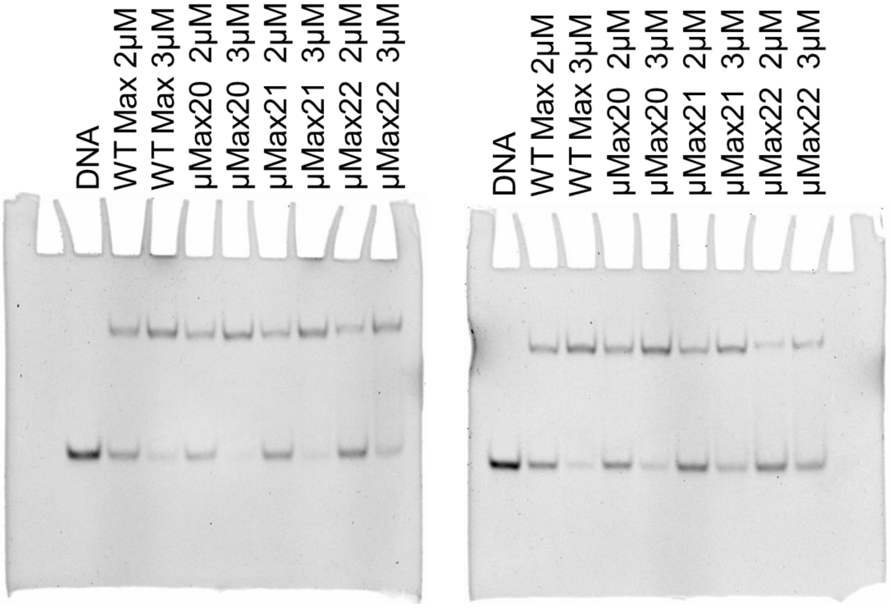

Figure S28. EMSA experiment of multi-point mutated analogs, with replicate. Conditions: 1 μM DNA probe and 2 or 3 μM protein in 10 mM MES, 150 mM KCl, 1 mM $MgCl_2$, 10% glycerol buffer pH=6.0.

7.3 Stapled μMax analogs EMSA

To 0.2 mL Eppendorf tube, 35.0, 34.0, 33.0, 32.0, or 31.0 μL folding buffer were added (except for the wells containing only DNA which in this case contained 36.0 μL of the folding buffer). E-box DNA probe (4.0 μL, 10.0 μM; 1 equiv.) as a solution in the folding buffer and **$2S_p$-μMax20** or **$2S_b$-μMax20** analogs (1.0, 2.0, 3.0, 4.0, or 5.0 μL, 40.0 μM, 1-5 equiv.) as a solution in the folding buffer were added. The final incubation concentration of the major components was the following: DNA (1.0 μM) and protein (1.0, 2.0, 3.0, 4.0, or 5.0 μM) in the folding buffer. The Eppendorf tube was closed, mixed, and incubated at room temperature for 30 minutes. Next, the DNA-binding activity of each analog was analyzed by EMSA: 5.0 μL of each DNA-protein mixture were mixed 1.0 μL DNA Loading Dye (6X). 5.0 μL of each mixture was loaded to a 10% TBE gel, 1.0 mm x 12 well and ran in 1xTBE at 90V for 75 min. The gel was washed three times with water for 30 seconds and then stained using Ethidium Bromide in 1xTBE buffer for 15 min at room temperature. Bands were visualized on A2S Vilber Fusion FX imager (Figure S29A).

To compare **μMax20**, **$2S_p$-μMax20**, and **$2S_b$-μMax20**, the same process was repeated to obtain final concentrations of 1.0 μM DNA and 2.0, 3.0, and 4.0 μM of each protein. Incubation time, gel loading, running, staining, and visualizing were performed as previously described (Figure S29B).

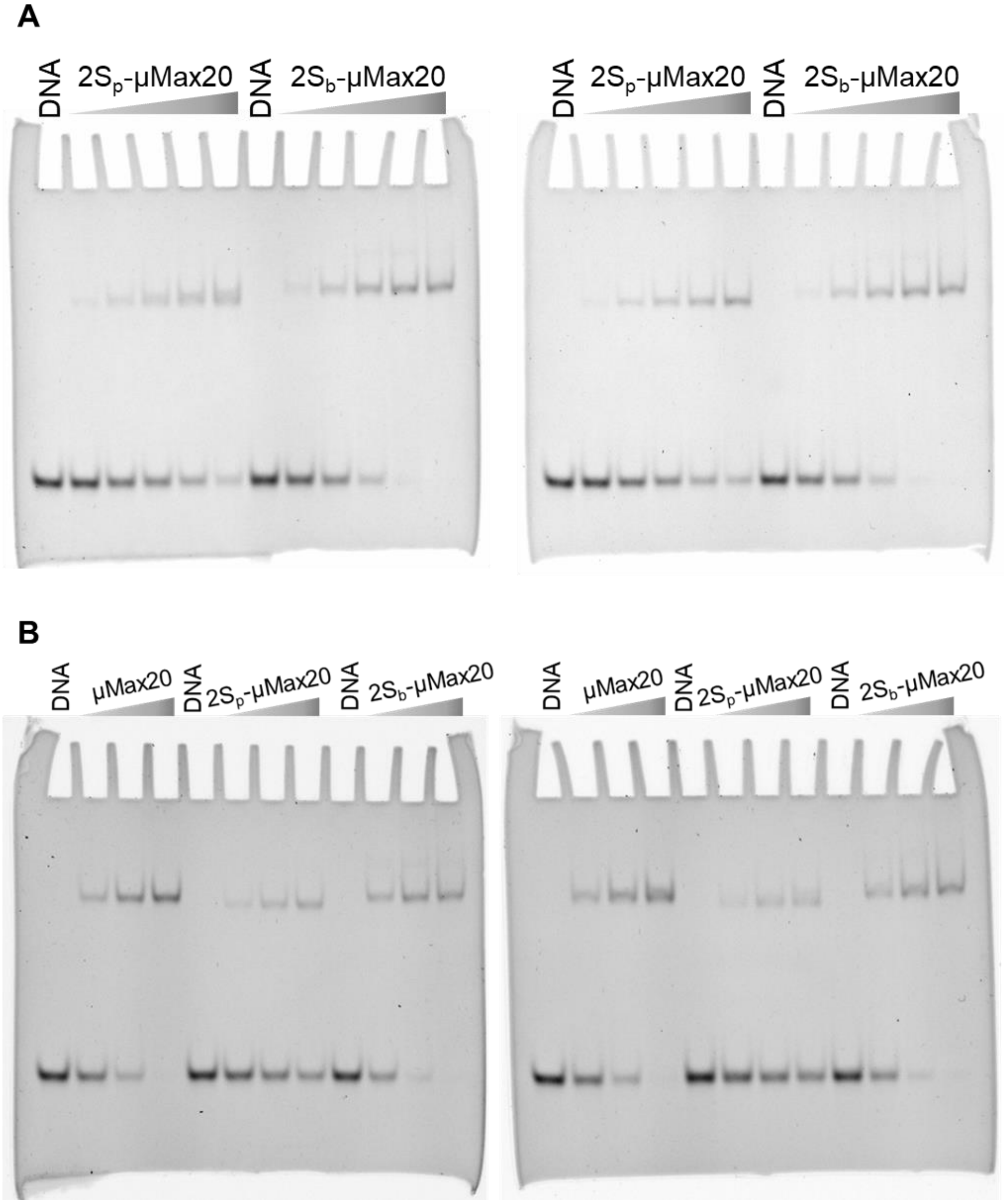

Figure S29. EMSA experiment of μMax20, $2S_p$-μMax20, and $2S_b$-μMax20, with replicate. A) Conditions: 1 μM DNA probe and 0-5 μM protein in 10 mM MES, 150 mM KCl, 1 mM $MgCl_2$, 10% glycerol buffer pH=6.0. B) Conditions: 1 μM DNA probe and 2-4 μM protein in 10 mM MES, 150 mM KCl, 1 mM $MgCl_2$, 10% glycerol buffer pH=6.0.

8. Circular dichroism (CD) analysis

CD analysis was carried out using Chirascan circular dichroism spectrometer with a 0.1 mm path length quartz cuvette. 10 μM, 50.0 μL protein solution was prepared in 10 mM MES, 150 mM KCl, 1 mM $MgCl_2$, 10% glycerol, and 0.5% DMSO buffer (pH=6.0). CD spectra of all samples were recorded in triplicates at 20 °C from 180 nm to 270 nm in 1.0 nm step with 3.0 nm slit bandwidth, and three seconds averaging times at each wavelength.

9. Protein binding microarray analyses

PBM experiments were performed as described previously[9–13] except we were detecting the fluorescence directly from the TAMRA tag on the protein, instead of the antibody. Universal PBM[10,11] microarrays were used (Agilent Technologies). The universal PBM used in this study along with the statistical analysis (E-score and Z-score) is the same as described before,[10,11] with 43774 probes of 60-bp long DNA sequences, providing binding statistics information for all 8bp sequences.

The microarrays were double stranded by solid-phase primer extension with Thermo Sequenase DNA Polymerase (Cytiva) and regular dNTP. After blocking with 2% non-fat dry milk, microarrays were incubated with fluorescent Max analogs (**T-WT Max**, **T-μMax20**, **T-μMax21**, **T-2S$_p$-μMax20**, and **T-2S$_b$-μMax20**). The protein concentration was in the range of 50 nM and 200 nM; the protein mixture is PBS based, containing 2% non-fat dry milk, 200 ng/μL BSA, 50 ng/μL Salmon Testes DNA, and 0.02% TX-100. The fluorescence was scanned with a GenePix® 4400A scanner at 532 nm at 2.5 μm resolution, and the fluorescence intensity was extracted with GenePix software for all probes in the array. All probes were randomized on the array. For each probe, we report the median pixel intensity. The experiment was performed in independent duplicates.

Z-score correlations of 8mers:

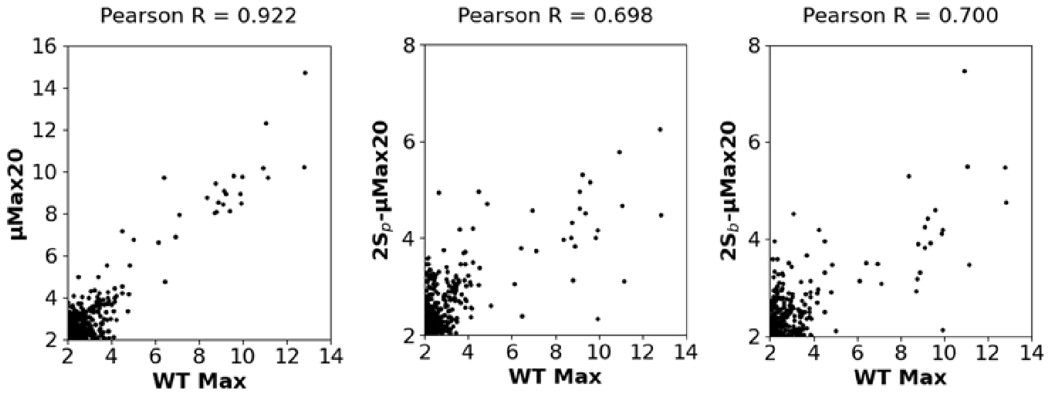

Figure S30. The correlation between the 8mers Z-score values of modified μMax20 analogs and WT Max.

Correlation of replicates:

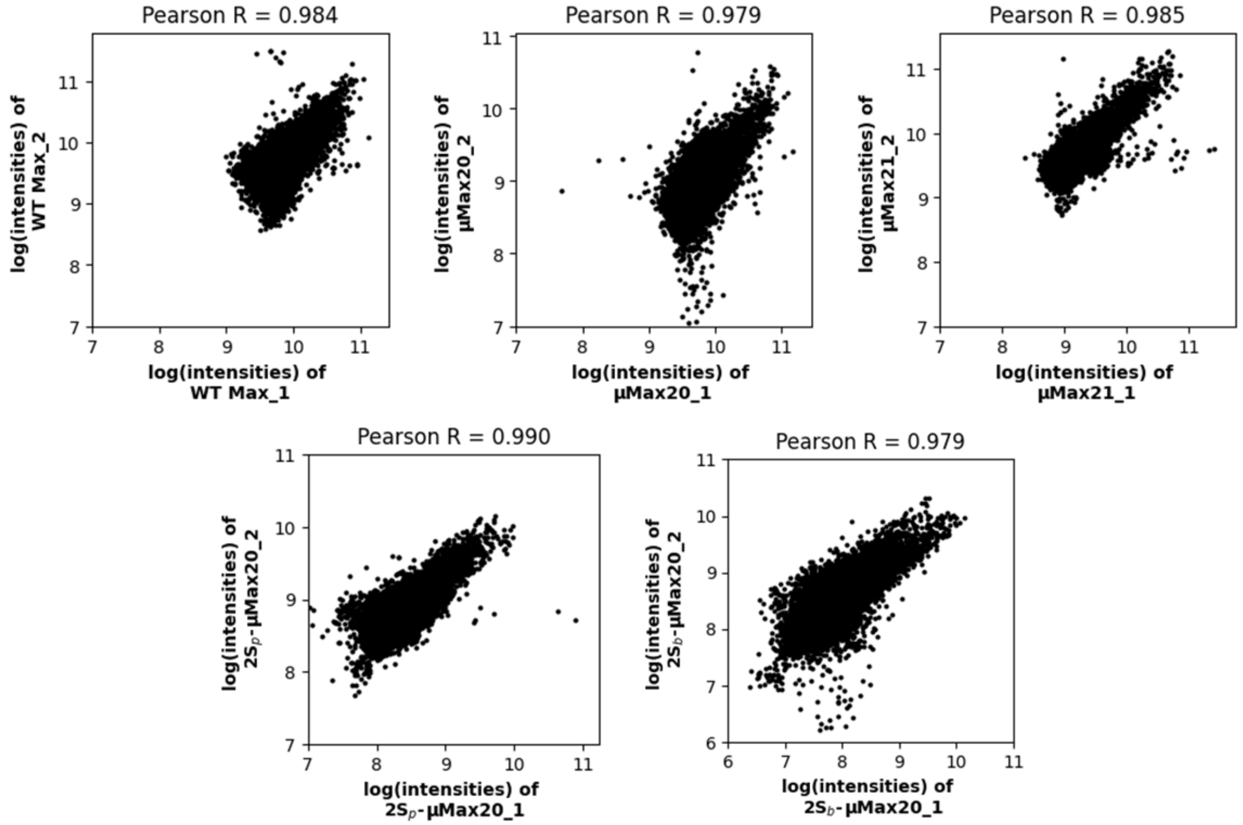

Figure S31. The correlation between the 8mers log(intensities) values of μMax analogs duplicates demonstrates reproducibility of the results.

Boxplots of analog's 8mer top binder compared to all other deBruijn sequences:

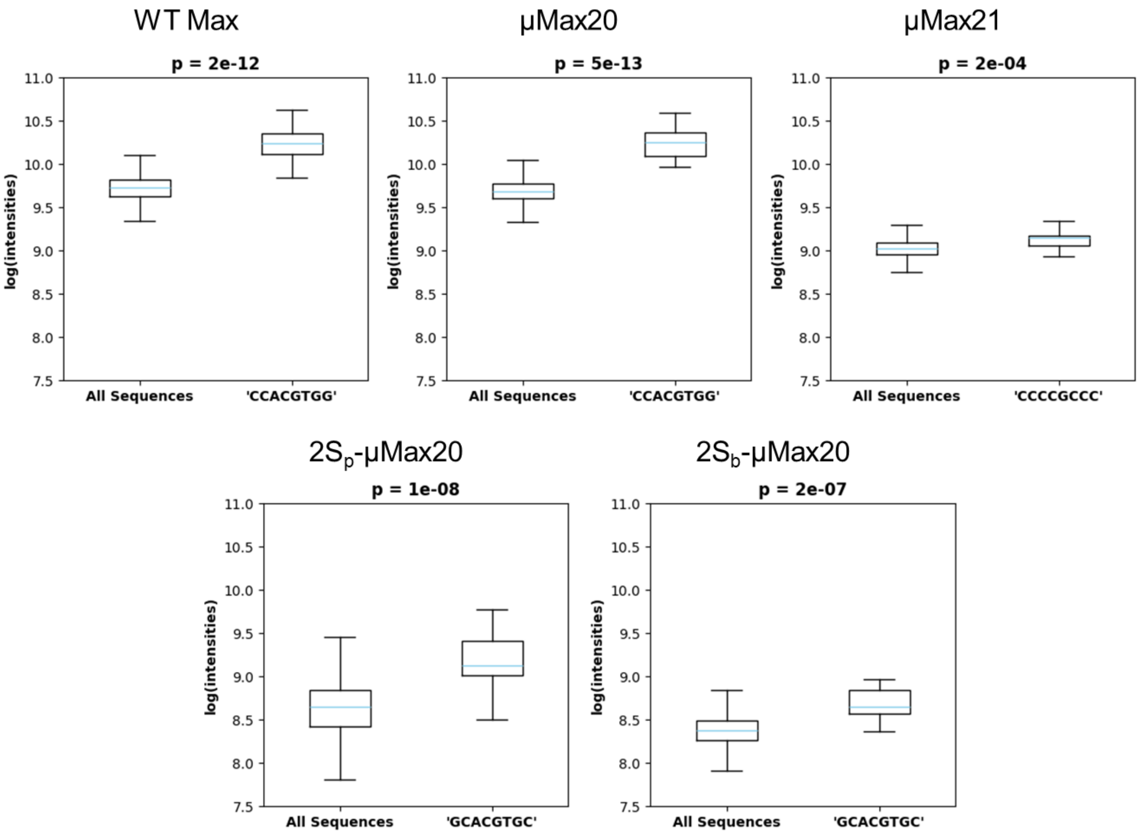

Figure S32. Boxplots comparing each analog's top binding k-mer probes intensity to the intensity of all other deBruijn sequences. The plots demonstrate that the preferred sequences are bound with substantially higher intensity than the background.

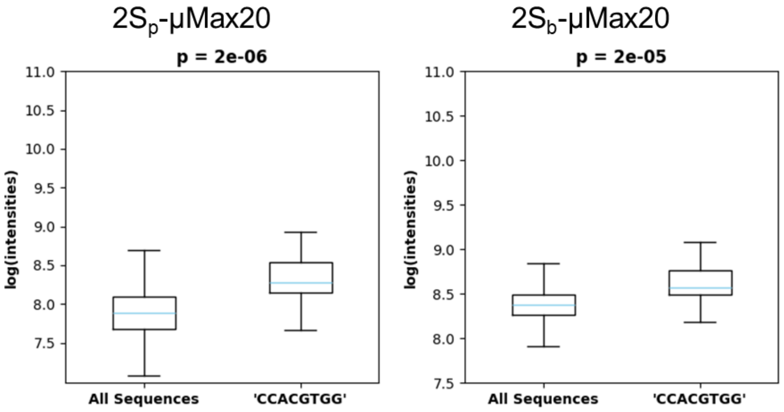

Figure S33. Boxplots comparing the intensity of CCACGTGG-containing probes bound by stapled analogs to that of all other deBruijn sequences. The plots demonstrate that this sequence is preferred with substantially higher intensity than the background.

10. Octet biolayer interferometry binding assay (BLI)

Biolayer interferometry (BLI) assays were performed using an Octet R4 System (ForteBio; Menlo Park, CA) in 96 well plates. Streptavidin Octet biosensors (ForteBio; Menlo Park, CA) were dipped into 200 µL of 0.1% BSA, 0.02% Tween-20, 1x PBS (kinetic buffer) for 10 minutes. Upon 10 minutes, the tips were dipped again in 200 µL of the kinetic buffer to acquire the baseline (60 seconds). Then, the tips were dipped into 200 µL of 65 nM biotinylated E-box DNA probe in the kinetic buffer for the loading step (300 seconds). Sensors were then dipped into the kinetic buffer for 60 seconds. Next, the tips were loaded with either **WT Max** or **µMax20** prepared in kinetic buffer at indicated concentrations for 300 seconds to obtain the association curve. Finally, the tips were dipped into the kinetic buffer for 600 seconds to obtain the dissociation curve. Measurements were carried out at 25 °C. Data was analyzed within the ForteBio Data Analysis software. The association and dissociation curves are fitted with Fortebio Biosystems (global fitting algorithm, 1:1 binding model) to obtain the kinetic K_D.

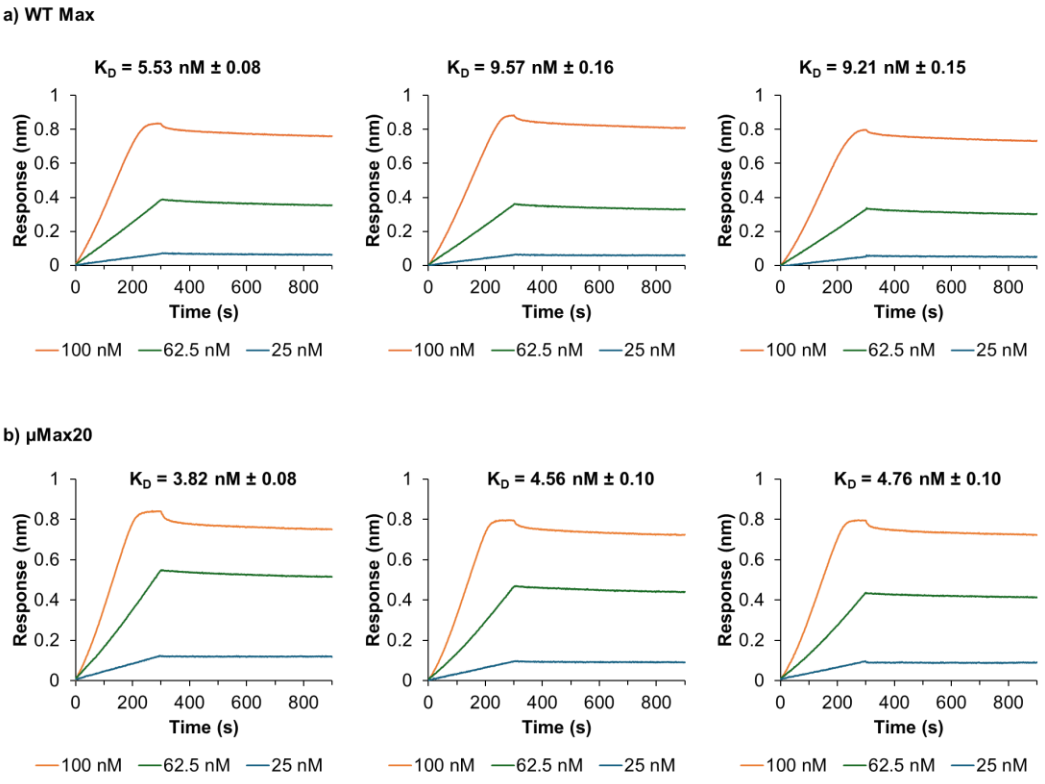

Figure S34. BLI analysis of Max analogs with the E-box DNA probe. The association and dissociation curves of proteins **WT Max** (a) and **µMax20** (b) with E-box DNA probe along with the triplicates.

11. Proteolytic Stability Assays

WT Max, **2S$_p$-μMax20**, and **2S$_b$-μMax20** were each dissolved in miliQ purified water to obtain 5.0 mg/mL stock solutions. Trypsin from bovine pancreas (SIGMA, Ref# T1426) was dissolved in miliQ purified water to obtain 1.0 μg/mL stock solution and put on ice for 5 minutes. 6.0 μL of protein solution was added to 94.0 μL solution of 50 mM Tris, 150 mM NaCl, and 10 mM $CaCl_2$ at pH=7.4 and put on ice for 5 minutes. Then, 0.67 μL of Trypsin solution was added to the protein solution, mixed, and incubated at 37 °C to initiate the proteolysis reaction. The reaction was quenched at the indicated time points by mixing 10.0 μL of the proteolysis solution with 20.0 μL of 30:70 H_2O:ACN containing 0.1% TFA.[14] The quenched solution was analyzed by comparing peaks areas using ThermoFischer Vanquish HPLC and Chromeleon™ software version 7.3.1. Fitting was done using GraphPad Prism 8.4.2. The experiment was performed in independent triplicates.

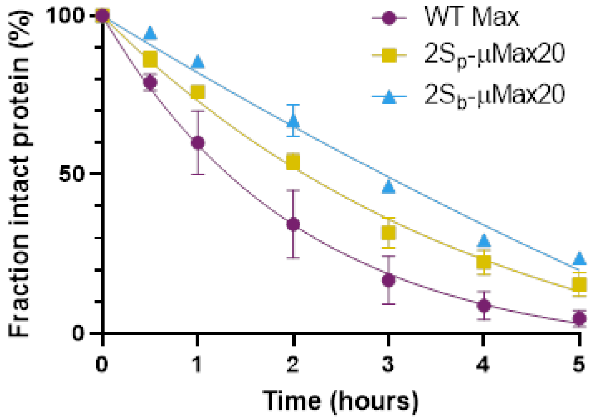

Figure S35. Stapled μMax20 analogs exhibit higher proteolytic stability compared to WT Max. The proteolytic degradation of **WT Max**, **2S$_p$-μMax20**, and **2S$_b$-μMax20** revealed half-lives of 1.3, 2.1, and 3.1 hours, respectively.

12. Cell permeability and nuclear localization assays

HeLa cells (ATCC, Ref# CCL-2) were grown in Dulbecco's modified Eagle's medium (DMEM, Gibco, Ref# 219699-035) supplemented with 10% (v/v) fetal bovine serum (Gibco, Ref# A56695-02), 20 mM L-glutamine (Gibco, Ref# 25030-024), and 1% (v/v) penicillin–streptomycin (Gibco, Ref# 15140-122). HeLa cells were resuspended in growth medium containing 10% (v/v) FBS, counted using a Countess II FL (Invitrogen™), plated (5,000 cells well^{-1}) in a µ-slide 18-well glass-bottom chamber (ibidi®), and incubated at 37 °C with 5% CO2 for 24 hours. Afterwards, the medium was removed by suction and replaced with 100 µL of phenol red-free media containing Max analogs labeled with the TAMRA fluorophore (**T-Native Max**, **T-WT Max**, **T-µMax20**, **T-2S$_p$-µMax20**, and **T-2S$_b$-µMax20**) at three different concentrations (0.25 µM, 1 µM and 4 µM). Samples were then incubated for 2 hours at 37 °C in a humidified 5% CO_2 atmosphere. Post-incubation, the wells were washed with fresh phenol red-free media containing the nuclear stain Hoechst 33342 (ThermoFisher, Ref# H1399) at 1 µg mL^{-1}, prior to imaging. Cells were imaged at 37 °C in a Leica SP8 fluorescence confocal microscope equipped with a live-cell imaging stage using a HC PL APO CS2 20x/0.75 dry lens with a resolution of 1024x1024 pixels. The microscope was configured to detect Hoechst 33342 and TAMRA using an automatic Dye Wizard configuration (Exc./Em. 405/461 nm and 552/578 nm, respectively). Images were acquired and processed with the corresponding microscope software, Leica Application Suite X (LAS X) V1.4.6. Quantification of cellular internalization was performed by measuring the intracellular TAMRA fluorescence normalized to Hoechst nuclear signal. Mean fluorescence values were calculated using FIJI from three independent fields of view per condition. Data are presented as mean ± SD, and data analysis was performed using GraphPad Prism 5.0.

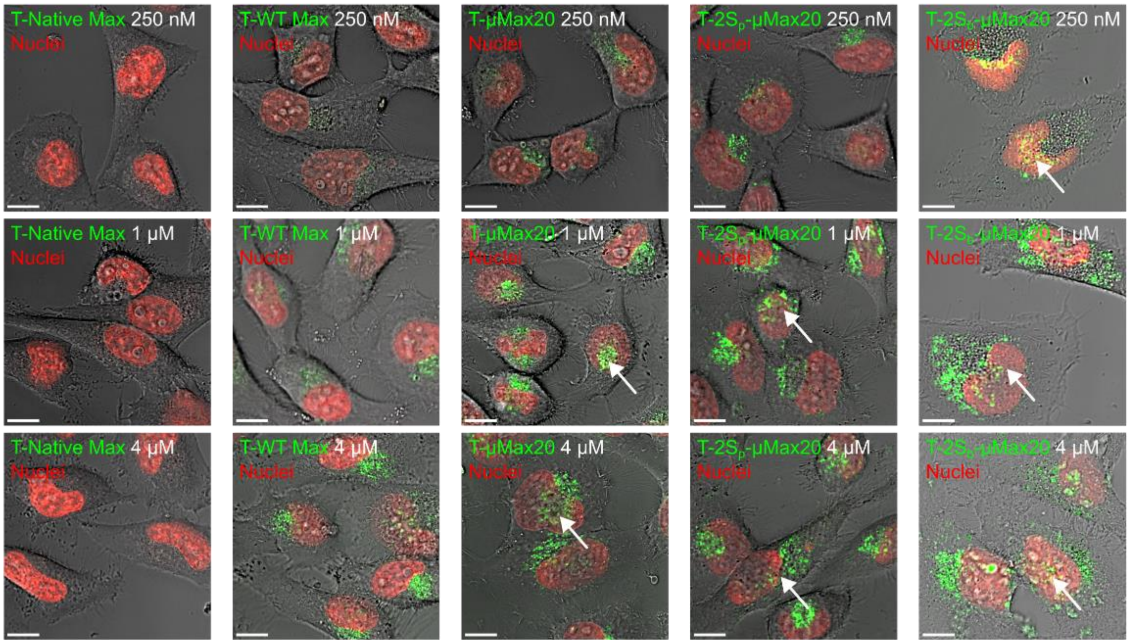

Figure S36. Abiotic μMax20 analogs enhance cellular uptake and nuclear localization compared to native Max analogs. Representative fluorescence microscopy images of HeLa cells treated with TAMRA-labeled Max analogs (**T-Native Max**, **T-WT Max**, **T-μMax20**, **T-2S$_p$-μMax20**, and **T-2S$_b$-μMax20**) at increasing concentrations: 250 nM (top), 1 μM (middle), and 4 μM (bottom). Brightfield images were merged with TAMRA (green; Exc./Em. 552/578 nm) and Hoechst 33342 fluorescence signals (red; Exc./Em. 405/461 nm). White arrows indicate nuclear accumulation of μMax-based analogs. Scale bars: 10 μm.

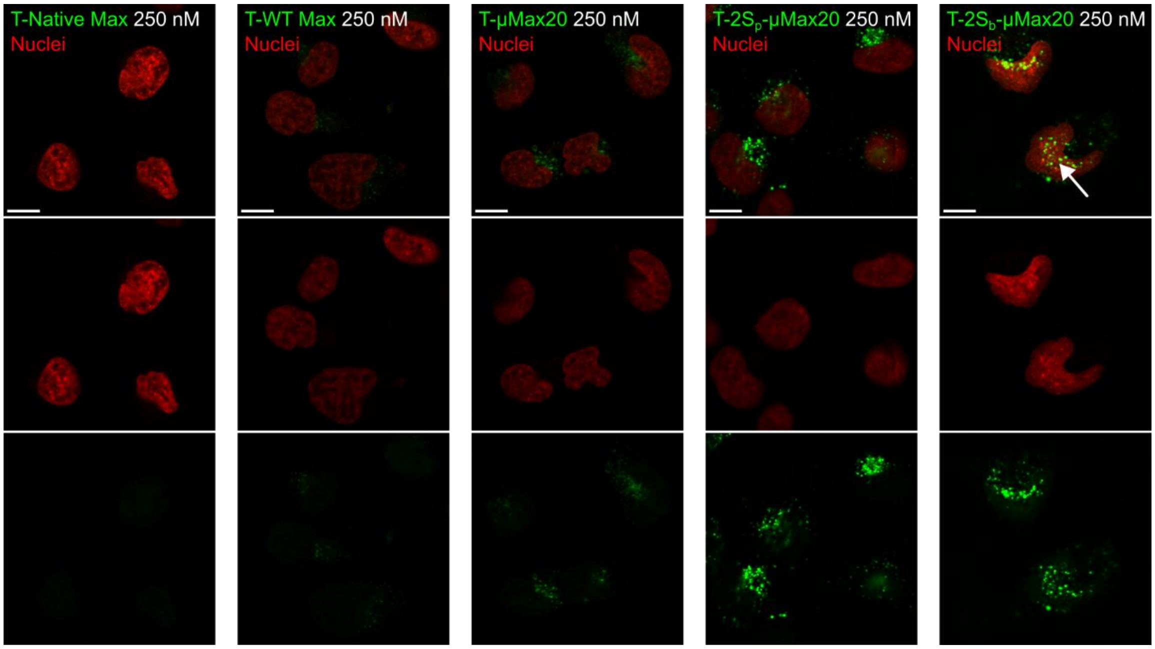

Figure S37. Single-channel fluorescence images of HeLa cells treated with TAMRA-labeled Max analogs at 250 nM. Merged images (top) of Hoechst 33342 (red; Exc./Em. 405/461 nm; middle) and TAMRA fluorescence signals (green; Exc./Em. 552/578 nm; bottom). Scale bars: 10 μm.

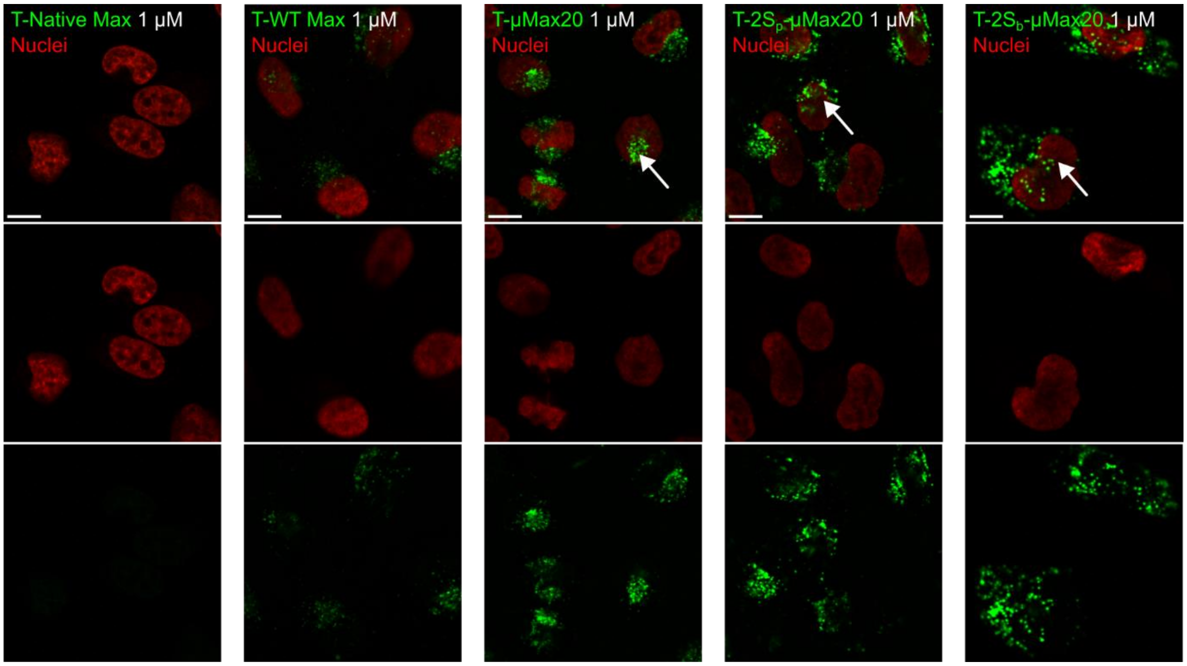

Figure S38. Single-channel fluorescence images of HeLa cells treated with TAMRA-labeled Max analogs at 1 μM. Merged images (top) of Hoechst 33342 (red; Exc./Em. 405/461 nm; middle) and TAMRA fluorescence signals (green; Exc./Em. 552/578 nm; bottom). Scale bars: 10 μm.

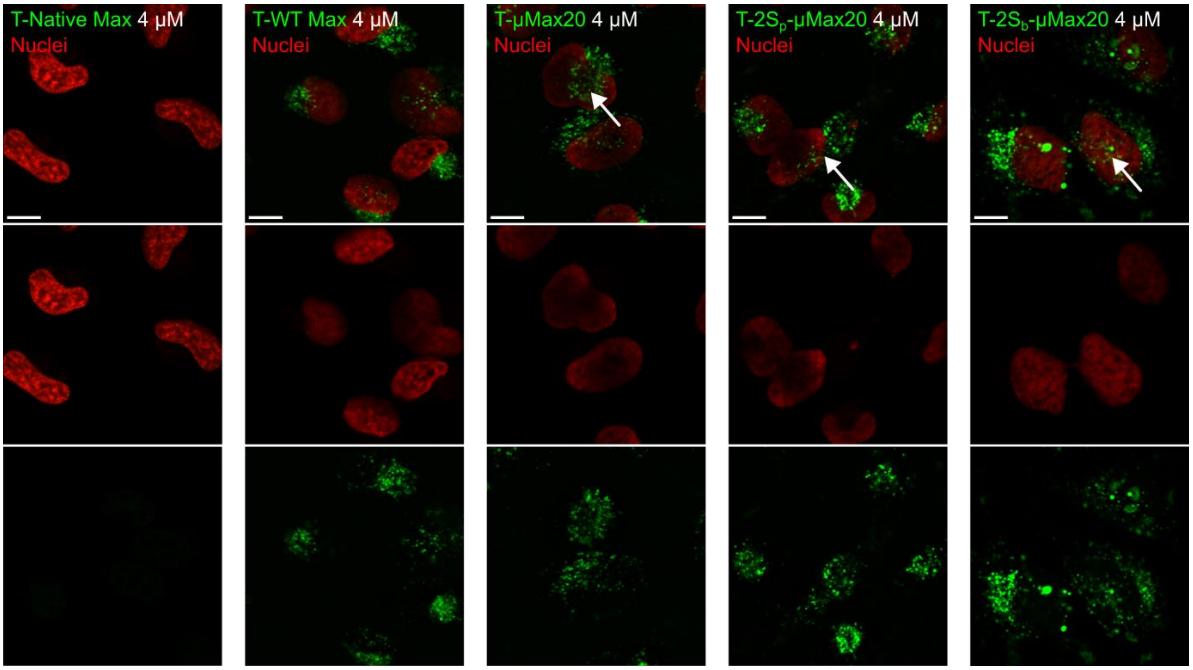

Figure S39. Single-channel fluorescence images of HeLa cells treated with TAMRA-labeled Max analogs at 4 μM. Merged images (top) of Hoechst 33342 (red; Exc./Em. 405/461 nm; middle) and TAMRA fluorescence signals (green; Exc./Em. 552/578 nm; bottom). Scale bars: 10 μm.

Z-stack imagining microscopy. For Z-stack imaging microscopy, 40 images were acquired across a physical depth of ~25 μm. 3D representations were processed with the corresponding microscope software, Leica Application Suite X (LAS X) V1.4.6.

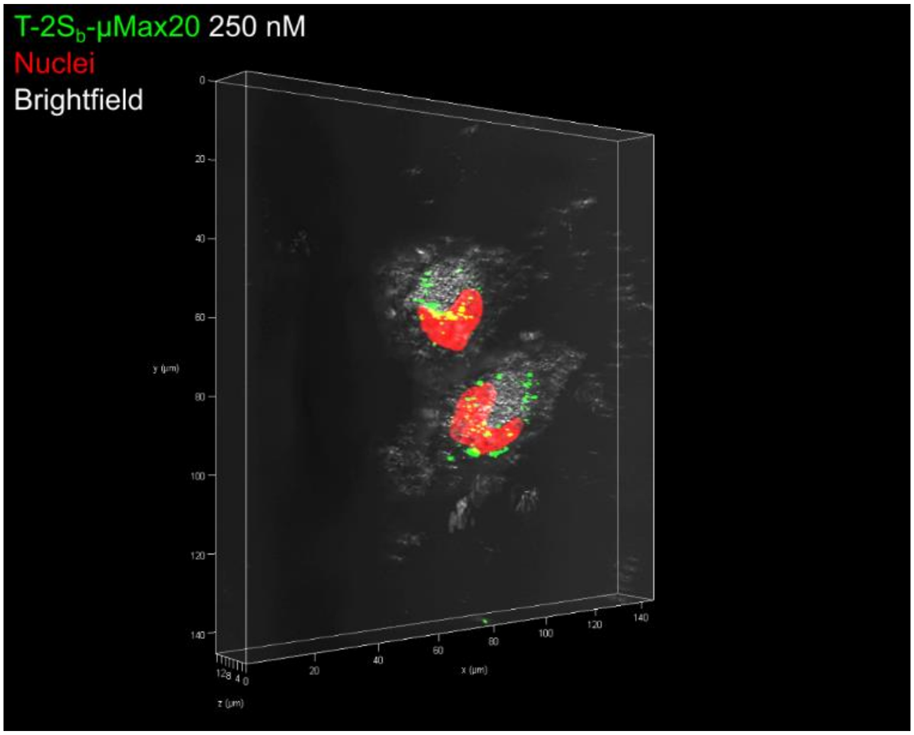

Figure S40. Z-stack imaging experiments of T-2S$_b$-μMax20. Representative fluorescence microscopy 3D reconstructions of HeLa cells treated with **T-2S$_b$-μMax20** (250 nM). 40 images were acquired across a physical depth of ~25 μm. The brightfield image was merged with TAMRA (green; Exc./Em. 552/578 nm) and Hoechst 33342 fluorescence signals (red; Exc./Em. 405/461 nm). The corresponding movie for the 3D reconstruction is included in the Supporting Files (Movie S1).

13. Cellular uptake mechanism analysis

To inhibit endocytosis, cells were pre-treated with MiTMAB™ (15 μM) for 15 minutes at 37 °C in a humidified 5% CO_2 atmosphere. Following this incubation, **T-WT Max**, **T-μMax20**, **T-2S$_p$-μMax20**, and **T-2S$_b$-μMax20** (4 μM) were added to the cells. Endpoint images were acquired as described in section 12 after incubation for 2 hours at 37 °C in a humidified 5% CO_2 atmosphere.

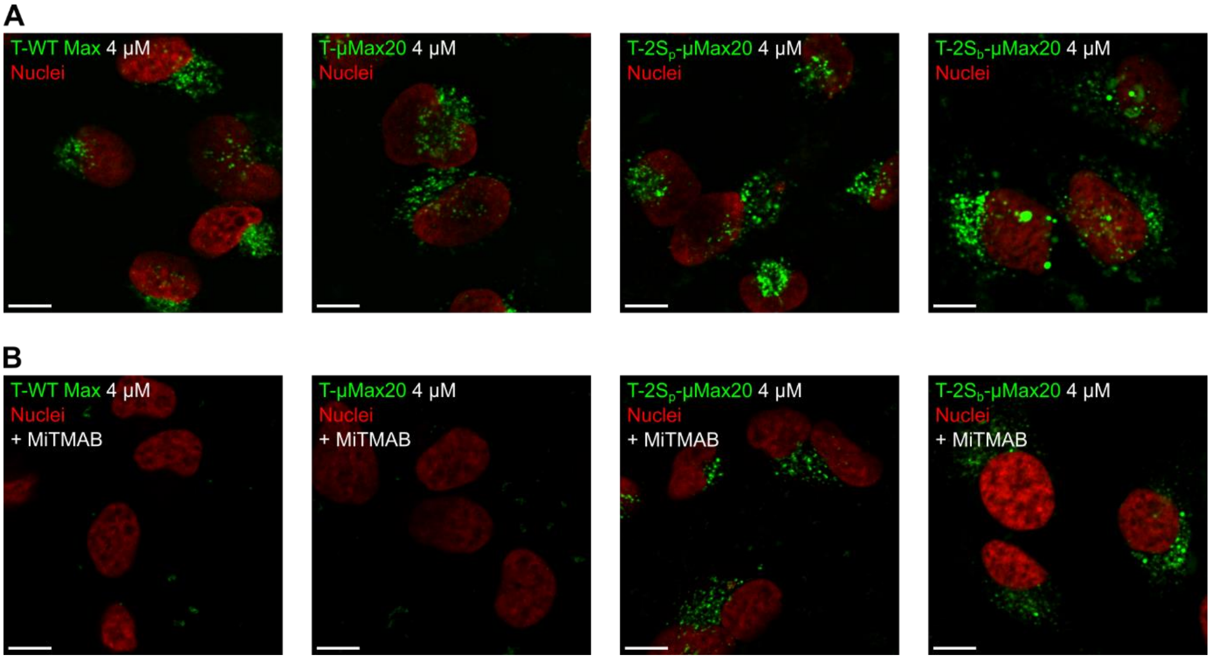

Figure S41. Stapled abiotic μMax analogs exhibit dual cell penetration mechanisms. A) Representative fluorescence confocal microscopy images of live HeLa cells after treatment with **T-WT Max**, **T-μMax20**, **T-2S$_p$-μMax20**, and **T-2S$_b$-μMax20** (4 μM) without the endocytosis inhibitor MiTMAB. B) Representative fluorescence confocal microscopy images of live HeLa cells after treatment with **T-WT Max**, **T-μMax20**, **T-2S$_p$-μMax20**, and **T-2S$_b$-μMax20** (4 μM) with the endocytosis inhibitor MiTMAB (15 μM). TAMRA fluorescence is shown in green (Exc./Em. 552/578), and nuclei are stained with Hoechst 33342 (red; Exc./Em. 405/461). Scale bars: 10 μm.

14. Myc reporter gene assays

HeLa cells were seeded at 10,000 cells per well in 100 µL of complete growth medium in a flat-bottom, transparent 96-well plate and incubated for 24 h at 37 °C with 5% CO_2. The following day, cells were transfected using the Cignal Myc Reporter Assay Kit (Qiagen, GeneGlobe ID: CCS-012L; Cat. No. 336841). For transfection, 12 µL of Lipofectamine 3000 (ThermoFisher) was diluted in 200 µL Opti-MEM (Gibco), while 40 µL of plasmid DNA (100 ng μL^{-1} stock) was diluted in 200 µL Opti-MEM to a final concentration of 20 ng μL^{-1}, followed by the addition of 4 µL P3000 reagent. Lipofectamine and DNA solutions were combined (1:1, 200 µL each), gently mixed, and incubated for 15 min at room temperature to allow complex formation. Then, 10 µL of the DNA-lipid complexes (containing 100 ng DNA) were added per well. Cells were incubated overnight at 37 °C with 5% CO_2. On the third day, the transfection medium was carefully removed and replaced with fresh complete medium and incubated for an additional 8 h. Subsequently, cells were treated or not with Max analogs (**WT Max**, **µMax20**, **2S$_p$-µMax20**, **2S$_b$-µMax20**) or the control (**Omomyc**) at final concentrations of 0.25, 1, or 4 µM, and incubated for 48 h at 37 °C with 5% CO_2. Luciferase activity was measured using the Dual-Glo® Luciferase Assay System (Promega, Cat. No. E2920) according to the manufacturer's protocol. Briefly, the plate was equilibrated to room temperature before the addition of 75 µL of Dual-Glo® Luciferase Reagent to each well. After 1 h incubation at room temperature, firefly luminescence was recorded using a microplate reader without filters. Subsequently, 75 µL of Dual-Glo® Stop & Glo® Reagent was added to each well, followed by another 1 h incubation prior to reading Renilla luminescence. Relative luciferase activity was determined by calculating the ratio of firefly (Myc reporter) to Renilla (control reporter) luminescence.

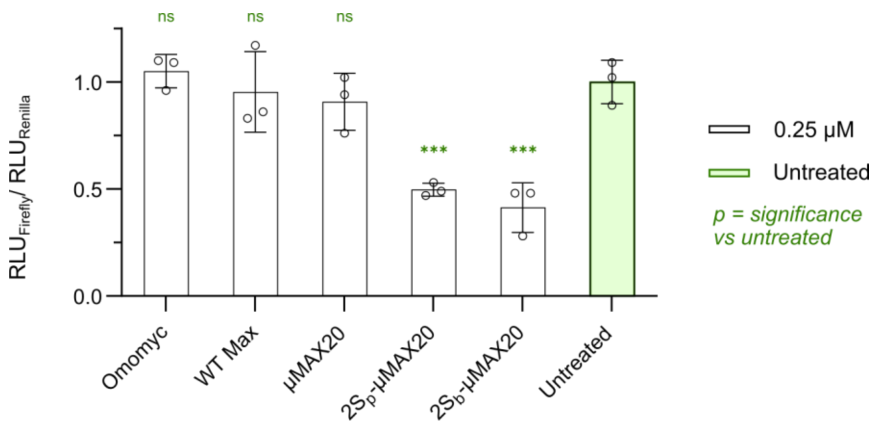

Figure S42. Inhibition of Myc transcriptional activity at submicromolar concentrations of Max analogs. HeLa cells transfected with a luciferase-based Myc reporter system and treated with 0.25 µM Max analogs for 48 h. Data represents the ratio of firefly luciferase expression (driven by c-Myc binding to E-box elements) to renilla luciferase (constitutively expressed), normalized to untreated cells. Data are shown as means ± SD from three independent experiments. Statistical significance relative to the untreated condition was calculated by one-way ANOVA and indicated in green: $p < 0.001$ (***), ns = not significant. Data are shown as means ± SD from three independent experiments.

15. Cell viability assays

HeLa cells were seeded at 2,500 cells per well in 100 µL complete growth medium in flat-bottom, transparent 96-well plates and incubated for 24 h at 37 °C with 5% CO_2. The following day, cells were treated or not with Max analogs (**WT Max**, **µMax20**, **2S$_p$-µMax20**, **2S$_b$-µMax20**) or the control (**Omomyc**) at final concentrations of 1, 4, or 16 µM. Cells were incubated for 72 h under standard conditions (37 °C, 5% CO_2). On day 4, cell viability was measured using a resazurin-based assay. Briefly, 10 µL of resazurin solution (1 mg mL^{-1} in PBS) was added to each well, and plates were incubated for 2 h at 37 °C. The fluorescent product of resazurin reduction (resorufin) was measured using a microplate reader (Ex/Em: 560/590 nm). Phosphate-buffered saline (PBS) and 0.01% (v/v) Triton X-100 were included as negative and positive controls, respectively.

16. References

(1) Zheng, J. S.; Tang, S.; Qi, Y. K.; Wang, Z. P.; Liu, L. Chemical Synthesis of Proteins Using Peptide Hydrazides as Thioester Surrogates. *Nature Protocols* **2013**, *8* (12), 2483–2495.

(2) Dawson, P. E.; Muir, T. W.; Clark-Lewis, I.; Kent, S. B. H. Synthesis of Proteins by Native Chemical Ligation. *Science (1979)* **1994**, *266* (5186), 776–779.

(3) Fang, G. M.; Li, Y. M.; Shen, F.; Huang, Y. C.; Li, J. Bin; Lin, Y.; Cui, H. K.; Liu, L. Protein Chemical Synthesis by Ligation of Peptide Hydrazides. *Angewandte Chemie International Edition* **2011**, *50* (33), 7645–7649.

(4) Wan, Q.; Danishefsky, S. J. Free-Radical-Based, Specific Desulfurization of Cysteine: A Powerful Advance in the Synthesis of Polypeptides and Glycopolypeptides. *Angewandte Chemie International Edition* **2007**, *46* (48), 9248–9252.

(5) Haase, C.; Rohde, H.; Seitz, O. Native Chemical Ligation at Valine. *Angewandte Chemie International Edition* **2008**, *47* (36), 6807–6810.

(6) Jo, H.; Meinhardt, N.; Wu, Y.; Kulkarni, S.; Hu, X.; Low, K. E.; Davies, P. L.; Degrado, W. F.; Greenbaum, D. C. Development of α-Helical Calpain Probes by Mimicking a Natural Protein-Protein Interaction. *J Am Chem Soc* **2012**, *134* (42), 17704–17713.

(7) Maity, S. K.; Jbara, M.; Laps, S.; Brik, A. Efficient Palladium-Assisted One-Pot Deprotection of (Acetamidomethyl)Cysteine Following Native Chemical Ligation and/or Desulfurization To Expedite Chemical Protein Synthesis. *Angewandte Chemie International Edition* **2016**, *55* (28), 8108–8112.

(8) Nithun, R. V.; Yao, Y. M.; Harel, O.; Habiballah, S.; Afek, A.; Jbara, M. Site-Specific Acetylation of the Transcription Factor Protein Max Modulates Its DNA Binding Activity. *ACS Cent Sci* **2024**, *10* (6), 1295–1303.

(9) Berger, M. F.; Bulyk, M. L. Universal Protein-Binding Microarrays for the Comprehensive Characterization of the DNA-Binding Specificities of Transcription Factors. *Nature Protocols* **2009**, *4* (3), 393–411.

(10) Berger, M. F.; Philippakis, A. A.; Qureshi, A. M.; He, F. S.; Estep, P. W.; Bulyk, M. L. Compact, Universal DNA Microarrays to Comprehensively Determine Transcription-Factor Binding Site Specificities. *Nature Biotechnology* **2006**, *24* (11), 1429–1435.

(11) Mukherjee, S.; Berger, M. F.; Jona, G.; Wang, X. S.; Muzzey, D.; Snyder, M.; Young, R. A.; Bulyk, M. L. Rapid Analysis of the DNA-Binding Specificities of Transcription Factors with DNA Microarrays. *Nature Genetics* **2004**, *36* (12), 1331–1339.

(12) Afek, A.; Shi, H.; Rangadurai, A.; Sahay, H.; Senitzki, A.; Xhani, S.; Fang, M.; Salinas, R.; Mielko, Z.; Pufall, M. A.; Poon, G. M. K.; Haran, T. E.; Schumacher, M. A.; Al-Hashimi, H. M.; Gordân, R. DNA Mismatches Reveal Conformational Penalties in Protein–DNA Recognition. *Nature* **2020**, *587* (7833), 291–296.

(13) Gordân, R.; Shen, N.; Dror, I.; Zhou, T.; Horton, J.; Rohs, R.; Bulyk, M. L. Genomic Regions Flanking E-Box Binding Sites Influence DNA Binding Specificity of BHLH Transcription Factors through DNA Shape. *Cell Rep* **2013**, *3* (4), 1093–1104.

(14) Mong, S. K.; Cochran, F. V.; Yu, H.; Graziano, Z.; Lin, Y. S.; Cochran, J. R.; Pentelute, B. L. Heterochiral Knottin Protein: Folding and Solution Structure. *Biochemistry* **2017**, *56* (43), 5720–5725.
